# Cationic molybdenum oxo alkylidenes stabilized by N-heterocyclic carbenes: from molecular systems to efficient supported metathesis catalysts†

**DOI:** 10.1039/d2sc03321f

**Published:** 2022-07-12

**Authors:** Janis V. Musso, Jordan De Jesus Silva, Mathis J. Benedikter, Jonas Groos, Wolfgang Frey, Christophe Copéret, Michael R. Buchmeiser

**Affiliations:** Institute of Polymer Chemistry, University of Stuttgart Pfaffenwaldring 55 D-70569 Stuttgart Germany michael.buchmeiser@ipoc.uni-stuttgart.de; Department of Chemistry and Applied Biosciences, ETH Zürich Vladimir-Prelog-Weg 1–5 CH-8093 Zürich Switzerland ccoperet@ethz.ch; Institute of Organic Chemistry, Universität Stuttgart Pfaffenwaldring 55 70569 Stuttgart Germany; German Institutes of Textile and Fiber Research (DITF) Denkendorf Körschtalstr. 26, 73770 Denkendorf Germany

## Abstract

Cationic d^0^ group 6 olefin metathesis catalysts have been recently shown to display in most instances superior activity in comparison to their neutral congeners. Furthermore, their catalytic performance is greatly improved upon immobilization on silica. In this context, we have developed the new family of molecular cationic molybdenum oxo alkylidene complexes stabilized by N-heterocyclic carbenes of the general formula [Mo(O)(CHCMe_3_)(IMes)(OR)[X^−^]] (IMes = 1,3-dimesitylimidazol-2-ylidene; R = 1,3-dimesityl-C_6_H_3_, C_6_F_5_; X^−^ = B(3,5-(CF_3_)_2_C_6_H_3_)_4_^−^, B(Ar^F^)_4_, tetrakis(perfluoro-*t*-butoxy)aluminate (PFTA)). Immobilization of [Mo(O)(CHCMe_3_)(IMes)(O-1,3-dimesityl-C_6_H_3_)^+^B(Ar^F^)_4_^−^] on silica *via* surface organometallic chemistry yields an active alkene metathesis catalyst that shows the highest productivity towards terminal olefins amongst all existing molybdenum oxo alkylidene catalysts.

## Abstract 2

The first cationic molybdenum oxo complexes were synthesized and immobilized on partially dehydroxylated silica. Vastly enhanced catalytic activity for terminal olefins was found compared to their neutral congeners.

## Graphical abstract

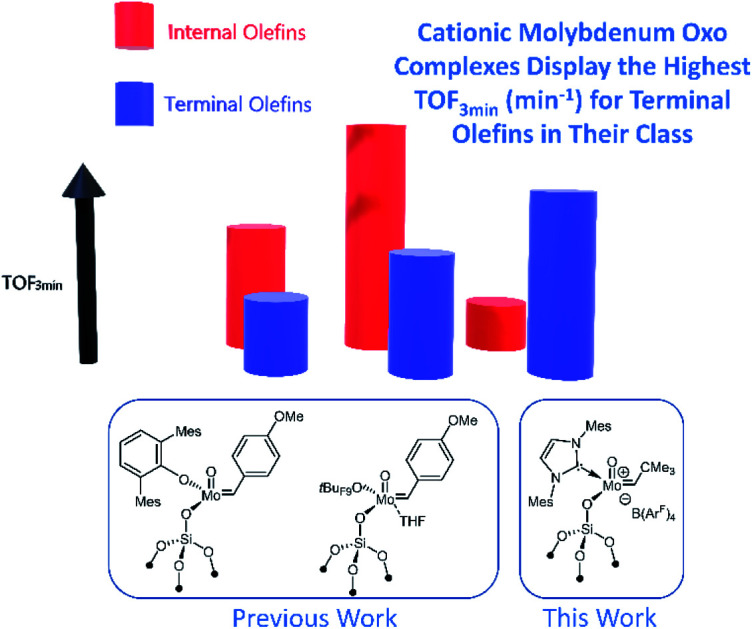

## Introduction

**Fig. 1:**
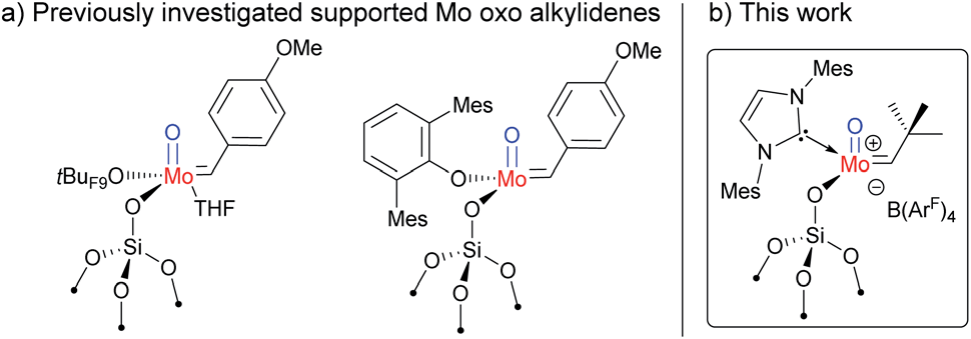
(a) Previously reported supported neutral Mo oxo alkylidene NHC species; (b) supported cationic Mo oxo NHC complex in this work. B(Ar^F^)_4_ = B(3,5-(CF_3_)_2_C_6_H_3_)_4_.

Since the discovery of olefin metathesis more than half a century ago,^1^ the development of novel catalyst families has been at the core of extensive research efforts, with the goal to increase catalytic performance, from functional group tolerance to increased activities, selectivities and stabilities.^2^ Apart from Ru-based “Grubbs-type” catalysts,^3^ d^0^ early-transition-metal metathesis catalysts mostly based on group 6 metals (Mo/W), *i.e.* Schrock-type catalysts of the general formula M(E)(CHR)(X)(Y) with E = oxo or imido and X, Y = anionic ligands of various types (alkyls, alkoxys, amidos), have emerged as a central class of olefin metathesis catalysts.^4^ Besides, the use of surface organometallic chemistry (SOMC)^5^ has enabled to greatly improve their activity and stability through generation of the corresponding well-defined active sites dispersed at the surface of oxide supports like silica.^6^ In parallel, DFT calculations have revealed that the right balance of the σ-donation ability of the X, Y and E ligands is key to these improved catalytic performances.^7^ The concurrent presence of both strong and weaker σ-donating anionic X and Y ligands for instance avoids overstabilization of the metallacyclobutane intermediates while favouring [2 + 2] cycloaddition and cycloreversion processes as well as suppressing deactivation pathways. N-Heterocyclic carbenes (NHCs) have gained increasing importance as ancillary ligands in transition metal complexes since the 1990s.^8^ In this regard, the introduction of NHCs in overall tetracoordinate, metathesis-active, cationic Mo and W species has illustrated how catalytic performance can be further improved.^9^ Charge delocalization between the NHC and the cationic metal centre renders cationic complexes rather “soft” according to the HSAB principle, which explains the high activity and functional group tolerance in cationic group 6 alkylidene complexes.^10^ The combination of both concepts – introduction of the strong σ-donating NHC ligands and weak siloxy ligands provided by the silica surface – has thus enabled the generation of some of the most active and stable olefin metathesis catalysts to date.^9*b*,*d*,9*g*^ For the cationic tungsten-based catalysts, the oxo-bearing complexes outperformed their imido analogues by 1–2 orders of magnitude in activity;^9*g*^ this can be attributed to the combination of the smaller E ligand size, allowing for easier access of the incoming olefin to the metal centre, and its strong σ-donating ability, which simultaneously enhances catalyst stability. Previously published immobilized neutral Mo oxo alkylidene complexes proved to possess high activity for internal olefins (Fig. 1a).^2*j*,11^ Consequently, we wanted to investigate whether the activity of molybdenum oxo complexes can be boosted for terminal olefins similar to what was shown for analogous tungsten oxo complexes.^9*g*^

## Results and discussion

### Synthesis of molecular catalysts

**Scheme 1:**
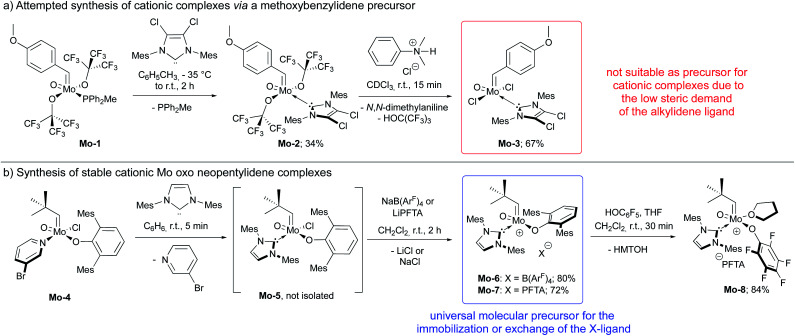
(a) Synthesis of 4-methoxybenzylidene complexes that could not be further transformed into cationic species; (b) synthesis of cationic molybdenum oxo neopentylidene complexes.

**Fig. 2:**
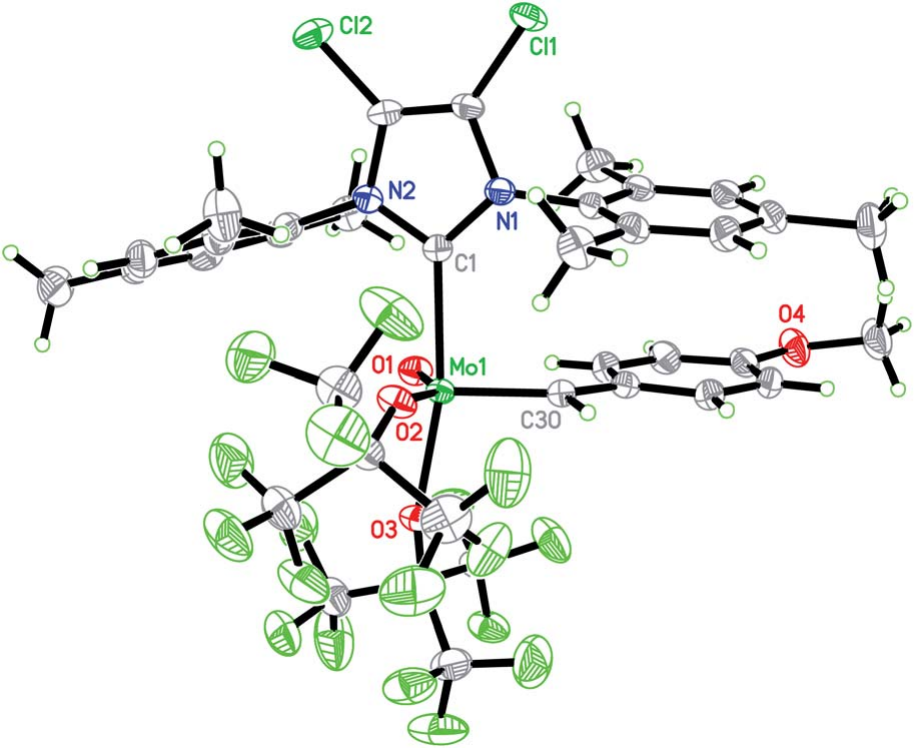
Single crystal X-ray structure of Mo-2. Selected bond lengths [pm] and angles [°]: Mo1–O(1) 166.4(2), Mo1–C(30) 192.1(3), Mo1–O(2) 203.3(2), Mo1–O(3) 206.2(2), Mo1–C(1) 226.3(3), O(1)–Mo1–C(30) 102.28(13), O(1)–Mo1–O(2) 146.29(11), C(30)–Mo1–O(2) 110.88(12), O(1)–Mo1–O(3) 95.40(10), C(30)–Mo1–O(3) 99.27(11), O(2)–Mo1–O(3) 85.36(9), O(1)–Mo1–C(1) 87.88(11), C(30)–Mo1–C(1) 92.56(12), O(2)–Mo1–C(1) 84.81(10), O(3)–Mo1–C(1) 166.73(10).

In this context, we developed and herein report the synthesis and characterization of the long-awaited family of molecular and well-defined silica-supported cationic Mo oxo species stabilized by NHC ligands (Fig. 1b) and investigated their activity towards internal and terminal olefins. The synthesis of cationic molybdenum oxo alkylidene NHC complexes was first attempted starting from the 4-methoxybenzylidene precursor Mo-1 (Scheme 1a).^12^ Addition of 1 equiv. of 1,3-bis(2,4,6-trimethylphenyl)imidazol-2-ylidene (IMes) to Mo-1 resulted in deprotonation of the alkylidene ligand. Through careful choice of a less basic chlorinated NHC,^13^ this side-reaction could be vastly circumvented and the desired product was isolated in 34% yield. Single crystals of Mo-2 for X-ray diffraction were grown from a mixture of dichloroethane/*n*-heptane at −40 °C (Fig. 2). Mo-2 crystallizes in the triclinic space group *P*1̄ with *a* = 1184.11(6) pm, *b* = 1247.18(7) pm, *c* = 1537.13(8) pm, *α* = 80.252(2)°, *β* = 81.208(2)°, *γ* = 66.112(3)°, *Z* = 2. In the solid state, the complex adopts a distorted square pyramidal geometry (*τ*_5_ = 0.34)^14^ with a *syn* alkylidene (^1^*J*_CH_ = 134 Hz) in the apical position. The Mo–oxo bond (166.4 pm) is slightly shorter than in five-coordinate W oxo alkylidene NHC complexes (169.0–176.0 pm) whereas the Mo–NHC bond distance of 226.3 pm is well in the expected range of comparable tungsten complexes.

Exchange of the fluorinated alkoxides was achieved by protonation using *N*,*N*-dimethylanilinium chloride to furnish complex Mo-3. Due to their electron count of 16 (counting the free electron pair at the oxo ligand, too) and their binding site occupied by an NHC, both, Mo-2 and Mo-3 were not expected to exhibit high activity in olefin metathesis. Therefore, we strived for the synthesis of highly active cationic species.^9*k*^

Abstracting one of the X-type ligands using either *N*,*N*-dimethylanilinium B(Ar^F^)_4_ in the reaction with Mo-2 in the presence of pivalonitrile (pivCN) or Ag(pivCN)_3_B(Ar^F^)_4_ in the reaction with Mo-3 resulting in complexes of the formula [MoO(CH-4-(OMe)C_6_H_4_)(X)(IMesCl_2_)(pivCN)][B(Ar^F^)_4_] (X = OC(CF_3_)_3_, Cl) proved to be successful when conducted in CDCl_3_ and monitored by ^1^H NMR. However, the cationic species readily decomposed upon workup, which we attribute to the low steric demand of both, the 4-methoxybenzylidene moiety and the oxo ligand. And indeed, so far, all isolated cationic group 6 alkylidene complexes either bear a neophylidene or neopentylidene ligand^9*c*^ in line with the “almost magic properties” of the neopentylidene ligand contributing to the stability of group 6 complexes.^15^

**Fig. 3:**
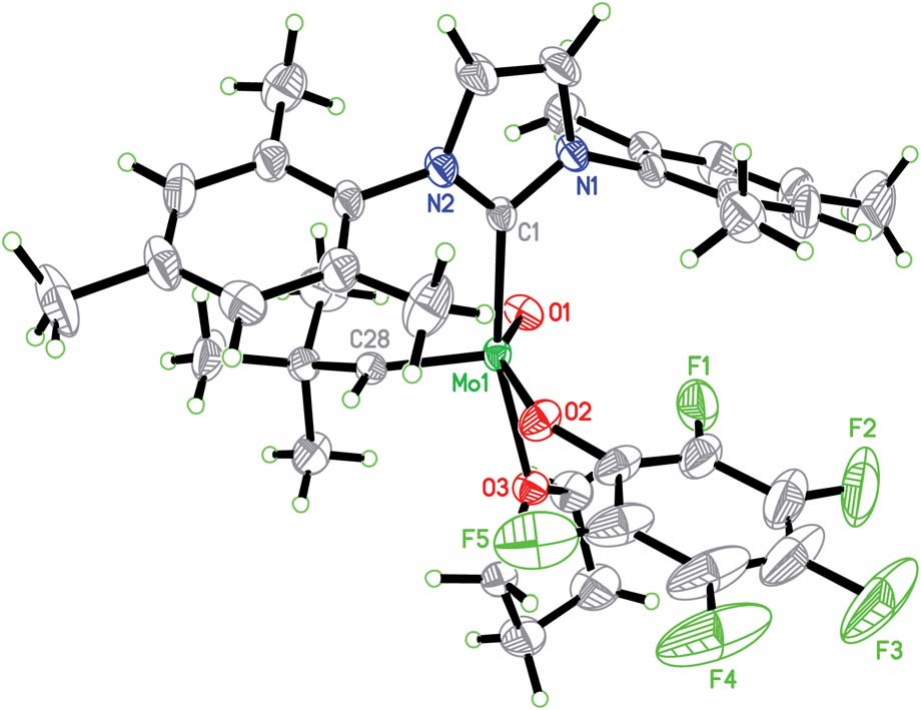
Single crystal X-ray structure of Mo-8, PFTA anion omitted for clarity. Selected bond lengths [pm] and angles [°]: Mo(1)–O(1) 166.4(3), Mo(1)–C(28) 189.1(3), Mo(1)–O(2) 195.8(3), Mo(1)–C(1) 220.3(3), Mo(1)–O(3) 220.5(2), O(1)–Mo(1)–C(28) 104.19(15), O(1)–Mo(1)–O(2) 146.73(13), C(28)–Mo(1)–O(2) 107.41(14), O(1)–Mo(1)–C(1) 94.78(13), C(28)–Mo(1)–C(1) 98.43(13), O(2)–Mo(1)–C(1) 90.70(12), O(1)–Mo(1)–O(3) 85.50(11), C(28)–Mo(1)–O(3) 96.81(12), O(2)–Mo(1)–O(3) 80.74(11), C(1)–Mo(1)–O(3) 164.20(11).

We consequently shifted our attention to a lately published procedure leading to the Mo oxo neopentylidene complex MoO(CHCMe_3_)Cl(OHMT)(3-Brpy) (OHMT = 2,6-dimesitylphenoxide; 3-Brpy = 3-bromopyridine).^16^ 3-Bromopyridine as a relatively weak donor could then be readily replaced by strongly σ-donating IMes (Scheme 1b). In this case, no deprotonation of the alkylidene ligand occurred. The resulting five-coordinate NHC complex was not isolated on account of its poor crystallisation propensity. Nevertheless, the addition of NaB(Ar^F^)_4_ and lithium tetrakis(perfluoro-*t*-butoxy)aluminate (LiPFTA), respectively, to the crude reaction mixture led to formation of the desired cationic complexes that could be purified by recrystallization and which were isolated in good yields of 72 and 80%, respectively, over two steps. The stable cationic species served as efficient and general precursors for immobilization but can also be further transformed by exchange of the X-ligand. Thus, upon addition of an excess of HOC_6_F_5_ to Mo-7, quantitative protonation of the HMTO ligand was facilitated to form Mo-8 in a high isolated yield of 84%. The coordination of THF in complex Mo-8 that lacks the sterically demanding alkoxide is imperative for its purification by recrystallization. Single crystals of Mo-8 suitable for X-ray diffraction were grown from 1,2-dichloroethane at −40 °C (Fig. 3). As for Mo-2, the triclinic space group *P*1̄ was found in Mo-8 with the unit cell dimensions being *a* = 1296.54(8) pm, *b* = 1388.76(9) pm, *c* = 1930.09(12) pm, *α* = 79.898(2)°, *β* = 78.994(2)°, *γ* = 86.057(3)° and two molecules in the unit cell. The distorted square pyramidal structure (*τ*_5_ = 0.29) bears a *syn*-alkylidene ligand in the apical position and all other ligands in the equatorial plane. All bond lengths and angles are similar to those observed for five-coordinate cationic W oxo alkylidene NHC complexes.^17^

On a final note regarding the synthesis of cationic Mo oxo alkylidene complexes, the synthesis of further species bearing fluorinated alkoxides starting from aryloxide complexes Mo-6 and Mo-7 have been attempted using HOC(CF_3_)_3_. Unfortunately, the reactions proved unsuccessful, and no conversion was observed. However, the aryloxide ligand in Mo-6 and Mo-7 was successfully protonated by the action of HCl in Et_2_O in the presence of pivCN to yield a mixture of complexes of the type [MoO(CHCMe_3_)Cl(IMes)(pivCN)][X] (X = B(Ar^F^)_4_ or LiPFTA) and the protonated OHMT ligand as an inseparable oil. All attempts to isolate the complexes by crystallization or to further convert them *in situ* using MOC(CF_3_)_3_ (M = Li, Ag) failed.

### Immobilization of complexes

**Scheme 2:**
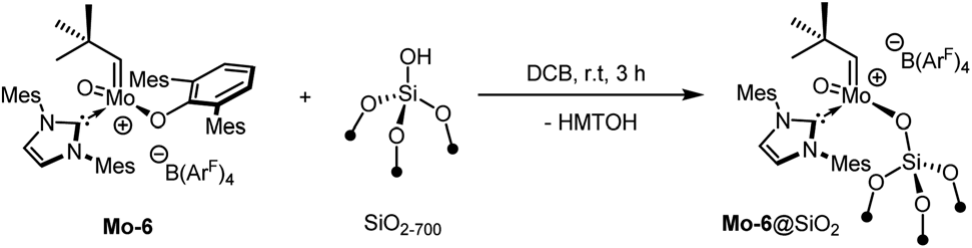
Grafting Mo-6 onto SiO_2–700_.

The reaction of Mo-7 with HOC_6_F_5_ illustrates that protic functional groups can lead to the very selective exchange of the X-ligand without notable side-reactions. Following the development of the molecular compounds, we also sought to exploit this reactivity for grafting the complex on silica partially dehydroxylated at 700 °C (SiO_2–700_). For comparability with previously published complexes,^9*b*,*d*,9*g*^ the B(Ar^F^)_4_-containing complex Mo-6 was selected for this purpose and yielded the corresponding supported species Mo-6@SiO_2_ (Scheme 2).

Quantification by ^19^F solution NMR spectroscopy of the remaining B(Ar^F^)_4_ of the complex indicates that *ca.* 29% of the surface silanols (0.32 mmol g^−1^) reacted with Mo-6, in line with a molybdenum loading of 0.79 wt% as determined by elemental analysis. IR spectroscopy confirms that a significant amount of isolated silanols (*ν̃* = 3747 cm^−1^) are consumed upon grafting by protonolysis, although the large size of Mo-6 likely prevents full coverage.

Besides of the appearance of CH stretching bands between *ν̃* = 3200 and 2800 cm^−1^, a broad absorption band appeared at around *ν̃* = 3700 cm^−1^, typical of the non-covalent interactions of unreacted surface silanols with the aromatic ligands of the grafted complex.^2*h*,9*b*,9*d*,11^ Further characterization of Mo-6@SiO_2_ by ^1^H magic angle spinning (MAS) NMR showed the alkylidene proton resonance at 13.6 ppm, slightly upfield with respect to the molecular precursor signals in solution (13.9 ppm). The ^13^C cross-polarization (CP) MAS NMR spectrum shows the presence of the different methyl moieties and aromatics, whereas the alkylidene carbon, unfortunately, was not observed (for further details see ESI Fig. S1 and S2†).

### Catalytic testing of molecular and immobilized complexes

Evaluation of the catalytic activity for Mo-6 was carried out for both molecular and immobilized complexes in the homo metathesis of the two benchmark substrates *cis*-4-nonene and 1-nonene at 30 °C (Table 1). While the homogeneous complex Mo-6 had to be tested in *o*-dichlorobenzene (DCB) due to solubility requirements, the immobilized analogue Mo-6@SiO_2_ was tested in both DCB and toluene, prototypical metathesis solvents used in many studies. In all cases, the metathesis activity towards α-olefins was higher than for internal ones. This behaviour was most pronounced for the molecular complex Mo-6, where no activity was observed for internal olefins and only 86% conversion after 8 hours was reached with 1-nonene. The lack of reactivity is likely associated with the presence of the large phenoxy (OHMT) ligand, whose steric encumbrance probably hinders the required distortion of the metal complex to a trigonal prism necessary to bind the incoming olefin.^2*j*^

**Table 1:** Homocoupling of *cis*-4-nonene and 1-nonene (toluene, 30 °C)

Catalyst	Mol%	TOF_3 min_^a^	Time to equilibrium/maximum conversion^b^
**Metathesis of *cis*-4-nonene** c			
Mo-6^e^	0.1	— (<1%)	5% after 24 h
Mo-6@SiO_2_^e^	0.1	18 (6%)	50% after 16 h
Mo-6@SiO_2_	0.1	26 (8%)	50% after 4 h

**Metathesis of 1-nonene** d			
Mo-6^e^	0.1	47 (14%/98%)	86% after 8 h
Mo-6@SiO_2_^e^	0.1	124 (37%/97%)	98% after 4 h
Mo-6@SiO_2_	0.1	118 (36%/98%)	98% after 4 h
Mo-6@SiO_2_	0.02	254 (15%/98%)	97% after 8 h
Mo-6@SiO_2_	0.005	225 (7%/98%)	94% after 8 h

a TOF at 3 min, given in min^−1^.

b In cases where full conversion was not reached, the maximum conversion measured after a given time is provided.

c Equilibrium conversion is 50%, conversion in brackets.

d Maximum conversion is 100%, conversions/selectivity for the formation of hexadec-8-ene in brackets.

e Experiment performed in *o*-dichlorobenzene.

**Fig. 4:**
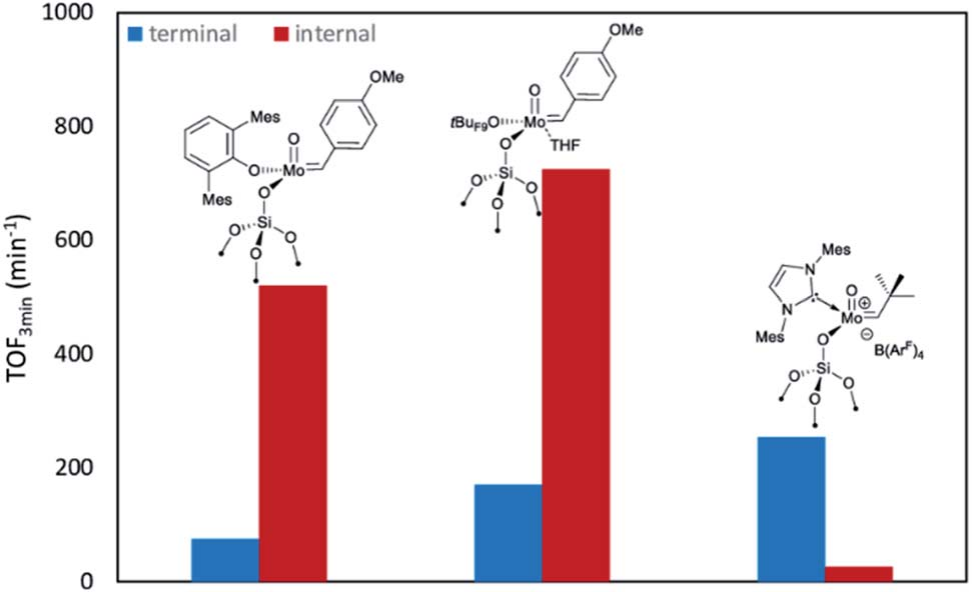
Overview of TOF_3 min_ for the homo metathesis of *cis*-4-nonene (internal) and 1-nonene (terminal) of previously reported neutral Mo oxo alkylidene species^2*j*,11^ compared to the novel cationic complex Mo-6@SiO_2_; all reactions in toluene, at 30 °C and at optimal catalyst loadings at which the highest TOF values have been reported (Mo-6@SiO_2_: 0.1 mol% for *cis*-4-nonene and 0.02 mol% for 1-nonene; MoO(CH-4-(OMe)C_6_H_4_)(OHMT)@SiO_2_: 0.005 mol% for *cis*-4-nonene and 0.1 mol% for 1-nonene; MoO(CH-4-(OMe)C_6_H_4_)(*t*Bu_F9_)(THF)@SiO_2_: 0.02 mol% for *cis*-4-nonene and 0.02 mol% for 1-nonene).

In fact, switching OHMT for the smaller surface siloxy ligand leads to an increase in activity towards both olefins.^2*g*,*h*^ Notably, the initial turnover frequency recorded for the self-metathesis of *cis*-4-nonene was modest in comparison to other molybdenum oxo alkylidenes (TOF_3 min_ = 26 min^−1^*vs.* >500 min^−1^).^2*j*,11^ This observation is in line with the reported activity difference for the cationic tungsten analogues^9*b*^ and established trends that show increased activity for internal olefins in Schrock-type metathesis catalysts with decreasing σ-donor strength of the X ligands (X = NHC, pyrrolide, alkoxide).^9*b*,11,18^ Considering also the activity for terminal olefins, the same correlation can only be observed in few cases.^11^ The decisive factor for a high catalytic activity might be rather the right balance of strong and weak σ-donor ligands as outlined above.^7^ However, the activity of Mo-6@SiO_2_ towards terminal olefins exceeds those previously reported for this family of supported complexes with TOF_3 min_ = 254 min^−1^ at 0.02 mol% catalyst loading compared to only 75 min^−1^ and 170 min^−1^ of their neutral congeners at their optimal loadings (Fig. 4).^2*j*,11^ Indeed, this supports our working hypothesis that the catalytic activity of cationic Mo oxo alkylidenes is enhanced for terminal olefins similarly as shown previously for the corresponding tungsten oxo complexes.^9*g*^ Furthermore, the catalyst remained active (94% after 8 h), when increasing the substrate to catalyst ratio to 10 000 : 1.

The excellent performance of Mo-6@SiO_2_ was additionally demonstrated by recycling tests, where maximum conversion was obtained within 8 h three times, albeit accompanied by a constant loss of activity (see ESI†).

In previous studies it was shown that the homometathesis of 1-nonene using neutral immobilized Mo alkylidene catalysts can lead to low selectivities through isomerization of the position of the double bond. This can result in a selectivity of the desired hexadec-8-ene of only 80–85%, especially in closed vessels and at prolonged reaction times.^2*m*,5*b*^ This type of isomerization can be attributed to Mo(iv) species resulting from methylidene complex decomposition that form π-complexes with olefins in the reaction mixture. Subsequently, the π-complexes undergo oxidative addition and reductive elimination processes and, thereby, lead to isomerization.^19^ Monitoring the selectivity for the formation of the homocoupling product in the metathesis of 1-nonene using cationic Mo oxo alkylidene complexes, in contrast, demonstrated that neither the homogeneous nor the immobilized catalyst are prone to form structural isomers. Even after long reaction times of 8 h, high selectivities of *ca.* 98% were obtained.

## Conclusions

In conclusion, catalysts belonging to the new family of cationic Mo oxo alkylidenes NHC complexes have been developed. The immobilization on silica yielded a catalyst with preferential activity towards α-olefins over internal olefins. This particular reactivity is attributed to the nature of the NHC ligand, whose steric demand and strong σ-donor ability have an important influence on the formation and reversion of the TBP metallacyclobutane. These results further contribute to the overall understanding on how structural and electronic properties of metal alkylidenes affect the catalytic performance and further highlight the role of the NHC ligand in providing highly active cationic d^0^-based olefin metathesis catalysts.

## Experimental

### General considerations

All experiments were carried out under an inert nitrogen or argon atmosphere using Schlenk techniques or an MBraun or GS glovebox equipped with a purifier unit. Water and oxygen levels were kept below 0.1 ppm. Diethyl ether, methylene chloride, pentane, toluene and tetrahydrofuran were purified using double MBraun SPS alumina columns. Benzene and benzene-d_6_ were distilled from Na/benzophenone. 1,2-Dichlorobenzene was distilled from CaH_2_. All solvents were degassed by three consecutive freeze–pump–thaw cycles.

Elemental analyses were performed at Mikroanalytisches Labor Pascher, Germany, and at the Institute of Inorganic Chemistry, University of Stuttgart, Germany. All infrared (IR) spectra were recorded using a Bruker FT-IR Alpha spectrometer placed inside a glovebox, equipped with the OPUS software. The IR spectrometer had a total spectral range of 275–7500 cm^−1^ with a resolution <2 cm^−1^ and consisted of a RockSolid interferometer, a DTGS (triglycine sulfate) detector and a SiC globar source. Solid samples were investigated in a magnetic pellet holder. A typical experiment consisted of 32 consecutive transmission measurements in the region from 4000 to 400 cm^−1^. Solution NMR spectra were recorded on a Bruker Avance III 400. Chemical shifts are reported in ppm relative to the solvent signal (CDCl_3_: 7.26 ppm, C_6_D_6_ 7.16 ppm, CD_2_Cl_2_ 5.13 ppm).^20^ Data are reported as follows: chemical shift, multiplicity (s = singlet, d = doublet, t = triplet, q = quartet, quint = quintet, sept = septet, br = broad, m = multiplet), coupling constants (Hz) and integration. The ^1^H and ^13^C solid-state NMR spectra were acquired on a Bruker AVANCE III spectrometer operating at 400 MHz ^1^H frequency (9.4 T) and equipped with a 3.2 mm proton-heteronucleus magic angle spinning (^1^H,X MAS) probe (Bruker). Samples were packed into a 3.2 mm MAS NMR zirconia rotor closed with a VESPEL drive cap in an argon-filled glovebox, transferred to the NMR spectrometer in a tightly sealed vial under argon, rapidly inserted into the NMR spectrometer and spun under dry nitrogen. The MAS frequency was set to 16 kHz for all experiments. For the ^13^C CP MAS measurements, the Hartman–Hahn cross-polarization was performed by making use of a linear ramp from 70% to 100% with the contact time set to 3 ms. For decoupling, SPINAL64 was applied with 100 kHz irradiation on ^1^H. The acquired solid-state NMR spectra are referenced externally with respect to the downfield signal of adamantane (38.5 ppm). All MAS NMR spectra were acquired at ambient temperature.

Liquid catalytic test aliquots were analysed using a GC/FID (Agilent Technologies 7890 A) equipped with a split–splitless injector heated to 250 °C, injection volume 0.5 μL using hydrogen carrier gas. Chromatographic separations for 1-nonene and *cis*-4-nonene catalytic tests were performed using an HP-5 (Agilent Technologies) column (30 m, 0.32 mm, 0.25 μm stationary phase). Crystal data have been deposited with the Cambridge Crystallographic Data Centre (CCDC): Mo-2 CCDC 2151036, Mo-8 CCDC 2151037.

#### Preparation of precursor reagents

The following reagents were prepared according to literature procedures: IMes,^21^ IMesCl_2_,^22^ NaB(Ar^F^)_4_,^23^ Li[Al(OC_4_F_9_)_4_],^24^ [MoO(CH-4-OMeC_6_H_4_)(OC(CF_3_)_3_)_2_(PPh_2_Me)],^12^ [MoO(CHCMe_3_)(OHMT)(Cl)(3-BrPy)].^25^

#### Substrates and internal standard

1-Nonene (TCI ≥ 99.5%), *cis*-4-nonene (TCI ≥ 95%) and decaline (Sigma Aldrich 98%) were distilled from Na, degassed by three consecutive freeze–pump–thaw cycles, filtered through activated alumina, and stored for 4 hours over activated Selexsorb CD® and activated 4 Å molecular sieves.

#### Silica

Silica (Aerosil Degussa, 200 m^2^ g^−1^) was compacted with distilled water, sieved, calcined at 500 °C under air for 12 h and treated under vacuum (10^−5^ mbar) at 500 °C for 8 h and then at 700 °C for 14 h (referred to as SiO_2–700_).

### Synthesis of molecular precursors

#### [MoO(CH-4-OMeC_6_H_4_)(OC(CF_3_)_3_)_2_(IMesCl_2_)] (Mo-2)

A cold (−35 °C) solution of IMesCl_2_ (112 mg, 0.30 mmol, 0.9 equiv.) in toluene (4 mL) was added dropwise to a cold (−35 °C) solution of [MoO(CH-4-OMeC_6_H_4_)(OC(CF_3_)_3_)_2_(PPh_2_Me)] (Mo-1, 300 mg, 0.33 mmol, 1 equiv.) in toluene (4 mL). The reaction mixture was stirred at room temperature for 30 minutes and then stored at −35 °C for three hours. The solvent was removed under reduced pressure and the residue was triturated with *n*-pentane to give a brown solid. The *n*-pentane was decanted and diethyl ether (4 mL) was added, which resulted in the formation of a dark brown solution and an orange solid. The solution was decanted and the solid was washed with diethyl ether (1 mL) and dried *in vacuo*. Yield: 120 mg (34%). Crystals suitable for single-crystal X-ray diffraction were obtained from a mixture of 1,2-dichloroethane and *n*-heptane. ^1^H NMR (400 MHz, CDCl_3_) *δ* 13.50 (s, 1H), 6.99 (s, 2H), 6.93–6.13 (m, 6H), 3.87 (s, 3H), 2.24 (s, 6H), 2.13 (s, 6H), 1.92 (s, 6H) ppm. ^19^F NMR (376 MHz, CDCl_3_) *δ* −72.64 ppm. ^13^C NMR (101 MHz, CDCl_3_) *δ* 294.5, 183.9, 163.2, 141.2, 136.4, 135.9, 133.7, 131.5, 129.5, 129.3, 125.9, 123.0, 120.1, 117.2, 112.3 (br), 83.6 (br), 55.5, 21.2, 18.2, 17.9 ppm. Elemental analysis (%) calcd. for C_37_H_30_Cl_2_F_18_MoN_2_O_4_: C 41.32, H 2.81, N 2.60; found: C 41.48, H 3.00, N 2.60.

#### [MoO(CH-4-OMeC_6_H_4_)Cl_2_(IMesCl_2_)] (Mo-3)

A solution of *N*,*N*-dimethylanilinium chloride (14.7 mg, 0.09 mmol, 2 equiv.) in CDCl_3_ (0.7 mL) was added to a solution of [MoO(CH-4-OMeC_6_H_4_)(OC(CF_3_)_3_)_2_(IMesCl_2_)] (Mo-2, 50.0 mg, 0.05 mmol, 1 equiv.) in CDCl_3_ (0.7 mL). The resulting red solution was stirred at room temperature for 15 minutes after which full conversion was confirmed by ^1^H NMR. The solvent was removed under reduced pressure and the residue was triturated with *n*-pentane to yield a red solid. The *n*-pentane was decanted and the solid was washed with diethyl ether. The solvent was decanted again and the solid was dried *in vacuo*. Yield: 21 mg (67%). ^1^H NMR (400 MHz, CDCl_3_) *δ* 12.17 (s, 1H), 7.42–7.20 (m, 2H), 7.03 (s, 2H), 6.88 (d, *J* = 8.6 Hz, 2H), 6.82 (s, 2H), 3.89 (s, 3H), 2.37 (s, 6H), 2.13 (s, 6H), 1.88 (s, 6H) ppm. ^13^C NMR (101 MHz, CDCl_3_) *δ* 303.1, 186.4, 163.9, 141.0, 136.6, 136.0, 133.5, 132.1, 131.0, 130.1, 129.7, 120.0, 113.2, 55.7,34.1, 21.3, 18.3, 18.3 ppm. Elemental analysis (%) calcd. for C_29_H_30_Cl_4_MoN_2_O_2_: C 51.50, H 4.47, N 4.14; found: C 51.30, H 4.55, N 4.12.

#### [MoO(CHCMe_3_)(OHMT)(IMes)][B(Ar^F^)_4_] (Mo-6)

A solution of IMes (66.9 mg, 0.21 mmol, 1 equiv.) in benzene (2 mL) was added to a solution of [MoO(CHCMe_3_)(OHMT)(Cl)(3-BrPy)] (155.0 mg, 0.21 mmol) in benzene (2 mL). The reaction was stirred at room temperature for 5 minutes and the solvent was removed *in vacuo*. The residue was co-evaporated with diethyl ether (2 × 1 mL) *n*-pentane (2 × 1 mL) to yield a yellow solid. The solid was dissolved in CH_2_Cl_2_ (3 mL) and the resulting solution was added to a suspension of NaB(Ar^F^)_4_ (194.8 mg, 0.21 mmol, 1 equiv.) in CH_2_Cl_2_ (2 mL). After stirring for two hours at room temperature, the solvent was removed under reduced pressure. The residue was stirred with *n*-pentane (10 mL), which resulted in the formation of a yellow suspension. The solvent was decanted and the solid was washed with *n*-pentane (4 mL) one more time. The solid was dried *in vacuo*, dissolved in CH_2_Cl_2_ (2 mL) and the resulting suspension was filtered through a pad of Celite and *n*-pentane was added to the filtrate. Upon storage at −35 °C yellow crystals formed. Yield: 296.0 mg (80%). ^1^H NMR (400 MHz, CD_2_Cl_2_) *δ* 13.96 (s, 1H), 7.75 (s, 8H), 7.57 (s, 4H), 7.26 (s, 2H), 7.25–7.17 (m, 1H), 7.01 (s, 2H), 6.99 (s, 2H), 6.97 (d, 2H), 6.94 (s, 2H), 6.83 (s, 2H), 2.42 (s, 6H), 2.33 (s, 6H), 1.90 (s, 6H), 1.87 (s, 6H), 1.85 (s, 6H), 1.55 (s, 6H), 0.89 (s, 9H) ppm. ^19^F NMR (376 MHz, CD_2_Cl_2_) *δ* −62.82 ppm. ^13^C NMR (101 MHz, CD_2_Cl_2_) *δ* 342.2, 173.7, 162.4 (q, ^1^*J*_CB_ = 49.8 Hz), 159.1, 142.2, 139.5, 137.6, 136.3, 136.0, 135.4, 134.0, 134.0, 131.2, 131.2, 130.6, 130.5, 129.9, 129.5 (qq, ^2^*J*_CF_ = 31.6, ^3^*J*_CB_ 3.0 Hz), 127.3, 125.7, 125.2 (q, ^1^*J*_CF_ = 272.5 Hz), 118.1, 49.9, 30.3, 30.3, 21.4, 21.0, 18.3, 17.2 ppm. Elemental analysis (%) calcd. for C_82_H_71_BF_24_MoN_2_O_2_: C 58.65, H 4.26, N 1.67; found: C 58.48, H 4.27, N 1.77.

#### [MoO(CHCMe_3_)(OHMT)(IMes)][Al(OC_4_F_9_)_4_] (Mo-7)

A solution of IMes (66.9 mg, 0.21 mmol, 1 equiv.) in benzene (2 mL) was added to a solution of [MoO(CHCMe_3_)(OHMT)(Cl)(3-BrPy)] (155.0 mg, 0.21 mmol) in benzene (2 mL). The reaction was stirred at room temperature for 5 minutes and the solvent was removed *in vacuo*. The residue was co-evaporated with diethyl ether (2 × 1 mL) and *n*-pentane (2 × 1 mL) and subsequently dissolved in CH_2_Cl_2_ (3 mL). This solution was added to a suspension of Li[Al(OC_4_F_9_)_4_] (214.2 mg, 0.21 mmol, 1 equiv.) in CH_2_Cl_2_ (2 mL). After stirring the reaction at room temperature for 2 h, the solvent was removed under reduced pressure. The residue was triturated with pentane, the solvent was decanted and the solid dried *in vacuo*. The solid was dissolved in CH_2_Cl_2_ and filtered through a pad of Celite. Diethyl ether and *n*-pentane were added to the filtrate. Upon storage at −35 °C orange crystals formed. Yield: 281 mg (72%). ^1^H NMR (400 MHz, CD_2_Cl_2_) *δ* 13.96 (s, 1H), 7.26 (s, 2H), 7.23–7.17 (m, 1H), 7.03 (s, 2H), 7.00 (s, 2H), 6.98 (d, *J* = 7.5 Hz, 2H), 6.94 (s, 2H), 6.82 (s, 2H), 2.44 (s, 6H), 2.34 (s, 6H), 1.90 (s, 6H), 1.87 (s, 6H), 1.85 (s, 6H), 1.56 (s, 6H), 0.89 (s, 9H) ppm. ^19^F NMR (376 MHz, CD_2_Cl_2_) *δ* −75.71 ppm. ^13^C NMR (101 MHz, CD_2_Cl_2_) *δ* 342.1, 173.7, 159.2, 142.3, 139.5, 137.6, 136.3, 136.0, 134.9, 134.1, 134.0, 131.3, 131.2, 130.6, 129.8, 127.3, 125.7, 121.9 (q, ^1^*J*_CF_ = 291.7 Hz), 79.5 (br), 50.0, 30.3, 21.4, 21.0, 18.2, 17.1 ppm. Elemental analysis (%) calcd. for C_66_H_59_AlF_36_MoN_2_O_6_: C 44.46, H 3.34, N 1.57; found: C 44.29, H 3.51, N 1.59.

#### [MoO(CHCMe_3_)(OC_6_F_5_)(IMes)(THF)][Al(OC_4_F_9_)_4_] (Mo-8)

To a solution of [MoO(CHCMe_3_)(OHMT)(IMes)][Al(OC_4_F_9_)_4_] (150 mg, 84.1 μmol, 1 equiv.) in CH_2_Cl_2_ (2 mL) was added a solution of C_6_F_5_OH (31.0 mg, 168 μmol, 2 equiv.) in CH_2_Cl_2_ (1 mL), then five drops of THF were added. The reaction was stirred at room temperature for 1 h and the solvent was removed *in vacuo*. The residue was washed with pentane (3 × 1 mL) to remove excess of C_6_F_5_OH and HOHMT. The residue was recrystallized from CH_2_Cl_2_, diethyl ether and *n*-pentane to yield the product as yellow crystals in a yield of *o* 121 mg (84%). ^1^H NMR (400 MHz, CD_2_Cl_2_) *δ* 13.97 (s, 1H), 7.33 (s, 2H), 7.06 (d, *J* = 15.0 Hz, 4H), 3.68–3.48 (m, 4H), 2.39 (s, 6H), 2.04 (s, 6H), 1.92 (s, 6H), 1.73 (s, 4H), 1.15 (s, 9H). ^19^F NMR (376 MHz, CD_2_Cl_2_) *δ* −75.75 (s, 36F), −160.88 to −161.18 (m, 2F), −163.32 to −163.57 (m, 2F), −166.28 to −166.50 (m, 1F). ^13^C NMR (101 MHz, CD_2_Cl_2_) *δ* 340.6, 178.3, 141.9, 140.5, 140.4, 140.1, 139.9, 138.2, 138.0, 137.6, 137.4, 136.7, 136.6, 135.8, 134.8, 134.5, 130.4, 130.4, 126.5, 126.5, 121.8 (q, ^1^*J*_CF_ = 292.9 Hz), 79.5 (br), 75.6, 50.0, 30.6, 25.8, 21.4, 21.3, 18.2, 18.0, 18.0. Elemental analysis (%) calcd. for C_52_H_42_AlF_41_MoN_2_O_7_: C 36.55, H 2.48, N 1.64; found: C 36.43, H 2.70, N 1.47. (Due to broad and overlapping signals ^1^*J*_CF_ in OC_6_F_5_ could not be determined).

### Synthesis of silica-supported complex Mo-6@SiO_2_

#### [(≡SiO)Mo(O)(CHCMe_3_)(IMes)][B(Ar^F^)_4_], Mo-6@SiO_2_

A yellow solution of Mo-6 (78.8 mg, 0.047 mmol, 1 equiv.) was added to a suspension of SiO_2–700_ (160.1 mg, 0.051 mmol SiOH, 1.1 equiv.) in *o*-dichlorobenzene (5 mL) at 25 °C in a nitrogen filled glovebox. The suspension was slowly stirred (120 rpm) over 3 h. The supernatant was removed by decantation and the solid was washed by suspension/decantation cycles in *o*-dichlorobenzene (3 × 1 mL), toluene (3 × 1 mL) and pentane (3 × 2 mL). The resulting light-yellow solid was dried thoroughly under high vacuum (10^−5^ mbar) at room temperature for 2 hours to afford 180 mg of the title compound. All filtrate solutions were collected and analysed by ^19^F NMR spectroscopy in C_6_D_6_ using 4,4′-difluorobiphenyl as internal standard, indicating that 0.015 mmol of the molecular complex remained in solution, corresponding to 29% grafting. Mo 0.79%, C 5.85%, H 0.53%, N 0.47%, corresponding to 59 C/Mo (82 expected), 64 H/Mo (71 expected), 4 N/Mo (2 expected).

## Data availability

Primary and complementary data are available from https://doi.org/10.18419/darus-3013.

## Author contributions

JVM and JDJS contributed equally.

## Conflicts of interest

There are no conflicts to declare.

## Supplementary Material

SC-013-D2SC03321F-s001

SC-013-D2SC03321F-s002
