# Supplementary material for: Cationic molybdenum oxo alkylidenes stabilized by N-heterocyclic carbenes: from molecular systems to efficient supported metathesis catalysts
Source: Chem Sci. 2022 Jul 12;13(29):8649–56. doi: 10.1039/d2sc03321f (PMC9337747; doi:10.1039/d2sc03321f)
Supplement: SC-013-D2SC03321F-s001 [file SC-013-D2SC03321F-s001.pdf]

Electronic Supplementary Material (ESI) for Chemical Science.
This journal is © The Royal Society of Chemistry 2022

Cationic Molybdenum Oxo Alkylidenes Stabilized by *N*-Heterocyclic Carbenes: From Molecular Systems to Efficient Supported Metathesis Catalysts

Janis V. Musso,[a]‡ Jordan De Jesus Silva,[b]‡ Mathis J. Benedikter,[a] Jonas Groos,[a] Wolfgang Frey,[c] Christophe Copéret,[b]* Michael R. Buchmeiser[a,d]*

[a] M.Sc. J. V. Musso, Dr. M. J. Benedikter, M.Sc. Jonas Groos, Prof. Dr. M. R. Buchmeiser
Institute of Polymer Chemistry, Universität Stuttgart
Pfaffenwaldring 55, 70569 Stuttgart, Germany
E-mail: michael.buchmeiser@ipoc.uni-stuttgart.de

[b] M.Sc. J.D.J. Silva, Prof. Dr. C. Copéret
Department of Chemistry and Applied Biosciences, ETH Zürich,
Vladimir-Prelog-Weg 1–5, CH-8093 Zürich, Switzerland
E-mail: ccoperet@ethz.ch

[c] Dr. W. Frey
Institut of Organic Chemistry, Universität Stuttgart
Pfaffenwaldring 55, 70569 Stuttgart, Germany

[d] German Institutes of Textile and Fiber Research (DITF) Denkendorf
Körschtalstr. 26, 73770 Denkendorf, Germany

* Corresponding authors

‡ These authors contributed equally

Table of Contents

1. Characterization of the Silica-Supported Complex 3

2. Experimental Procedures and Catalytic Data 4

Plots of Conversion vs Time and Selectivity vs Time for *Cis*-4-Nonene Experiments 7

Plots of Conversion vs Time and Selectivity vs Time for 1-Nonene Experiments 13

Recycling Tests with 1-Nonene 23

3. Solution NMR Spectra 25

4. Single Crystal X-Ray Crystallography Data 32

1. Characterization of the Silica-Supported Complex

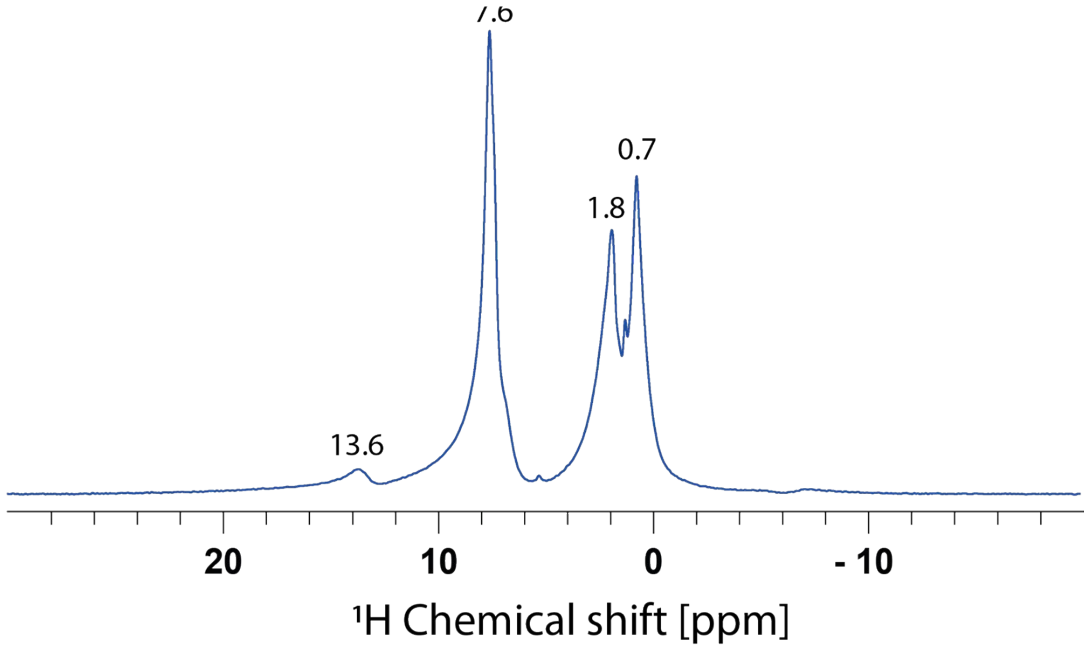

Figure S1. Top: ^{1}H MAS NMR (400 MHz, spinning rate 16 kHz, recycle delay 2 s) spectrum of **Mo-6**@SiO_2 (* spinning side bands); proton resonance at 13.6 ppm, *tert*-butyl proton resonances at 0.7 ppm, the methyl moieties of the mesityl groups at 1.8 ppm and the aromatic protons at 7.6 ppm, respectively.

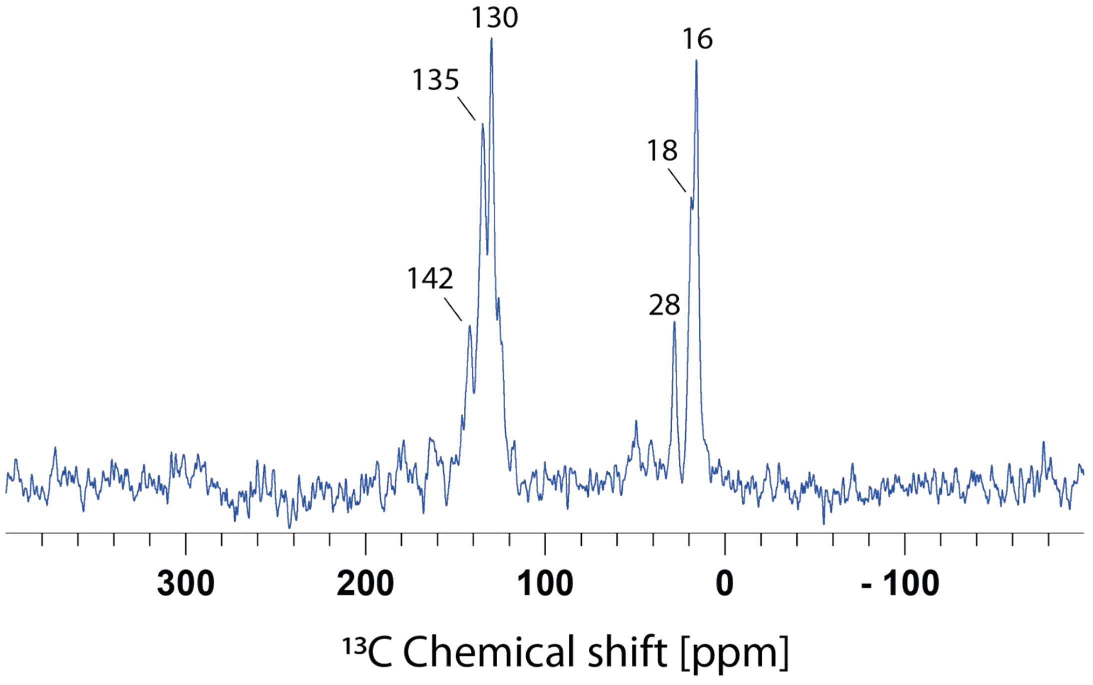

Figure S2. ^{13}C CP MAS NMR spectrum (400 MHz, spinning rate 16 kHz, contact time 3 s) of **Mo-6**@SiO_2, different methyl moieties at 16, 18 and 28 ppm, as well as the presence of

aromatics at 130, 135 and 142 ppm.

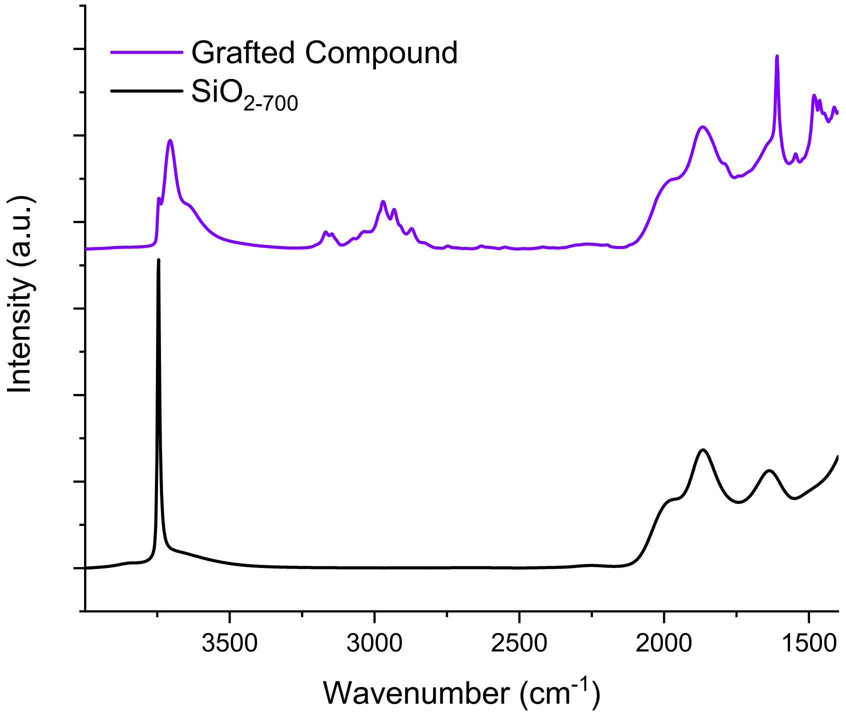

Figure S3. Transmission FT-IR spectra (from top) of **Mo-6**@SiO_2 purple, and $SiO_{2\text{-}(700)}$ blue.

2. Experimental Procedures and Catalytic Data

Metathesis of *cis*-4-nonene:

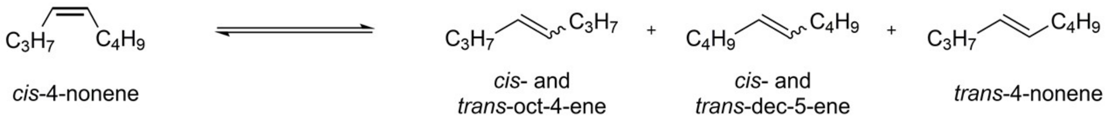

Every dispensing step for experiments with *cis*-4-nonene was performed manually inside a nitrogen-filled glovebox (<0.1 ppm H_2O and O_2). At t=0, a 0.95 M solution of *cis*-4-nonene in toluene or o-dichlorobenzene containing decaline as internal standard (0.1 M) was added to the catalyst placed inside a 10 mL screw cap vial containing a magnetic stirrer, and the reaction was stirred at 620 rpm. The reaction mixtures were agitated for 1 hour and 10 µL aliquots were taken after ca. 3, 5, 10, 30 and 60 min after the addition of *cis*-4-nonene. The aliquots were dispensed into GC vials containing 500 µL of a solution of 4-heptanone in toluene (ca $5.6 \cdot 10^{-4}$ M) for quenching. The resulting solution was analyzed by GC/FID (Agilent Technologies 7890 A) equipped with an HP-5 (Agilent Technologies) column. Quantification gave conversions and (*E*)/(*Z*) selectivities for oct-4-ene and dec-5-ene. Equilibrium conversion was reached at ca. 50%.

Metathesis of 1-nonene:

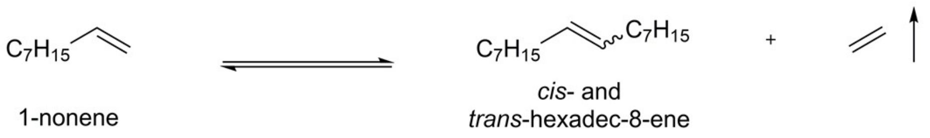

Every agitation and dispensing step for experiments with 1-nonene were performed by the liquid handling robot (Zinsser Analytics) operated inside a nitrogen-filled glovebox (<0.1 ppm H_2O and O_2). At t=0, a 0.95 M solution of 1-nonene in toluene or *o*-dichlorobenzene containing decaline as internal standard (0.1 M) was added to the catalyst introduced in a 10 mL vial and the reaction mixture was agitated at 300 rpm. Catalytic tests were conducted open to the glove-box atmosphere. The reaction mixtures were agitated for 8 hours and 30 µL aliquots were taken after ca. 3, 10, 30, 60, 120, 240 and 480 min after the addition of 1-nonene. The aliquots were dispensed into GC vials containing 500 µL of a solution of 4-heptanone in toluene (ca $5.6 \cdot 10^{-4}$ M) for quenching. The resulting solution was analyzed by GC/FID (Agilent Technologies 7890 A) equipped with an HP-5 (Agilent Technologies) column. Quantification gave conversions and (*E*)/(*Z*) selectivities for hexadec-8-ene. Maximum conversion is reached at ca. 100% in our conditions.

$$X(\text{substrate}) = \frac{n(substrate)initial - n(substrate)}{n(substrate)initial} \times 100\% \quad (1)$$

$$\text{TOF}_{3\text{min}} = \frac{X(substrate)\ at\ 3min}{100 \times 3\ min} \times \frac{n(substrate)}{n(catalyst)}, \text{ in [min}^{-1}] \quad (2)$$

$$S_{C16}(E/Z) = \frac{n(C16E)\ /\ n(C16)}{(\ n(C16) - (n(C16E))\ /\ n(C16))} \quad (3)$$

Table S1. Homometathesis of *cis*-4-nonene **(**toluene, 30 °C).

Catalyst[a]	*mol%*	*TOF*$_{3min}$*[a]*	*Time to equilibrium conversion* *[b][c]*
**Mo-6**[d]	0.1	– (<1%)	5% after 24 h
**Mo-6**@SiO_2[d]	0.1	18 (6%)	50% after 16 h
**Mo-6**@SiO_2	0.1	26 (8%)	50% after 4 h

[a] TOF at 3 min, given in min^{-1} with the corresponding conversions given in brackets. [b] In cases where full

conversion was not reached, the maximum conversion measured after a given time is provided. [c] Equilibrium conversion is 50%. [d] Experiment performed in *o*-dichlorobenzene.

Table S2. Homometathesis of 1-nonene (toluene, 30 °C).

Catalyst [a]	***mol%***	***TOF_{3min}*** [a]	***Time to maximum conversion*** [b][c]
**Mo-6** [d]	0.1	47 (14%)	86% after 8 h
**Mo-6**@SiO_2 [e]	0.1	124 (37%)	98% after 4 h
**Mo-6**@SiO_2	0.1	118 (36%)	98% after 4 h
**Mo-6**@SiO_2	0.02	254 (15%)	97% after 8 h
**Mo-6**@SiO_2	0.005	225 (7%)	94% after 8 h

[a] TOF at 3 min, given in min^{-1} with the corresponding conversions given in brackets. [b] In cases where conversion was not reached, the maximum conversion measured after a given time is provided. [c] Maximum conversion is 100%. [d] Experiment performed in *o*-dichlorobenzene.

Plots of Conversion vs Time and Selectivity vs Time for *Cis*-4-Nonene Experiments

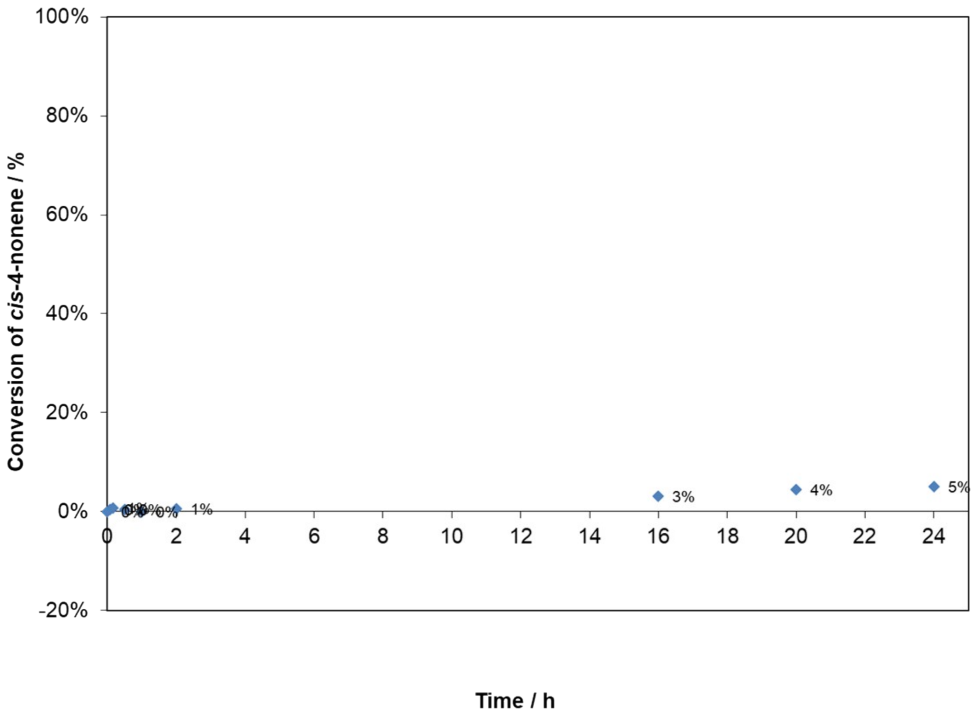

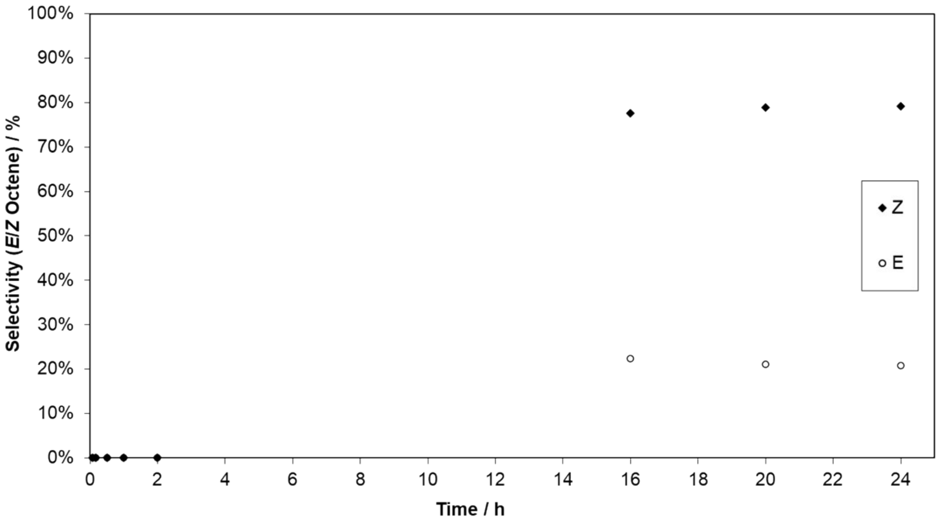

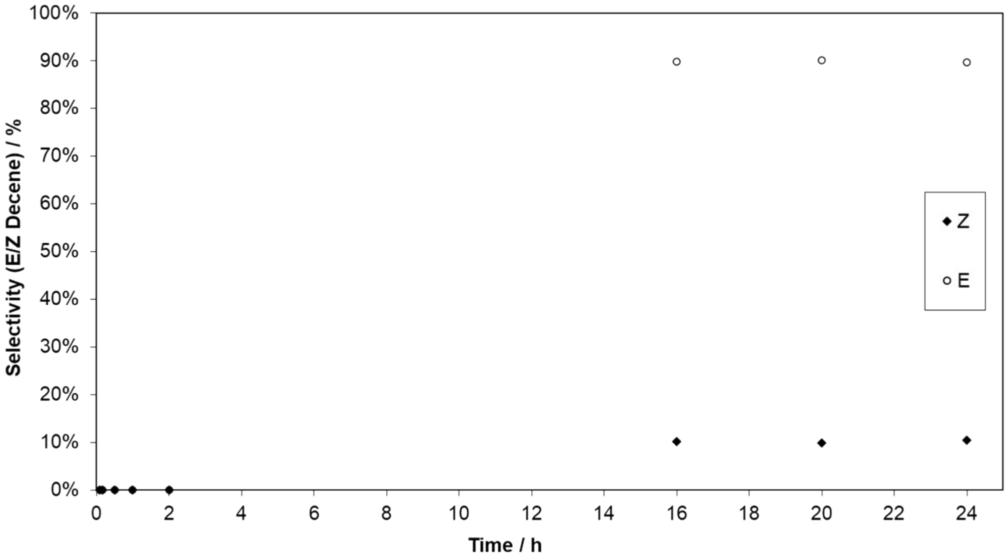

Figure S4. Conversion vs time and selectivity vs time plots for **Mo-6** (0.1 mol%) in *o*-dichlorobenzene.

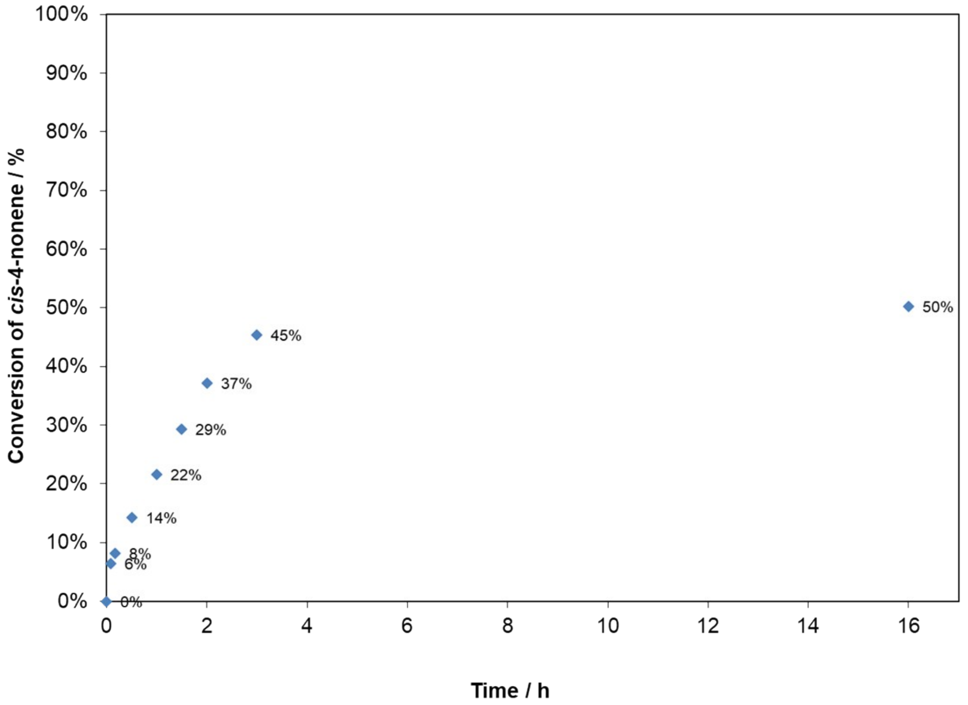
Conversion of *cis*-4-nonene / %
Time / h
100%
90%
80%
70%
60%
50%
40%
30%
20%
10%
0%
0
2
4
6
8
10
12
14
16
0%
6%
8%
14%
22%
29%
37%
45%
50%

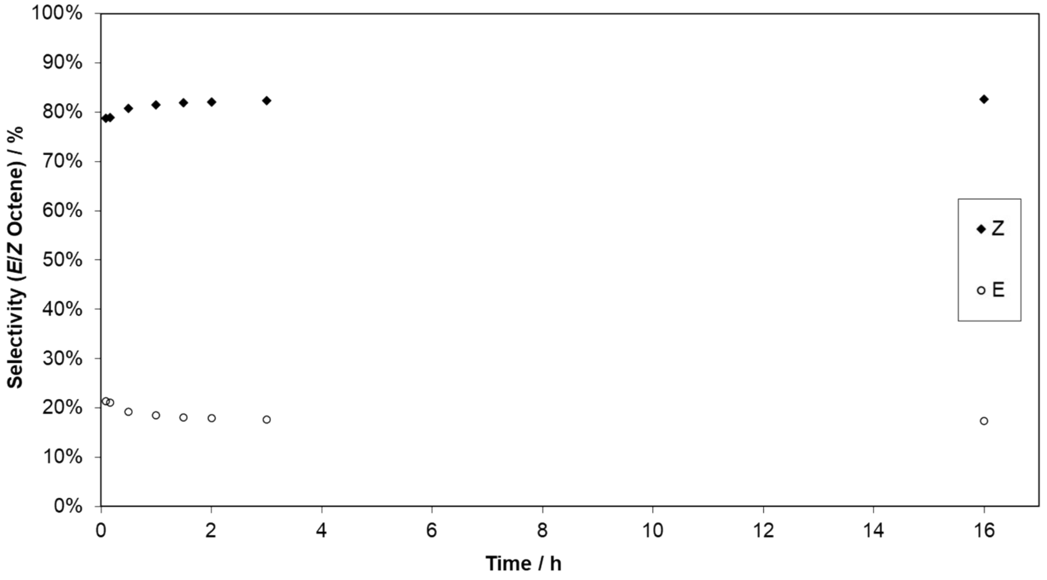
Selectivity (*E*/*Z* Octene) / %
Time / h
100%
90%
80%
70%
60%
50%
40%
30%
20%
10%
0%
0
2
4
6
8
10
12
14
16
Z
E

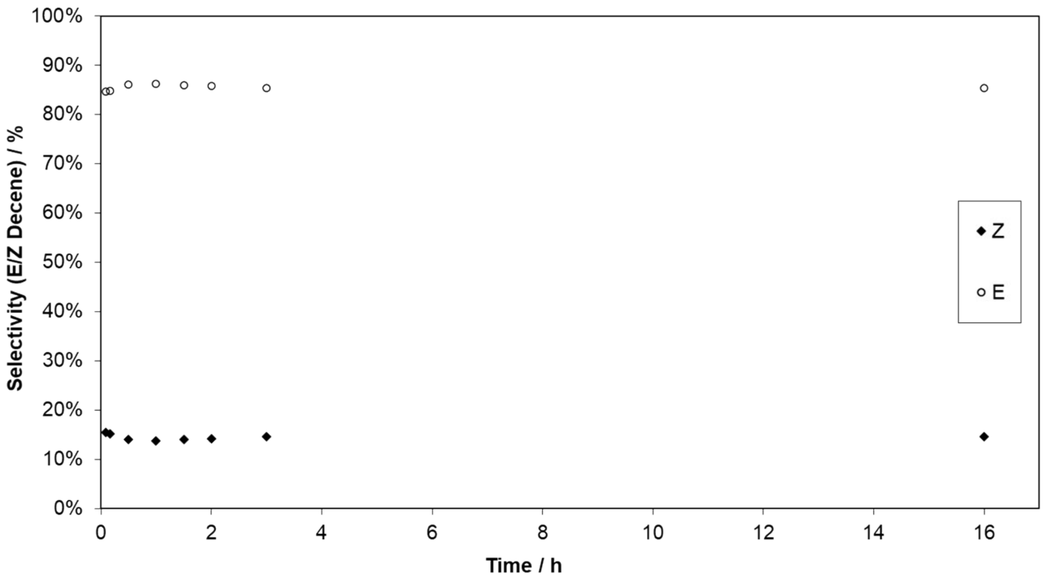

Figure S5. Conversion vs time and selectivity vs time plots for **Mo-6**@SiO_2 (0.1 mol%) in *o*-dichlorobenzene.

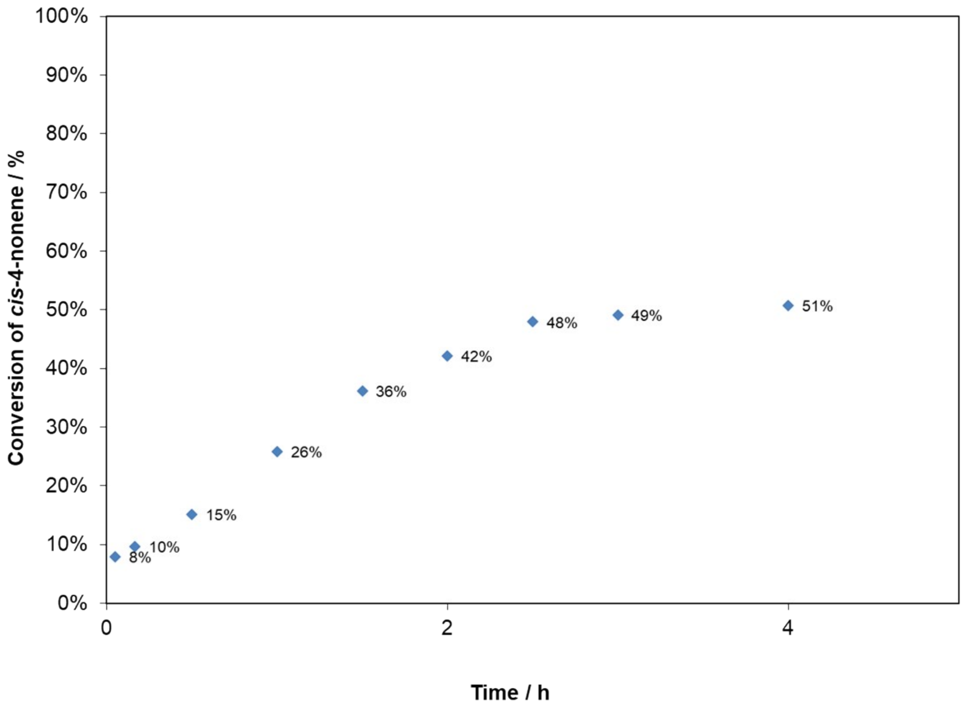

Conversion of *cis*-4-nonene / %
100%
90%
80%
70%
60%
50%
40%
30%
20%
10%
0%
8%
10%
15%
26%
36%
42%
48%
49%
51%
0
2
4
Time / h

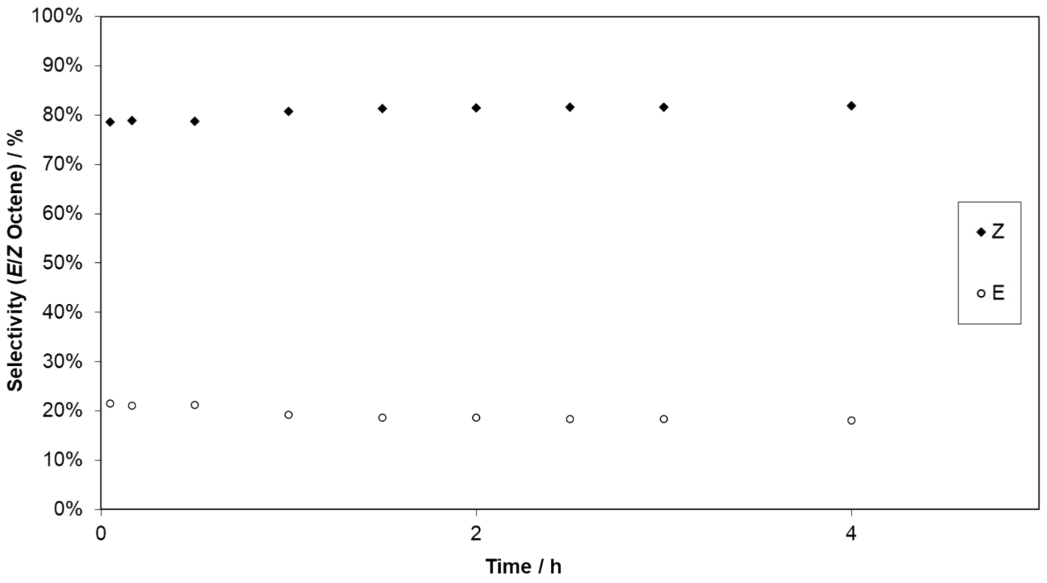

Selectivity (*E*/*Z* Octene) / %
100%
90%
80%
70%
60%
50%
40%
30%
20%
10%
0%
Z
E
0
2
4
Time / h

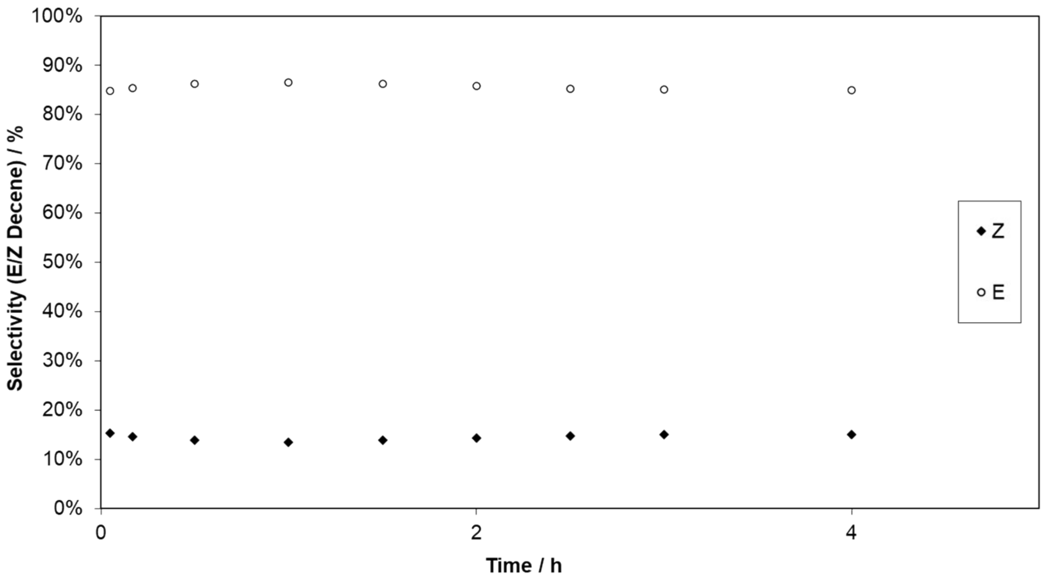

Figure S6. Conversion vs time and selectivity vs time plots for **Mo-6**@SiO_2 (0.1 mol%) in toluene.

Plots of Conversion vs Time and Selectivity vs Time for 1-Nonene Experiments

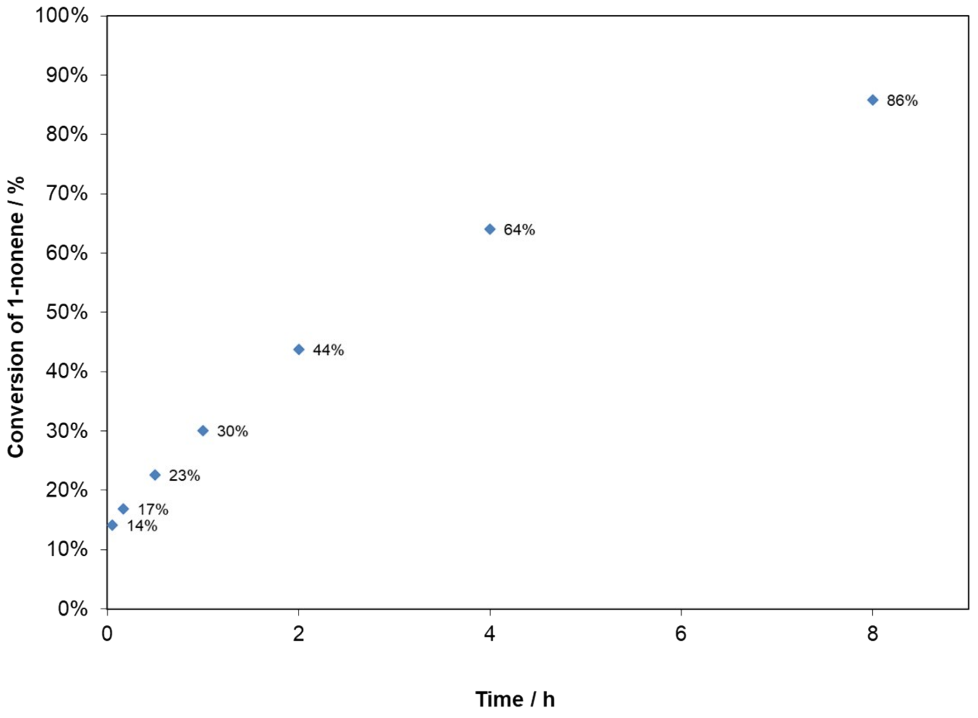

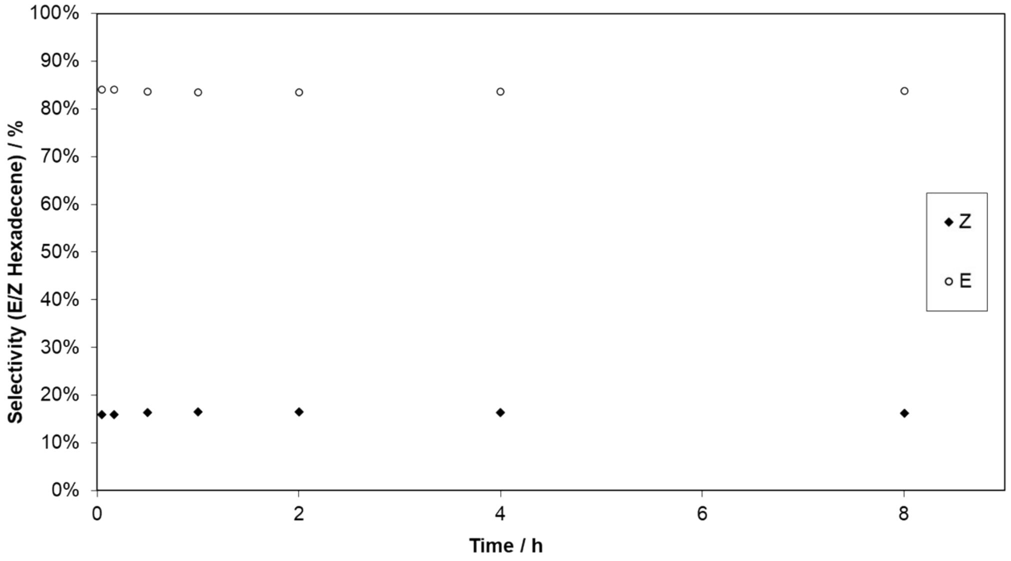

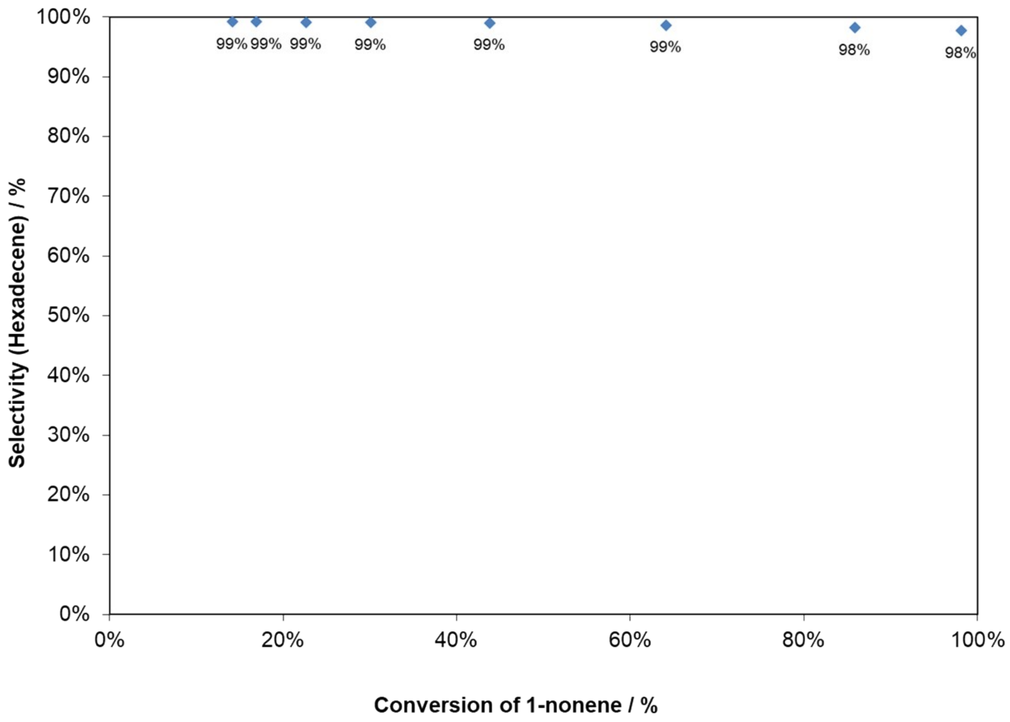

Figure S7. Conversion vs time, selectivity vs time and conversion vs selectivity plots for **Mo-6** (0.1 mol%) in *o*-dichlorobenzene.

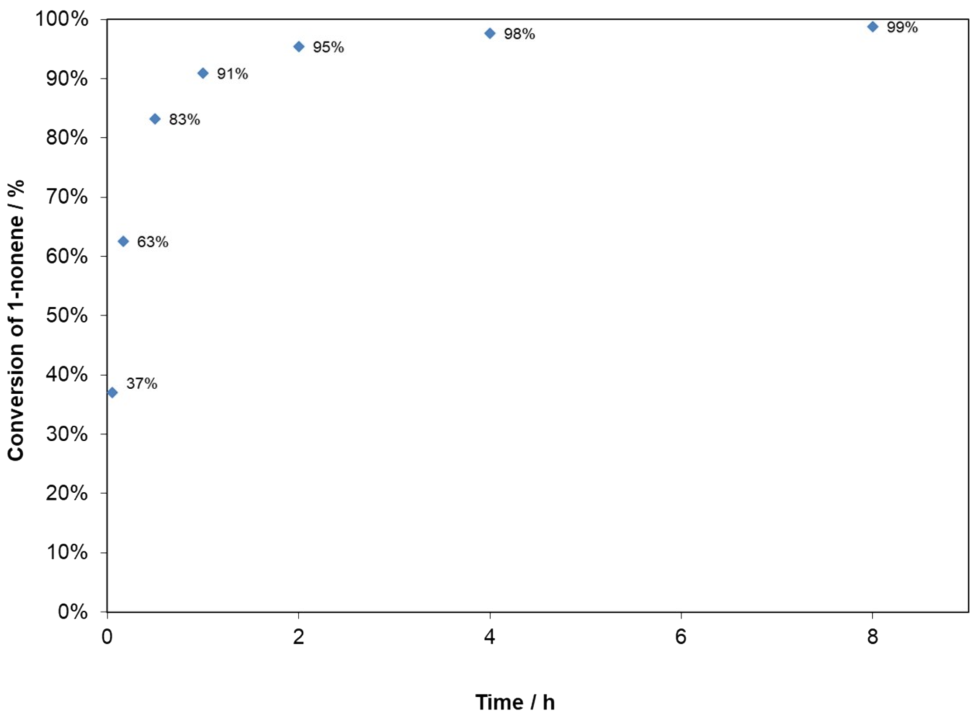
Conversion of 1-nonene / %
Time / h
37%
63%
83%
91%
95%
98%
99%

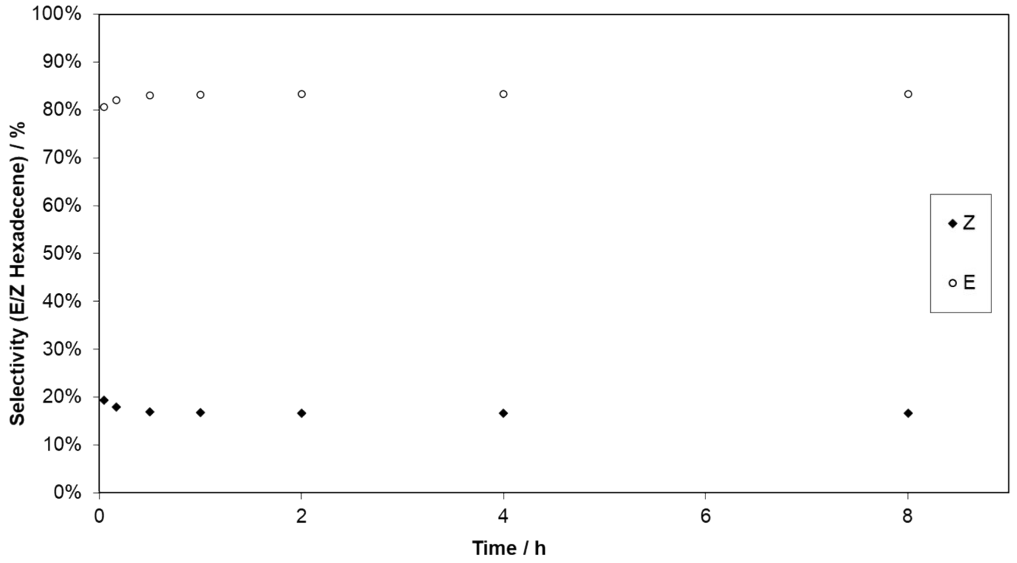
Selectivity (E/Z Hexadecene) / %
Time / h
Z
E

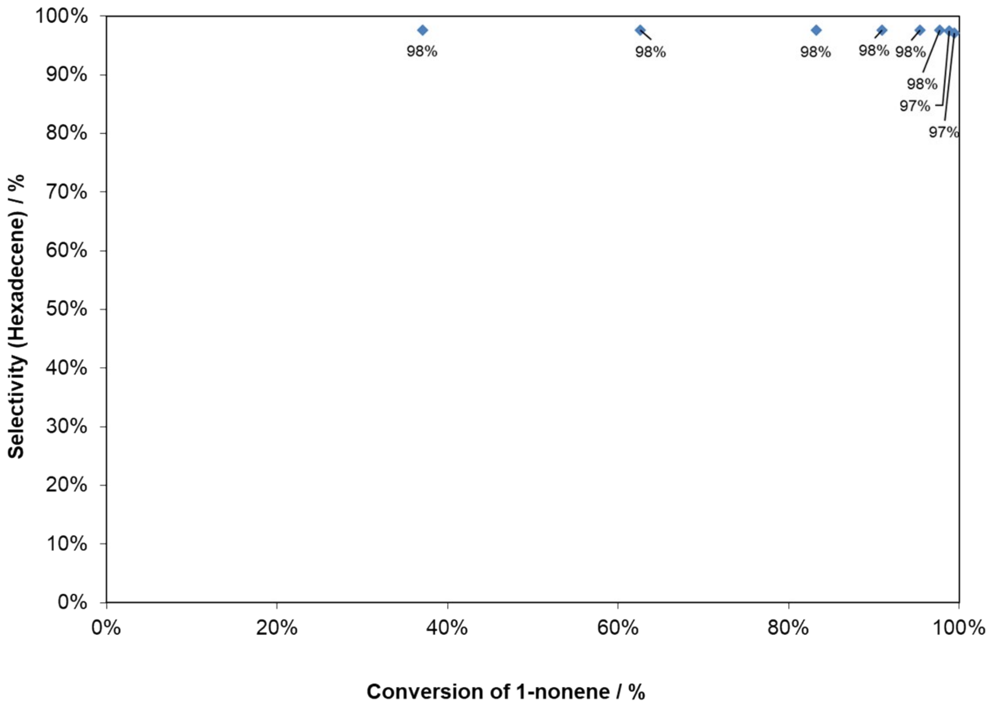

Figure S8. Conversion vs time, selectivity vs time and conversion vs selectivity plots for **Mo-6**@SiO_2 (0.1 mol%) in *o*-dichlorobenzene.

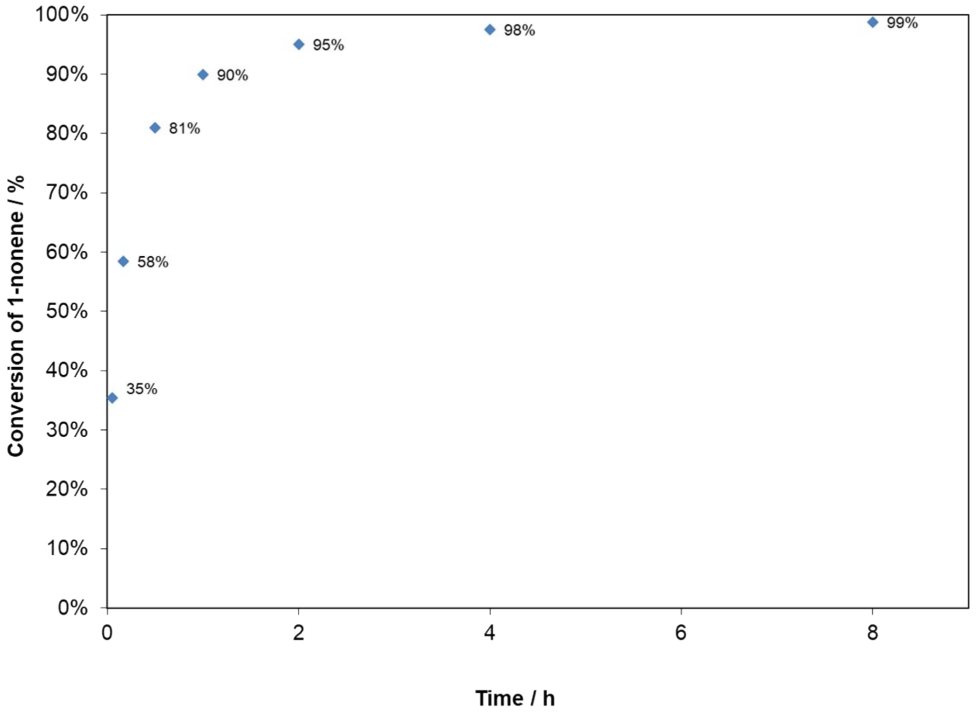

Conversion of 1-nonene / %
Time / h
100%
90%
80%
70%
60%
50%
40%
30%
20%
10%
0%
0
2
4
6
8
35%
58%
81%
90%
95%
98%
99%

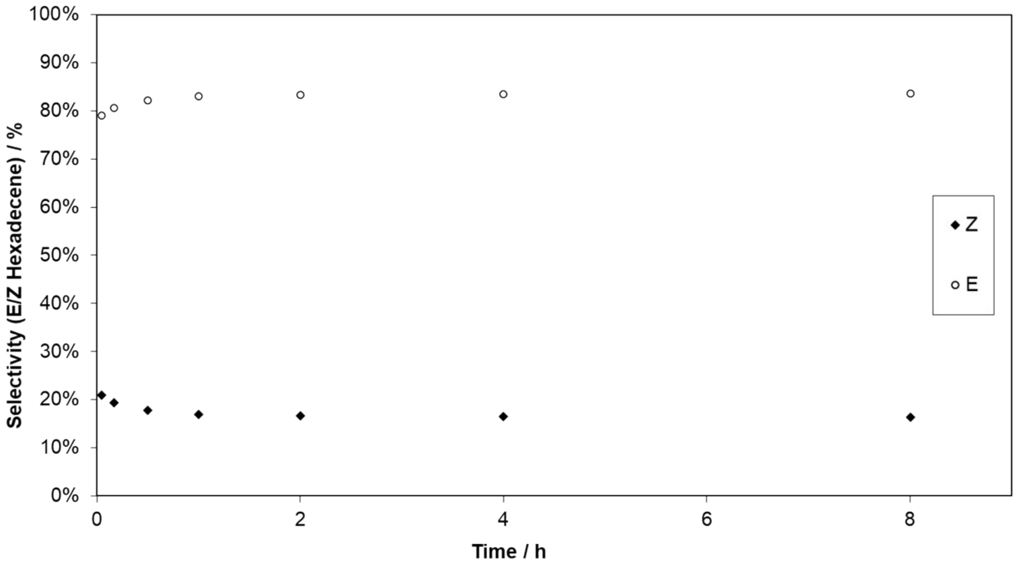

Selectivity (E/Z Hexadecene) / %
Time / h
100%
90%
80%
70%
60%
50%
40%
30%
20%
10%
0%
0
2
4
6
8
Z
E

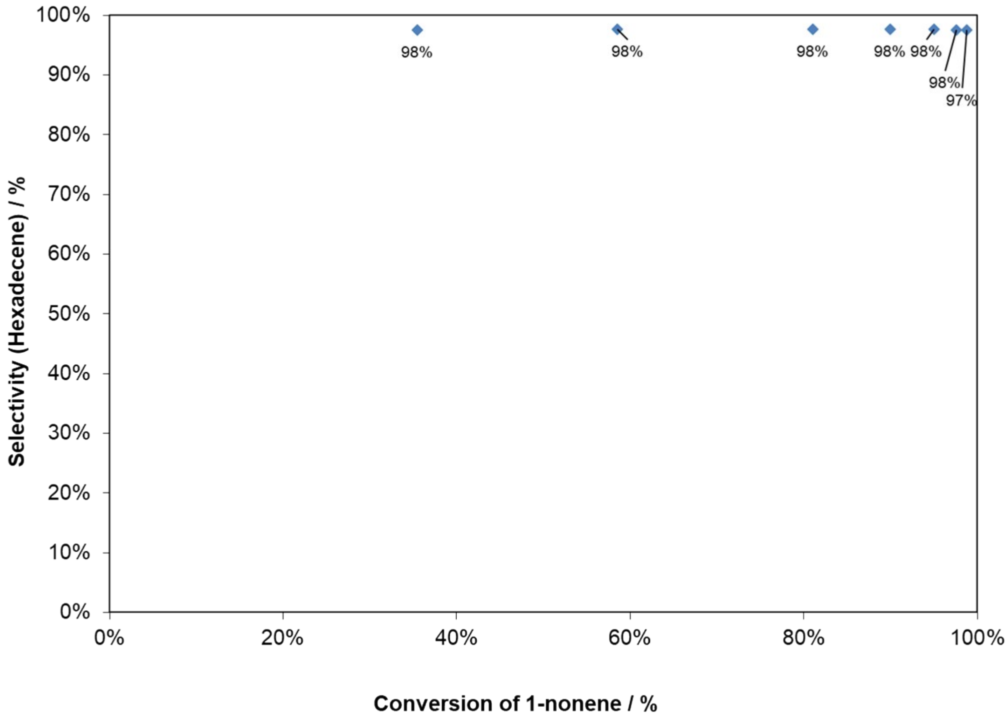

Figure S9. Conversion vs time, selectivity vs time and conversion vs selectivity plots for **Mo-6**@SiO_2 (0.1 mol%) in toluene.

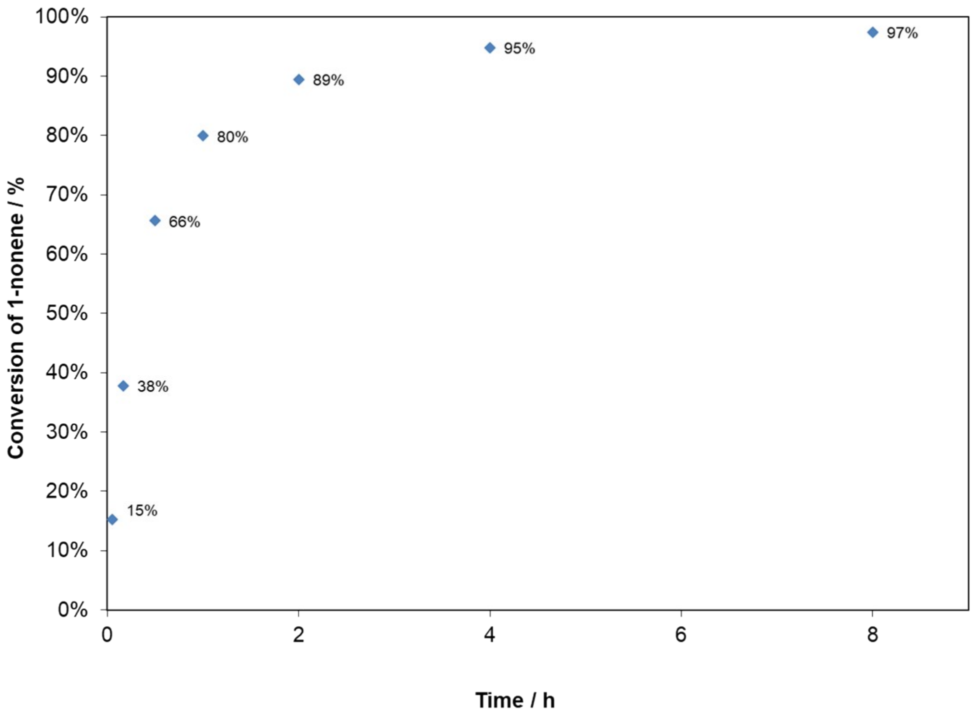
100%
90%
80%
70%
60%
50%
40%
30%
20%
10%
0%
Conversion of 1-nonene / %
15%
38%
66%
80%
89%
95%
97%
0
2
4
6
8
Time / h

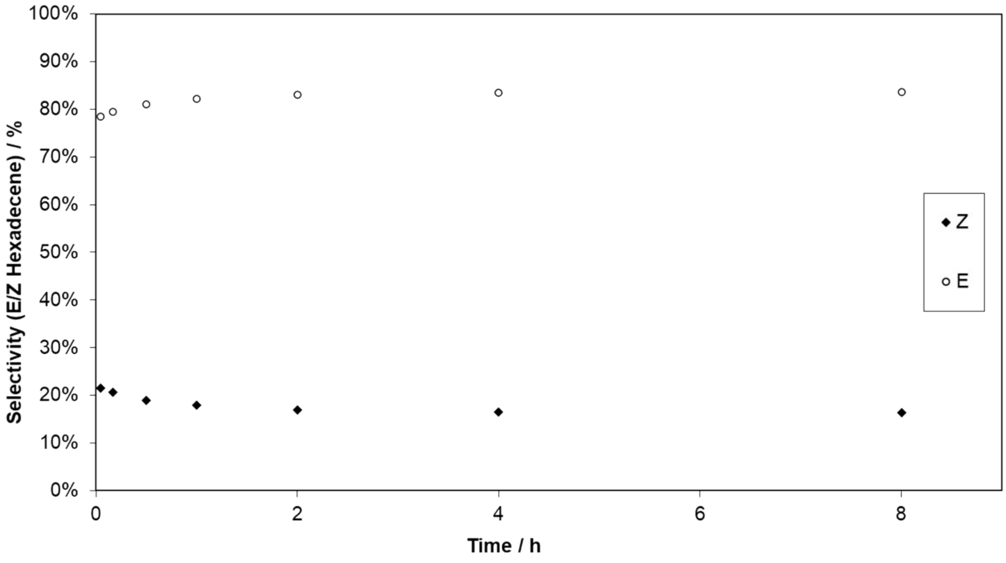
100%
90%
80%
70%
60%
50%
40%
30%
20%
10%
0%
Selectivity (E/Z Hexadecene) / %
Z
E
0
2
4
6
8
Time / h

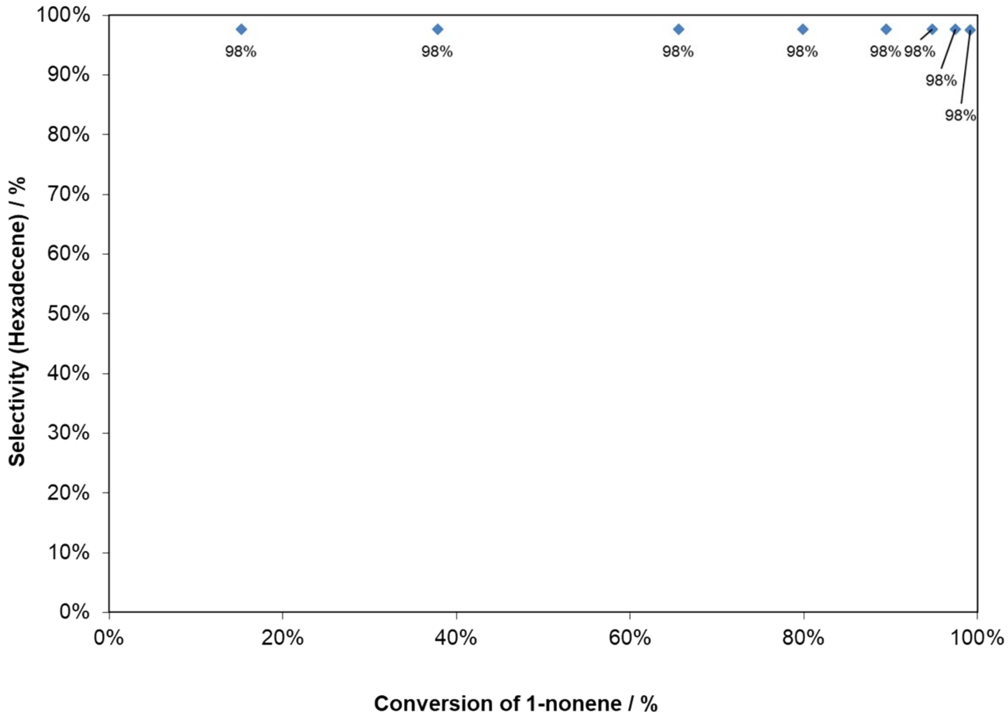

Figure S10. Conversion vs time, selectivity vs time and conversion vs selectivity plots for **Mo-6**@SiO_2 (0.02 mol%) in toluene.

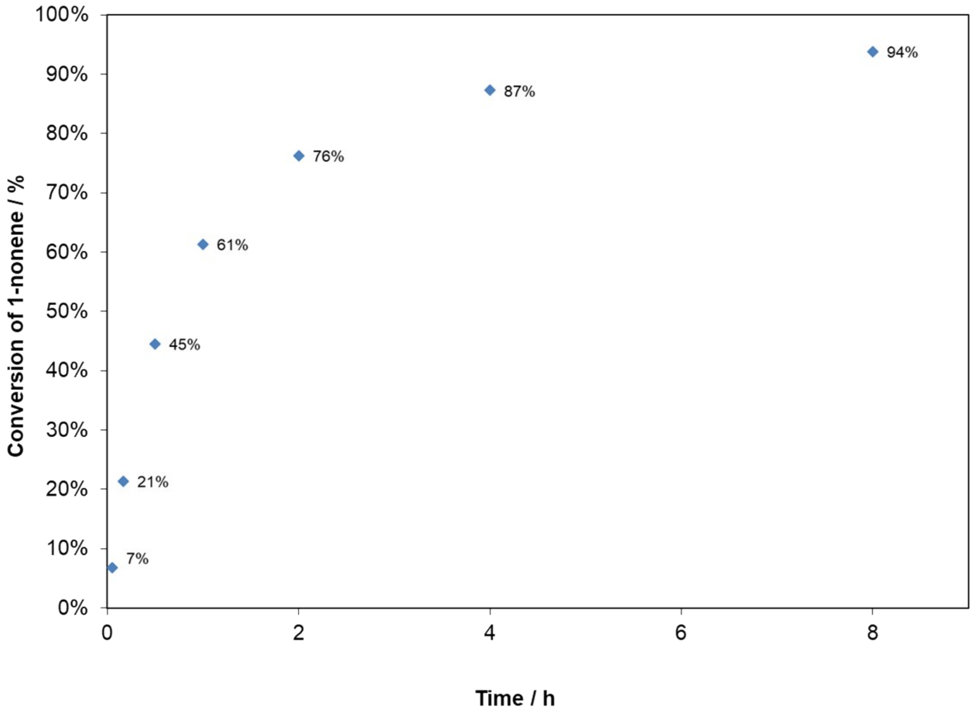

100%
90%
80%
70%
60%
50%
40%
30%
20%
10%
0%
Conversion of 1-nonene / %
94%
87%
76%
61%
45%
21%
7%
0
2
4
6
8
Time / h

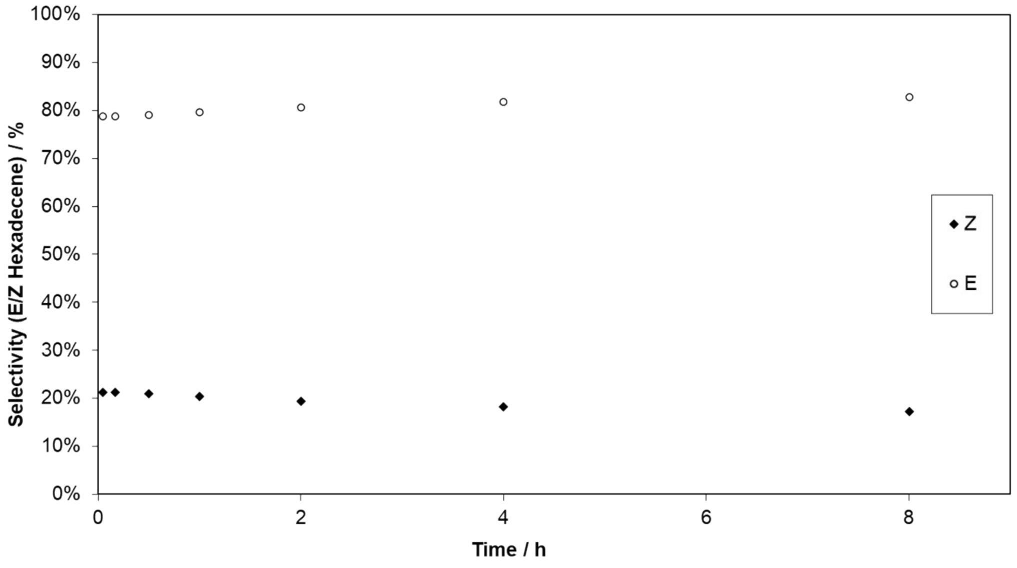

100%
90%
80%
70%
60%
50%
40%
30%
20%
10%
0%
Selectivity (E/Z Hexadecene) / %
Z
E
0
2
4
6
8
Time / h

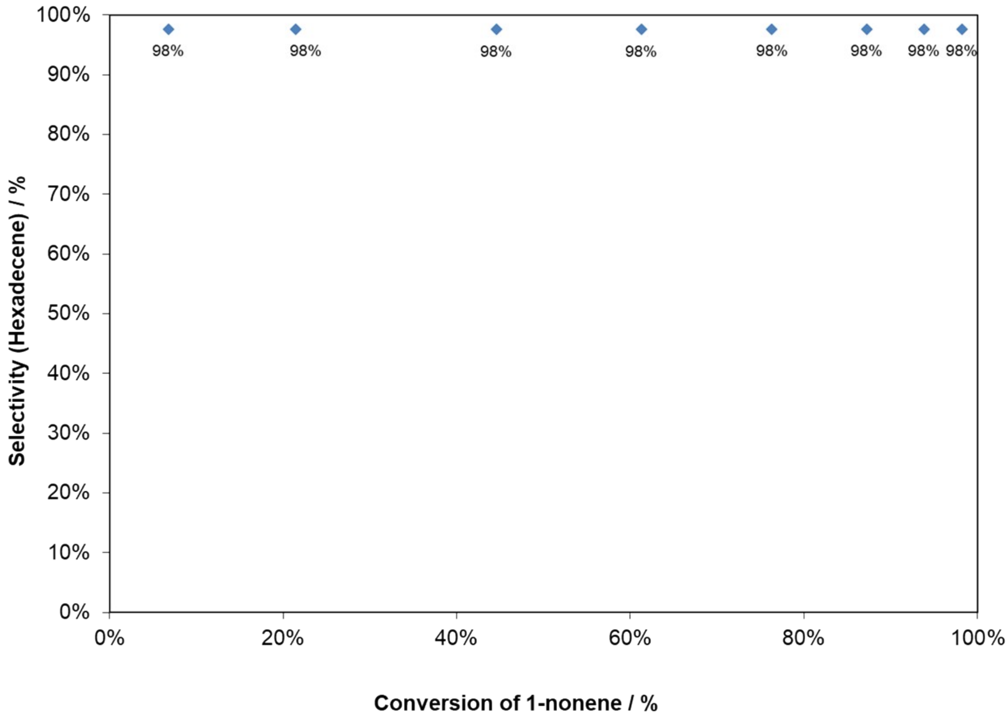

Figure S11. Conversion vs time, selectivity vs time and conversion vs selectivity plots for **Mo-6**@SiO_2 (0.01 mol%) in toluene.

Recycling Tests with 1-Nonene

Every agitation and dispensing step for experiments with 1-nonene was performed by a liquid handling robot (Zinsser Analytics) operated inside a nitrogen-filled glovebox (<0.1 ppm H_2O and O_2). The procedure for 1-nonene experiments (described in Experimental Procedures and Catalytic Data) was repeated three times sequentially with **Mo-6**@SiO_2 (0.1 mol%). Washing steps consisting of aspiration of the reaction solution, addition of 2 mL of toluene, 3 minutes of agitation, waiting 1 minute for the solids to settle and withdrawing 2 mL of washings, were included. This washing procedure was repeated 3 times, before continuing with the next experiment.

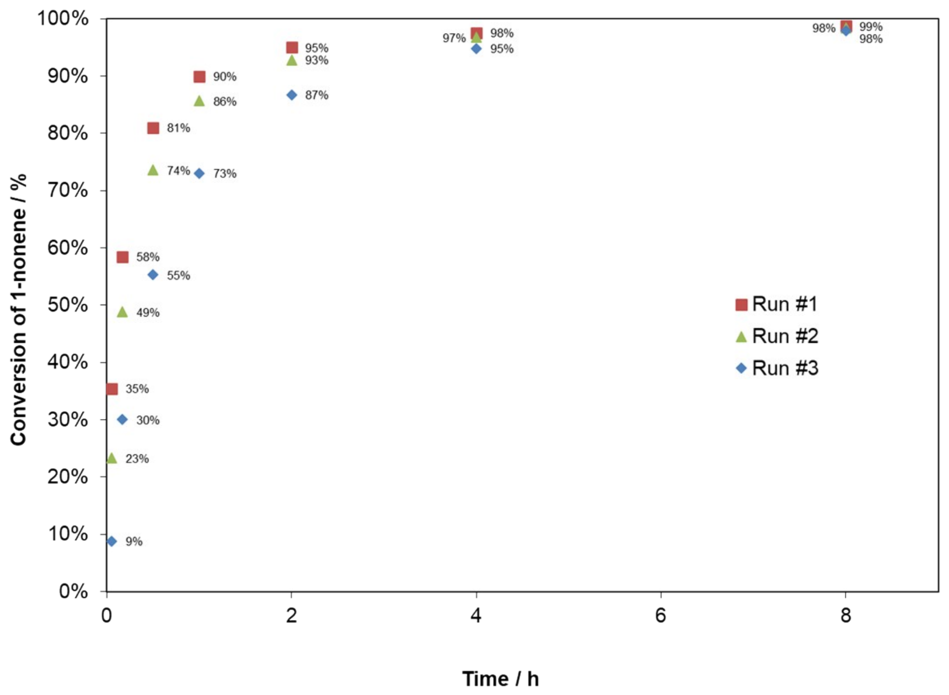

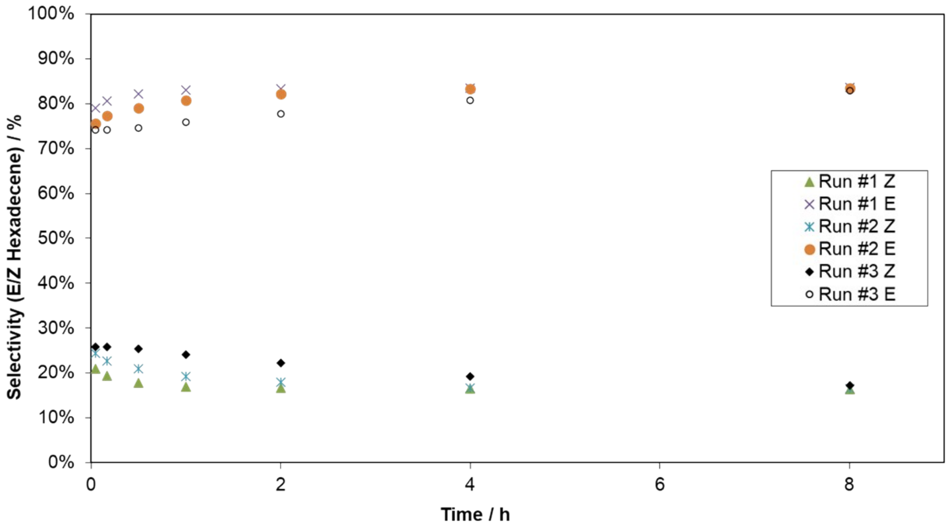

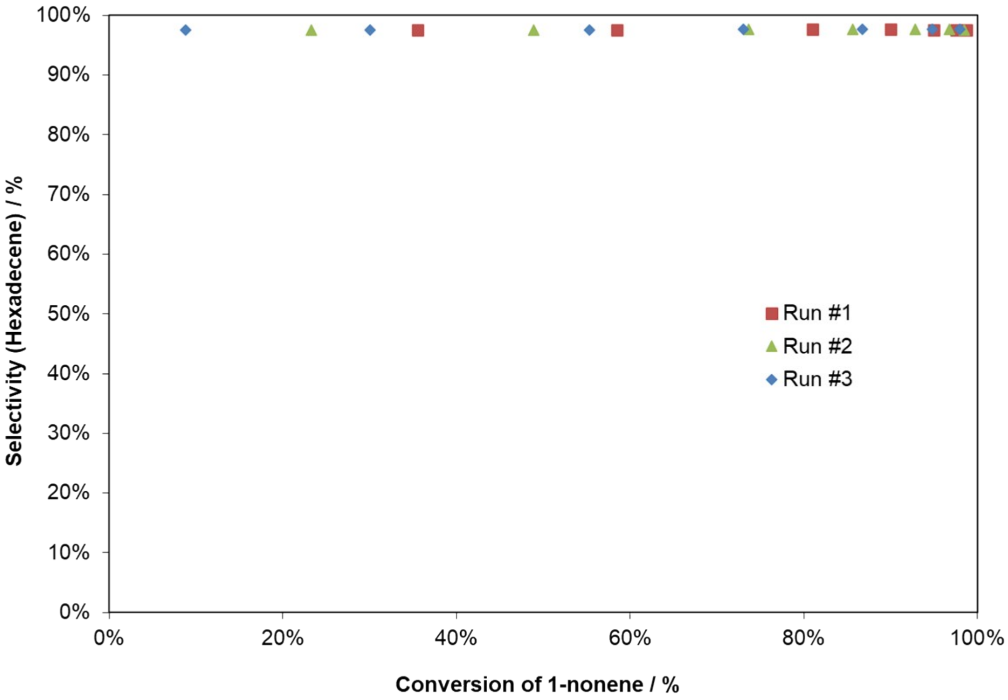

Figure S12. Conversion vs time, selectivity vs time and conversion vs selectivity plots for recycling tests performed three times with **Mo-6**@SiO_2 (0.1 mol%) in toluene.

Table S3. Homometathesis of 1-nonene with **Mo-6**@SiO_2 (0.1 mol%, toluene, 30 °C).

Run[a]	***TOF***$_{3min}$[a]	***Time to maximum conversion***[b][c]
#1	118 (35%)	98% after 4 h
#2	78 (23%)	97% after 4 h
#3	29 (9%)	98% after 8 h

[a] TOF at 3 min, given in min^{-1} with the corresponding conversions given in brackets. [b] In cases where full conversion was not reached, the maximum conversion measured after a given time is provided. [c] Maximum conversion is 100%.

3. Solution NMR Spectra

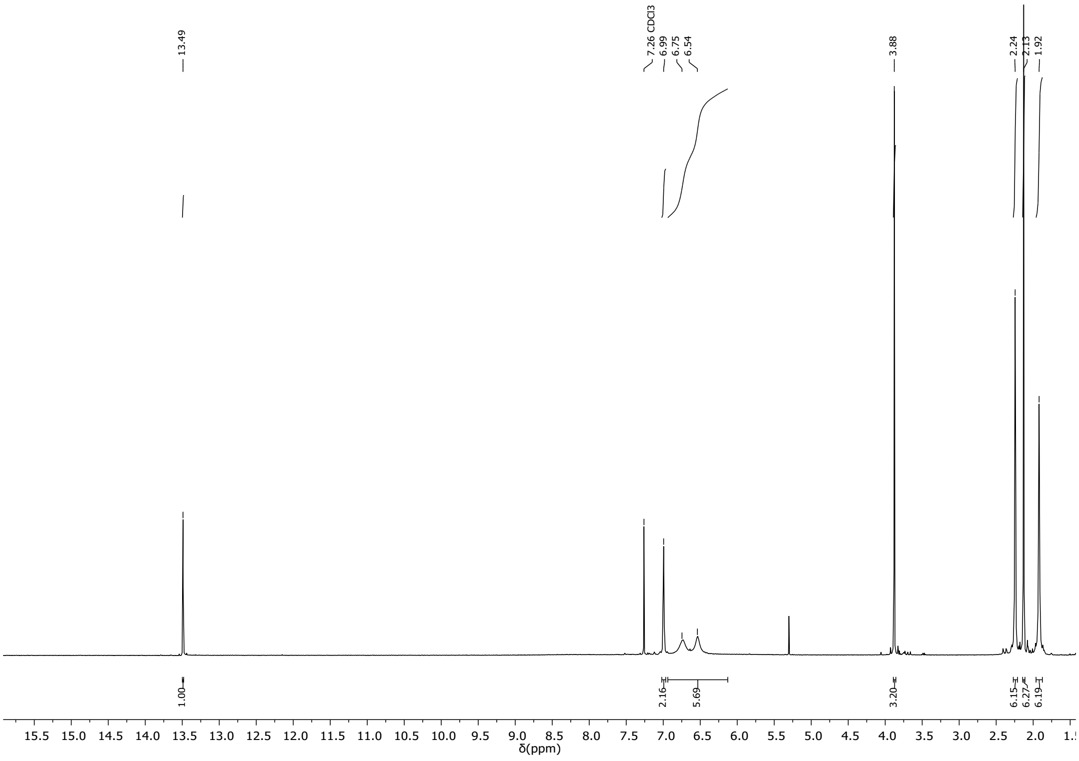

Figure S13. ^{1}H-NMR spectrum (400 MHz, $CDCl_3$, 25 °C) of [$MoO(CH\text{-}4\text{-}OMeC_6H_4)(OC(CF_3)_3)_2(IMesCl_2)$] (**Mo-2**).

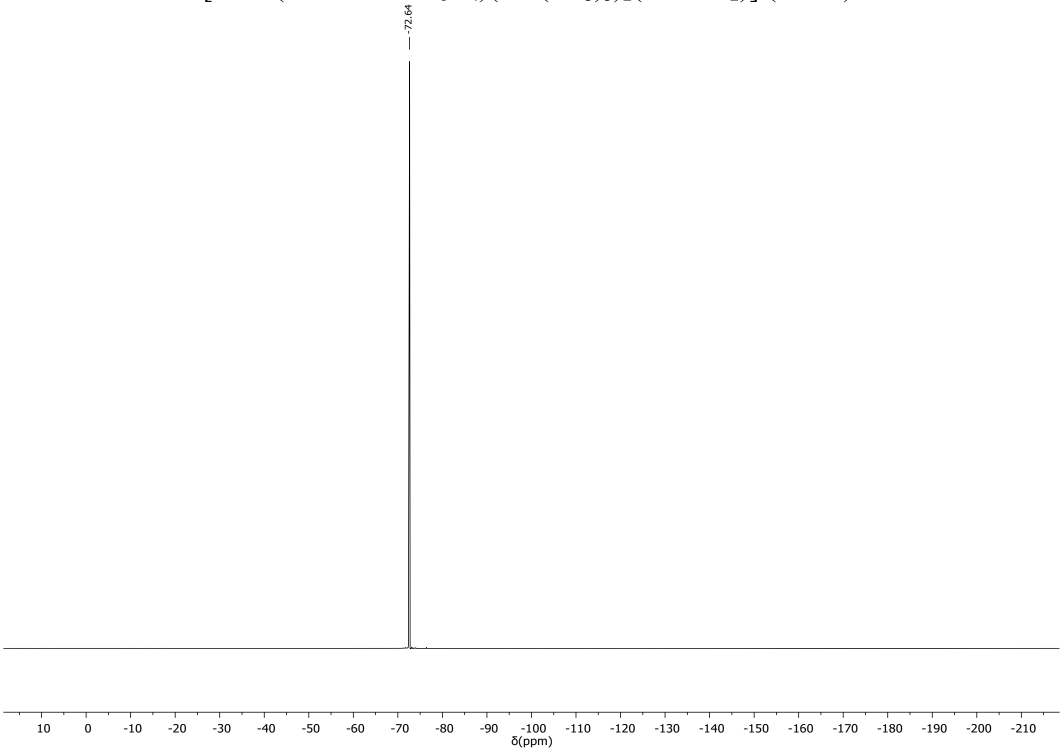

Figure S14. ^{19}F-NMR spectrum (375 MHz, $CDCl_3$, 25 °C) of [$MoO(CH\text{-}4\text{-}OMeC_6H_4)(OC(CF_3)_3)_2(IMesCl_2)$] (**Mo-2**).

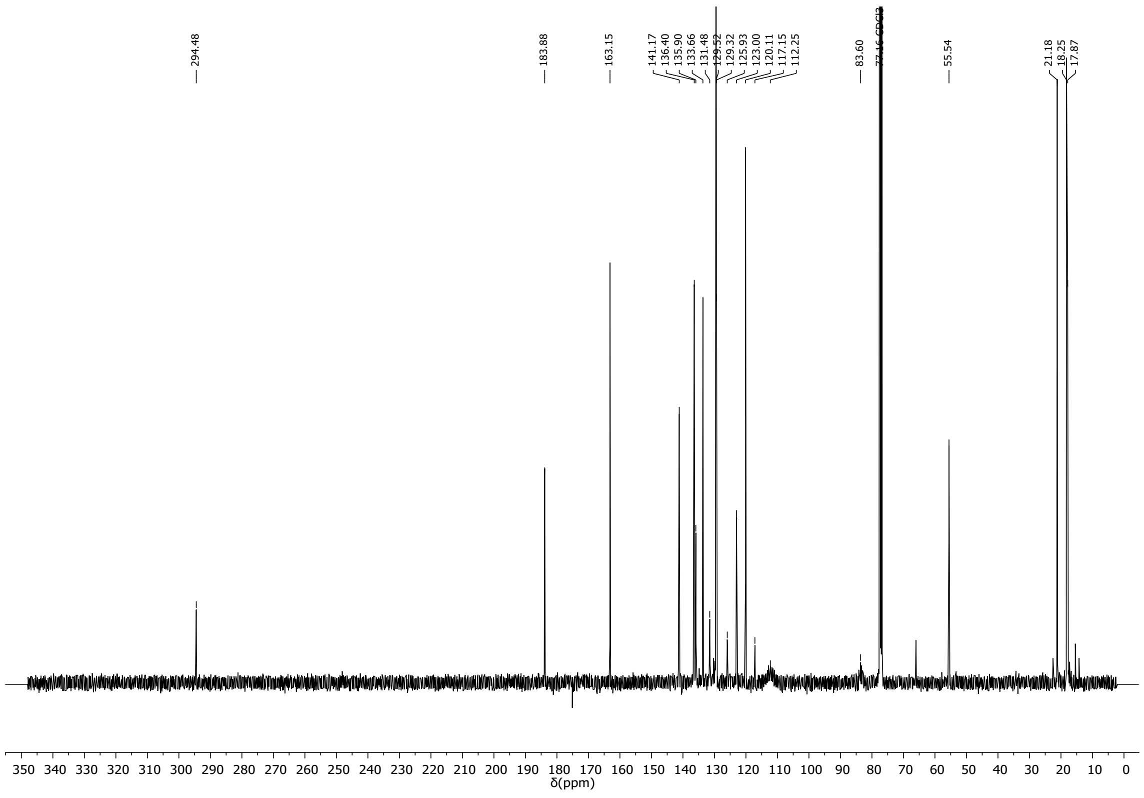

Figure S15. ^{13}C-NMR spectrum (100 MHz, $CDCl_3$, 25 °C) of [MoO(CH-4-OMeC_6H_4)(OC$(CF_3)_3)_2$(IMesCl_2)] (**Mo-2**).

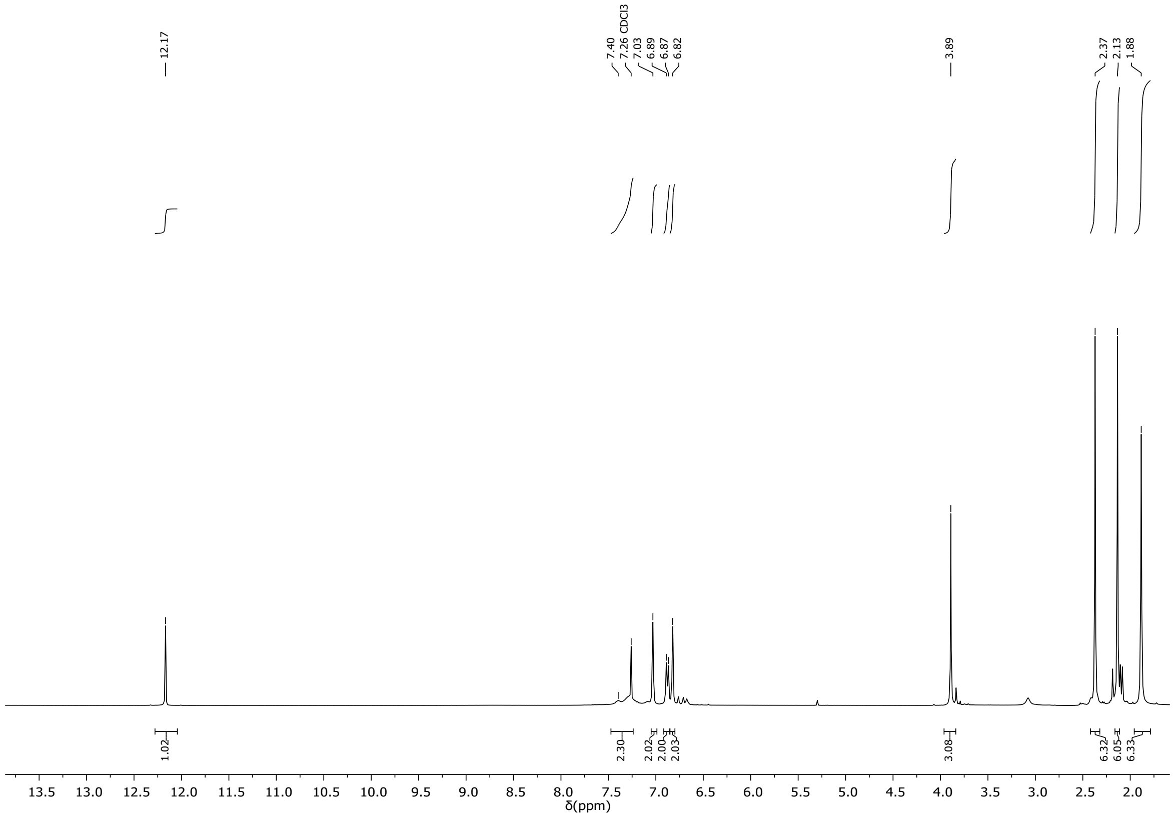

Figure S16. ^{1}H-NMR spectrum (400 MHz, $CDCl_3$, 25 °C) of

[MoO(CH-4-OMeC$_6$H$_4$)Cl$_2$(IMesCl$_2$)] (**Mo-3**).

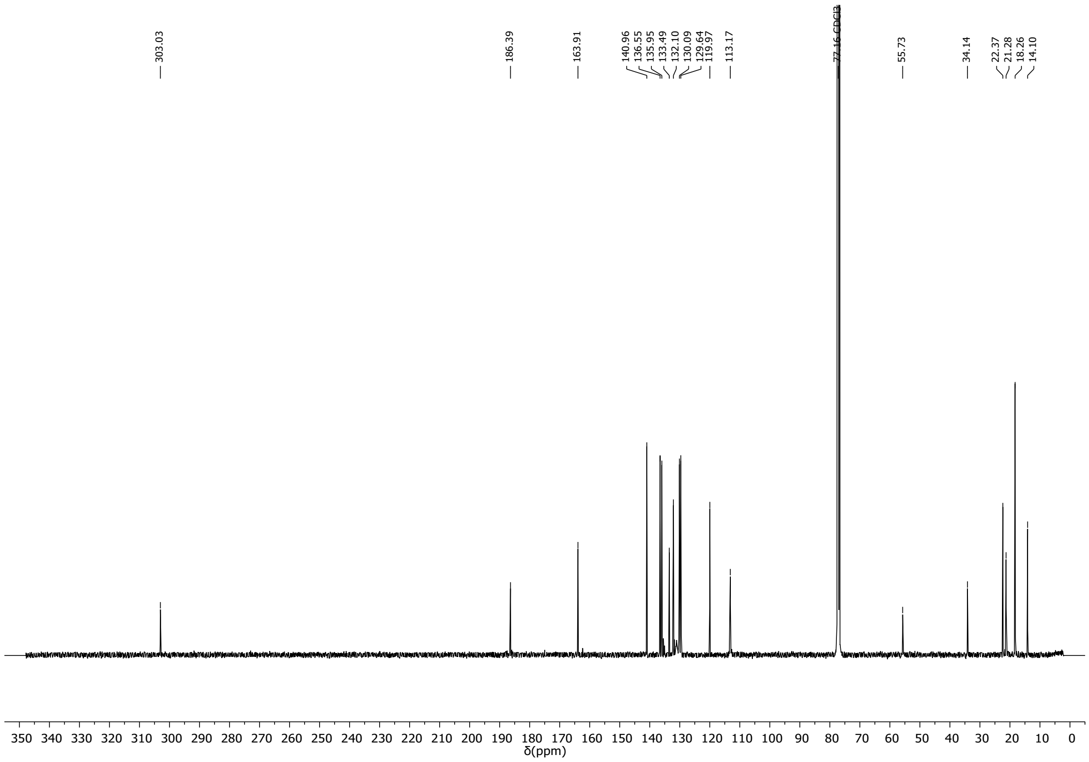

Figure S17. ^{13}C-NMR spectrum (100 MHz, CDCl$_3$, 25 °C) of [MoO(CH-4-OMeC$_6$H$_4$)Cl$_2$(IMesCl$_2$)] (**Mo-3**).

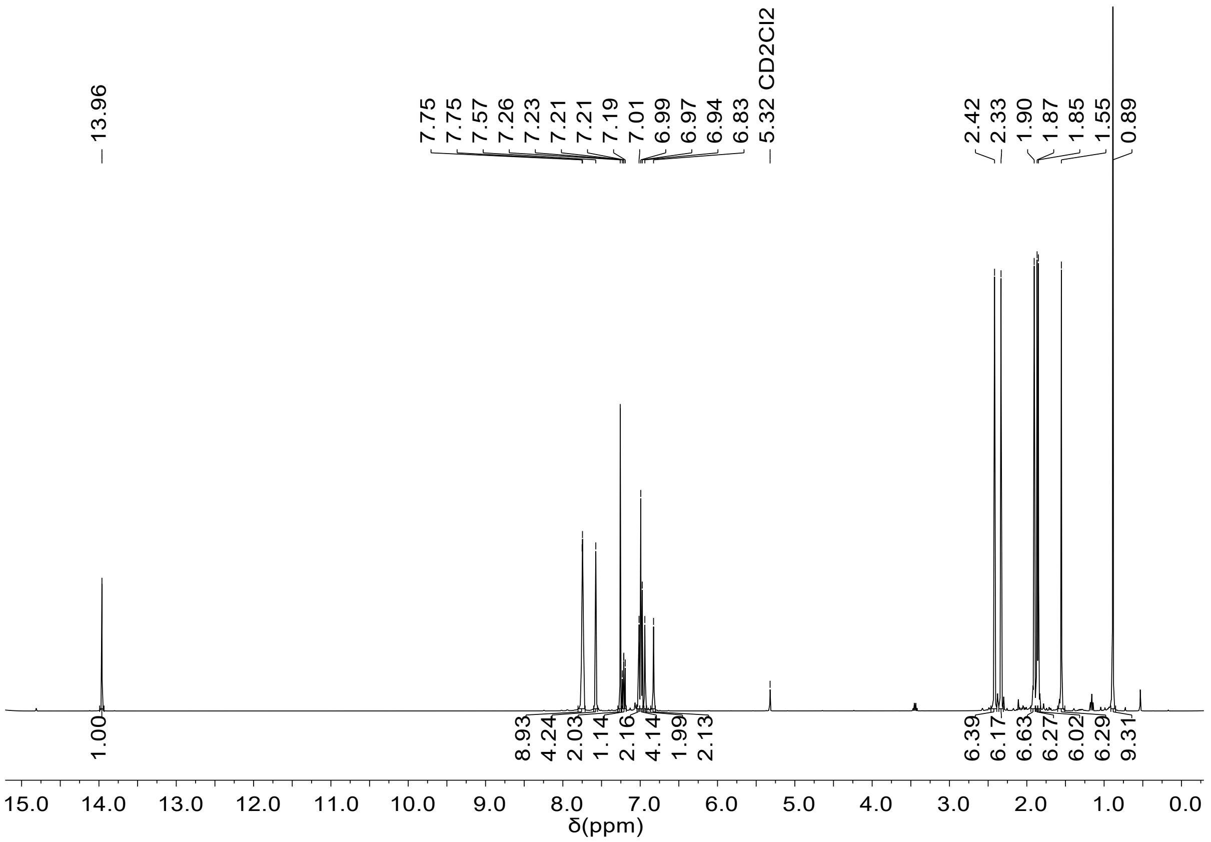

Figure S18. ^{1}H-NMR spectrum (400 MHz, CD_2Cl_2, 25 °C) of [MoO(CHCMe$_3$)(OHMT)(IMes)][B(Ar^F)$_4$] (**Mo-6**).

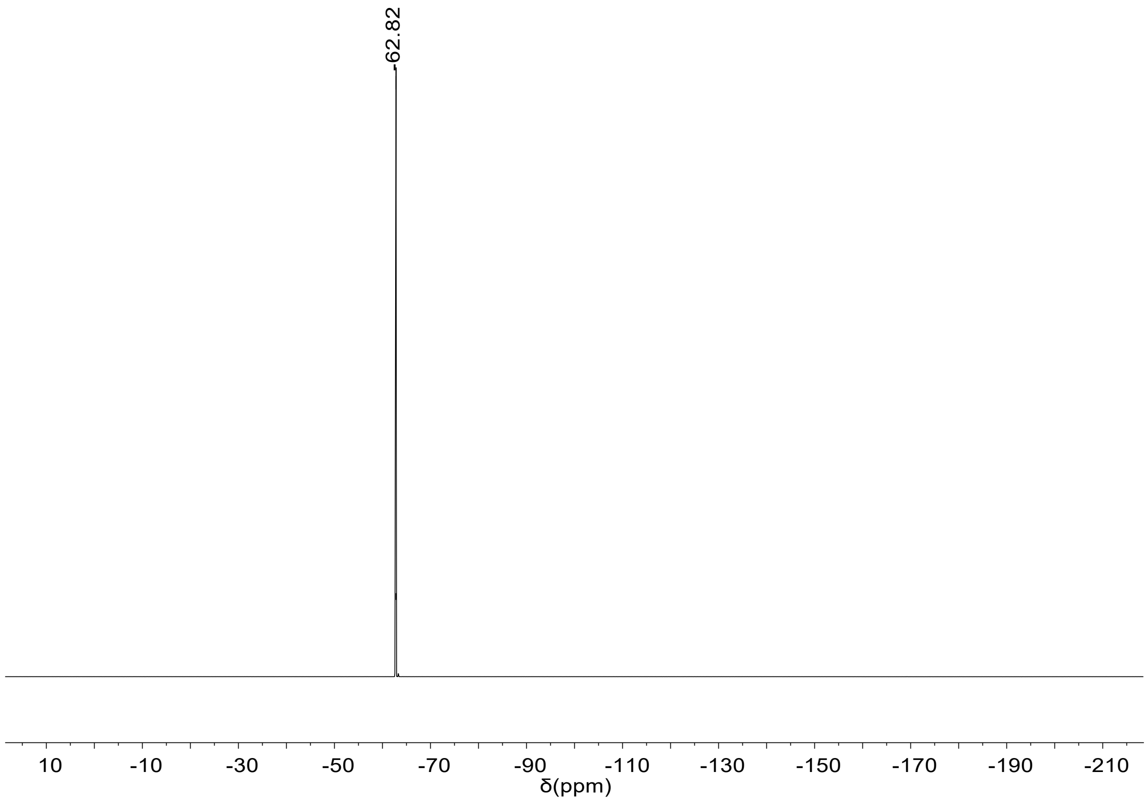

Figure S19. ^{19}F-NMR spectrum (375 MHz, CD_2Cl_2, 25 °C) of [MoO($CHCMe_3$)(OHMT)(IMes)][B(Ar^F)$_4$] (**Mo-6**).

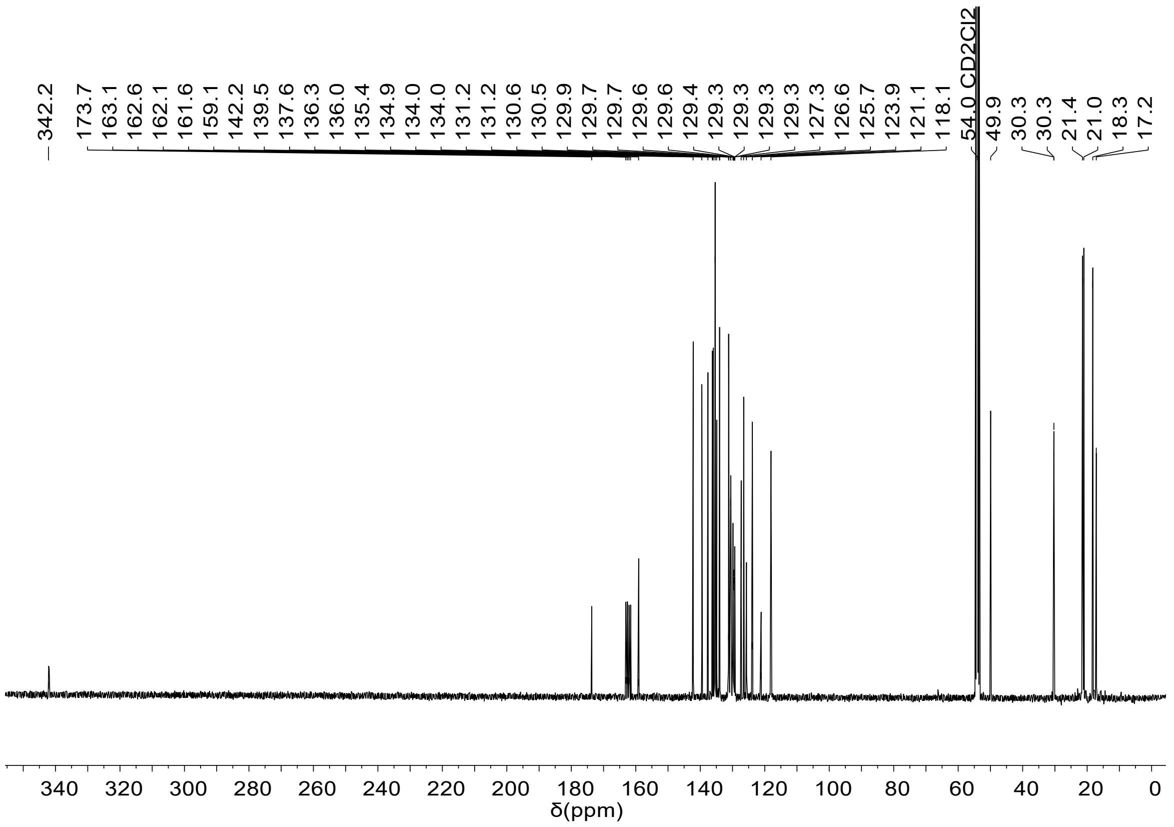

Figure S20. ^{13}C-NMR spectrum (100 MHz, CD_2Cl_2, 25 °C) of [MoO(CHCMe$_3$)(OHMT)(IMes)][B(Ar^F)$_4$] (**Mo-6**).

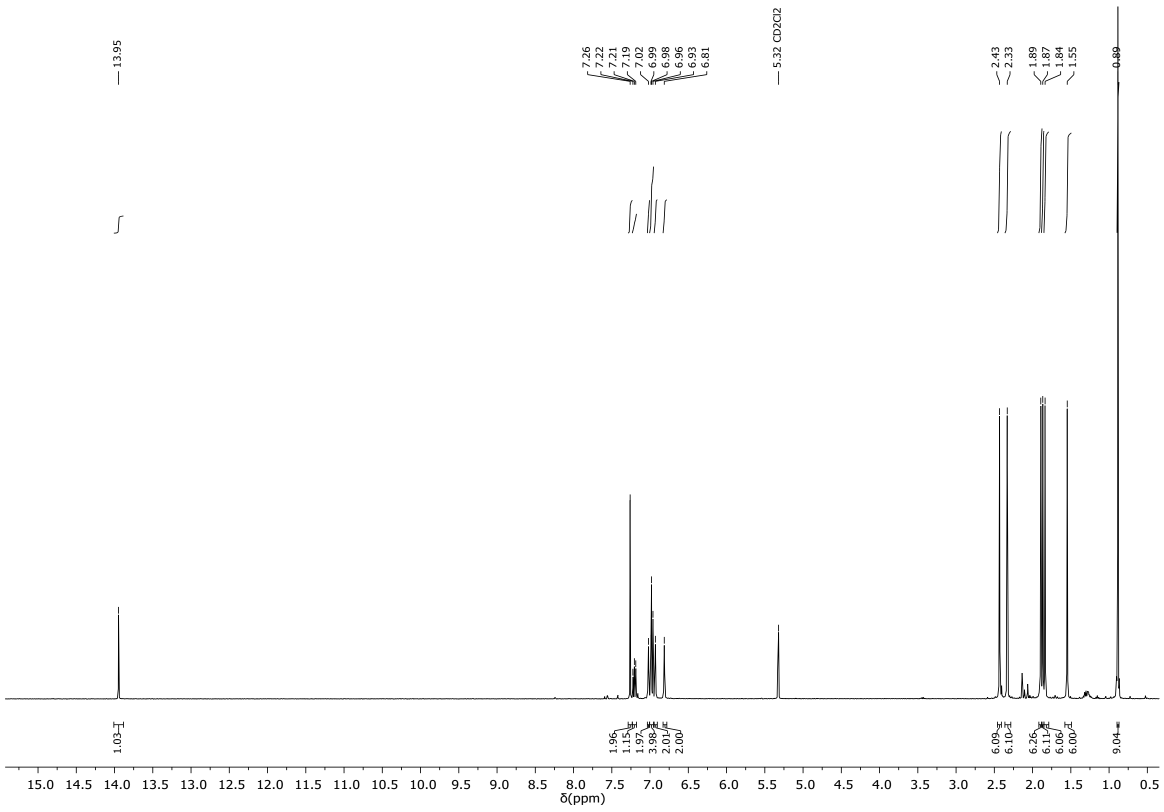

Figure S21. ^{1}H-NMR spectrum (375 MHz, CD_2Cl_2, 25 °C) of $[MoO(CHCMe_3)(OHMT)(IMes)][Al(OC_4F_9)_4]$ (**Mo-7**).

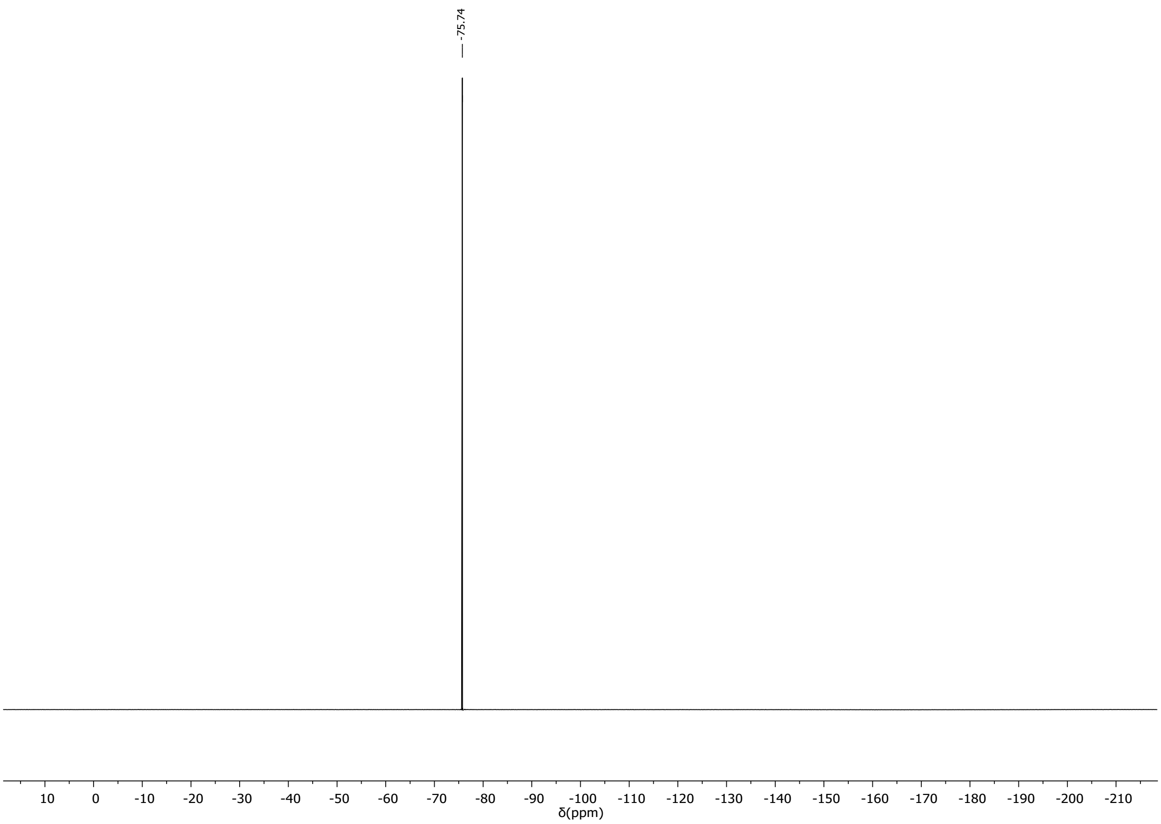

Figure S22. ^{19}H-NMR spectrum (400 MHz, CD_2Cl_2, 25 °C) of [MoO(CHCMe$_3$)(OHMT)(IMes)][Al(OC$_4$F$_9$)$_4$] (**Mo-7**).

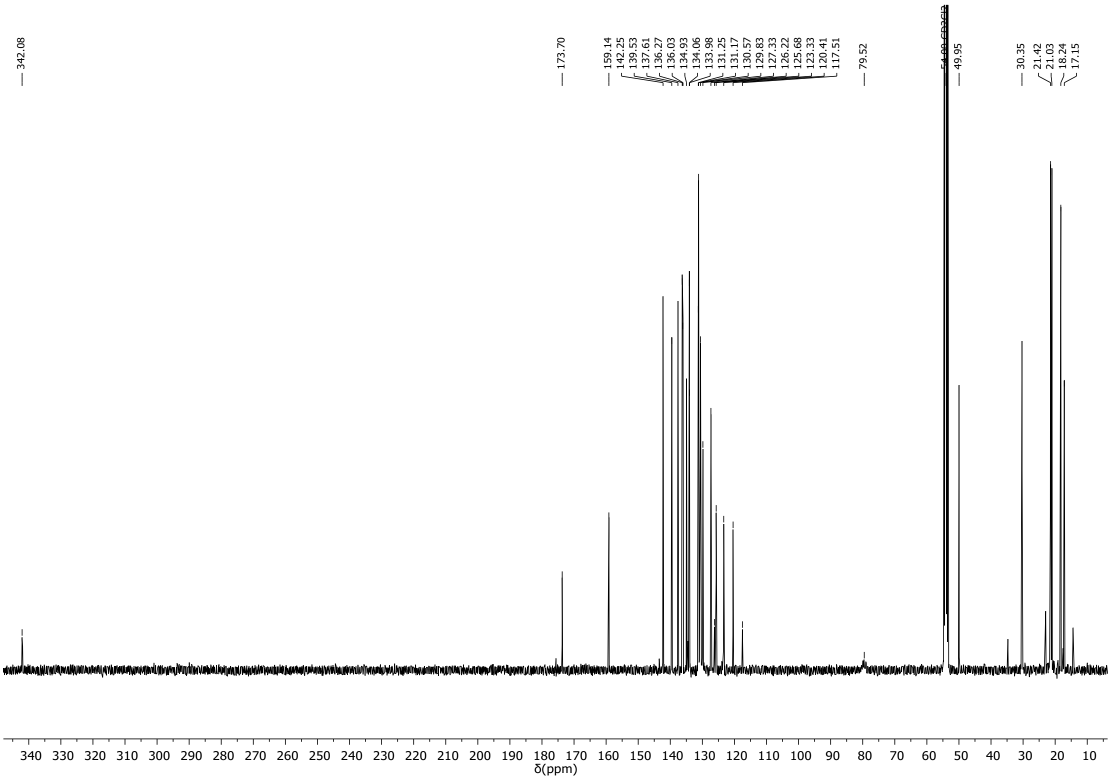

Figure S23. ^{13}C-NMR spectrum (100 MHz, CD_2Cl_2, 25 °C) of [MoO(CHCMe$_3$)(OHMT)(IMes)][Al(OC$_4$F$_9$)$_4$] (**Mo-7**).

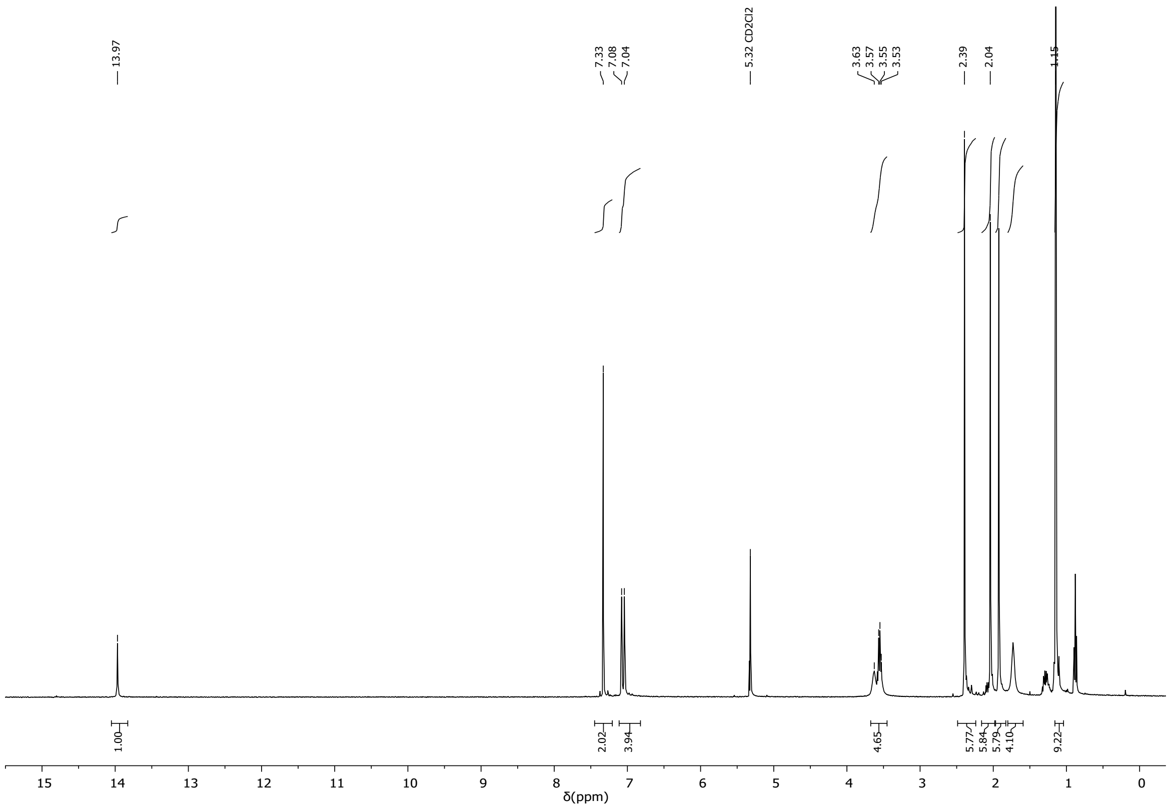

Figure S24. ^{1}H-NMR spectrum (400 MHz, CD_2Cl_2, 25 °C) of $[MoO(CHCMe_3)(OC_6F_5)(IMes)(THF)][Al(OC_4F_9)_4]$ (**Mo-8**).

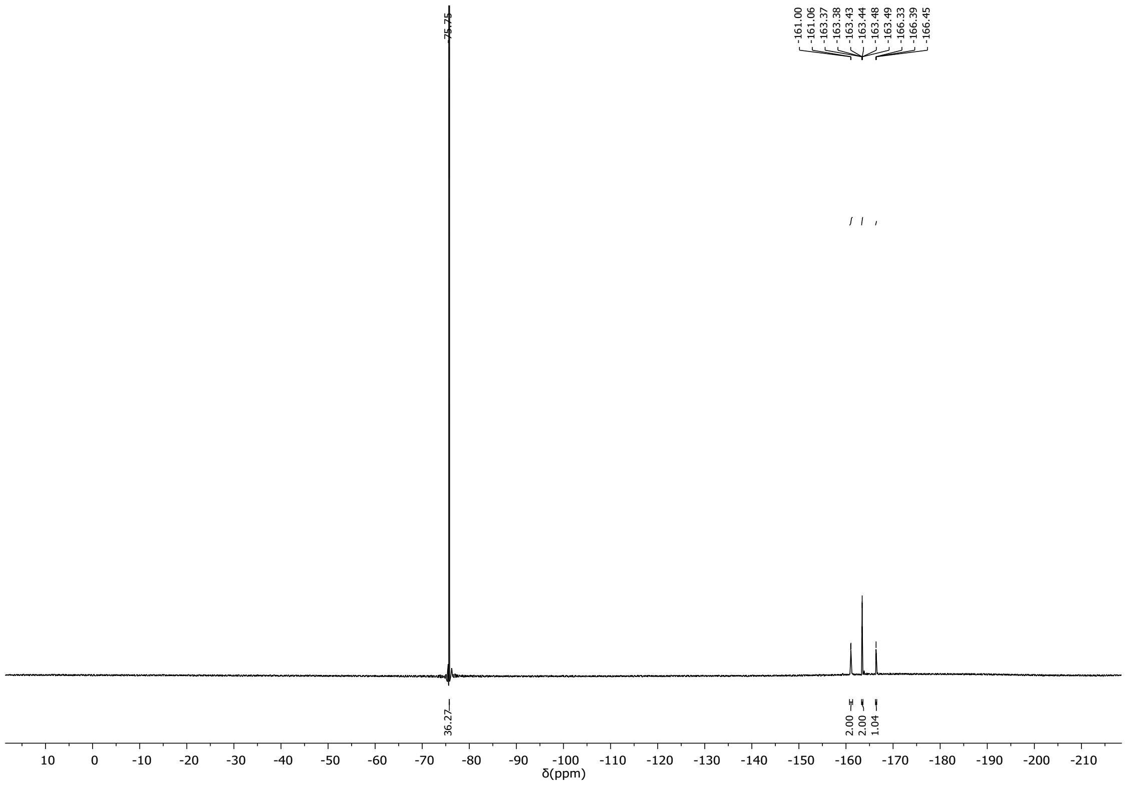

Figure S25. ^{19}F-NMR spectrum (375 MHz, CD_2Cl_2, 25 °C) of

[MoO(CHCMe$_3$)(OC$_6$F$_5$)(IMes)(THF)][Al(OC$_4$F$_9$)$_4$] (**Mo-8**).

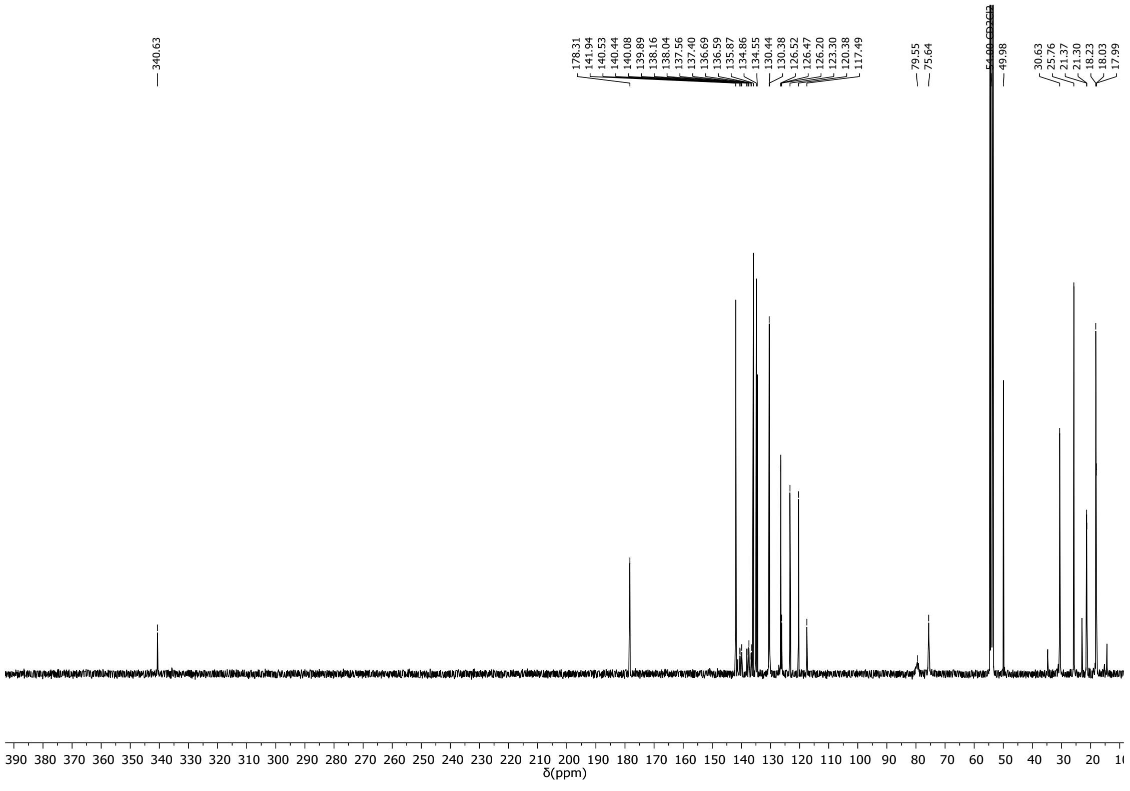

Figure S26. ^{13}C-NMR spectrum (400 MHz, CD$_2$Cl$_2$, 25 °C) of [MoO(CHCMe$_3$)(OC$_6$F$_5$)(IMes)(THF)][Al(OC$_4$F$_9$)$_4$] (**Mo-8**).

4. Single Crystal X-Ray Crystallography Data

Table S4. Crystal data and structure refinement for **Mo-2**.

Empirical formula	$C_{37}H_{30}Cl_2F_{18}MoN_2O_4$
Formula weight	1075.47
Temperature	135(2) K
Wavelength	0.71073 Å
Crystal system, space group	Triclinic, $P\bar{1}$
Unit cell dimensions	a = 11.8411(6) Å, α = 80.252(2)°<br>b = 12.4718(7) Å, β = 81.208(2)°<br>c = 15.3713(8) Å, γ = 66.112(3)°
Volume	2036.64(19) A^3

Z, Calculated density	2, 1.754 Mg/m^3
Absorption coefficient	0.579 mm^{-1}
F(000)	1072
Crystal size	0.323 x 0.200 x 0.191 mm
Theta range for data collection	1.799 to 28.294°
Limiting indices	-15<=h<=15, -16<=k<=16, -19<=l<=20
Reflections collected / unique	43911 / 10061 [R(int) = 0.0211]
Completeness to Θ = 25.242	99.90%
Absorption correction	Semi-empirical from equivalents
Max. and min. transmission	0.7457 and 0.6826
Refinement method	Full-matrix least-squares on F^2
Data / restraints / parameters	10061 / 0 / 588
Goodness-of-fit on F^2	1.024
Final R indices [I>2 σ(I)]	R1 = 0.0528, wR2 = 0.1401
R indices (all data)	R1 = 0.0602, wR2 = 0.1443
Extinction coefficient	n/a
Largest diff. peak and hole	2.354 and -0.842 e.A^{-3}

Table S5. Atomic coordinates (x 10^4) and equivalent isotropic displacement parameters (Å^2 x 10^3) for **Mo-2**. U(eq) is defined as one third of the trace of the orthogonalized U_{ij} tensor.

	x	y	z	U(eq)
MO1	1646(1)	4416(1)	2591(1)	18(1)
CL1	3128(1)	8597(1)	1715(1)	32(1)

CL2	5444(1)	6304(1)	2851(1)	32(1)
O(1)	1635(2)	4349(2)	3682(2)	26(1)
F(1)	4373(3)	1141(2)	566(2)	56(1)
N(1)	2309(2)	6810(2)	2051(2)	21(1)
C(1)	2633(3)	5668(3)	2430(2)	19(1)
O(2)	2665(2)	3865(2)	1443(2)	26(1)
N(2)	3743(2)	5393(2)	2747(2)	20(1)
F(2)	2789(3)	1370(2)	1490(2)	53(1)
C(2)	3222(3)	7220(3)	2121(2)	23(1)
O(3)	1148(2)	2999(2)	2649(2)	24(1)
C(3)	4107(3)	6338(3)	2556(2)	23(1)
F(3)	4298(3)	1686(3)	1818(2)	62(1)
O(4)	-4024(2)	8963(2)	4134(2)	37(1)
F(4)	5167(2)	2847(3)	373(2)	53(1)
C(4)	1123(3)	7630(3)	1756(2)	23(1)
F(5)	4097(3)	3063(3)	-670(2)	59(1)
C(5)	304(3)	8380(3)	2361(2)	26(1)
F(6)	3843(3)	4536(2)	6(2)	64(1)
C(6)	-788(3)	9235(3)	2051(3)	33(1)
F(7)	888(2)	3515(3)	831(2)	55(1)
C(7)	-1064(3)	9352(3)	1187(3)	36(1)
F(8)	2121(3)	2464(3)	-167(2)	66(1)
C(8)	-215(4)	8600(3)	611(3)	34(1)
C(9)	898(3)	7725(3)	878(2)	27(1)
F(9)	1665(3)	4336(3)	-282(2)	66(1)
F(10)	-1349(2)	2694(2)	4194(2)	39(1)
C(10)	585(4)	8265(4)	3297(3)	35(1)
F(11)	-1257(2)	4236(2)	3388(2)	51(1)
C(11)	-2275(4)	10268(4)	874(4)	53(1)
F(12)	-89(2)	3428(3)	4435(2)	49(1)
C(12)	1805(4)	6914(4)	251(2)	35(1)
F(13)	581(3)	1461(3)	1892(2)	50(1)
C(13)	4468(3)	4304(3)	3251(2)	22(1)
F(14)	-649(3)	1251(3)	2994(2)	62(1)
C(14)	4211(3)	4185(3)	4169(2)	25(1)
C(15)	4978(4)	3143(3)	4636(2)	32(1)
F(15)	-1094(3)	2920(3)	2177(2)	74(1)
F(16)	2027(2)	498(2)	3190(2)	47(1)
C(16)	5965(4)	2284(3)	4219(3)	34(1)
F(17)	2126(3)	1584(3)	4062(2)	64(1)
C(17)	6199(3)	2468(3)	3304(3)	34(1)
F(18)	756(3)	857(3)	4356(2)	74(1)
C(18)	5470(3)	3483(3)	2809(2)	28(1)
C(19)	3216(4)	5135(3)	4656(2)	32(1)
C(20)	6805(4)	1192(4)	4743(3)	49(1)

C(21)	5842(4)	3738(4)	1839(3)	42(1)
C(22)	3040(3)	3143(3)	798(2)	26(1)
C(23)	3634(4)	1813(4)	1167(3)	38(1)
C(24)	4025(4)	3425(4)	104(3)	36(1)
C(25)	1883(5)	3364(4)	289(3)	45(1)
C(26)	446(3)	2450(3)	3134(2)	25(1)
C(27)	-571(3)	3192(3)	3801(3)	32(1)
C(28)	-179(4)	2029(4)	2527(3)	38(1)
C(29)	1334(4)	1309(4)	3700(3)	39(1)
C(30)	82(3)	5680(3)	2357(2)	23(1)
C(31)	-1004(3)	6487(3)	2826(2)	23(1)
C(32)	-1110(3)	6520(3)	3743(2)	27(1)
C(33)	-2140(3)	7334(3)	4157(2)	30(1)
C(34)	-3075(3)	8149(3)	3664(3)	29(1)
C(35)	-3025(3)	8113(3)	2764(2)	28(1)
C(36)	-1993(3)	7277(3)	2353(2)	27(1)
C(37)	-4892(4)	9933(4)	3627(3)	45(1)

Table S6. Bond lengths [Å] and angles [°] for **Mo-2**.

MO1-O(1)	1.664(2)
MO1-C(30)	1.921(3)
MO1-O(2)	2.033(2)
MO1-O(3)	2.062(2)
MO1-C(1)	2.263(3)
CL1-C(2)	1.690(3)
CL2-C(3)	1.695(3)
F(1)-C(23)	1.321(5)
N(1)-C(1)	1.364(4)
N(1)-C(2)	1.393(4)
N(1)-C(4)	1.449(4)
C(1)-N(2)	1.363(4)
O(2)-C(22)	1.354(4)
N(2)-C(3)	1.384(4)
N(2)-C(13)	1.444(4)
F(2)-C(23)	1.325(5)
C(2)-C(3)	1.337(5)
O(3)-C(26)	1.347(4)
F(3)-C(23)	1.315(5)
O(4)-C(34)	1.375(4)
O(4)-C(37)	1.431(5)
F(4)-C(24)	1.341(5)
C(4)-C(9)	1.390(5)
C(4)-C(5)	1.403(5)
F(5)-C(24)	1.322(5)

C(5)-C(6)	1.392(5)
C(5)-C(10)	1.495(5)
F(6)-C(24)	1.298(5)
C(6)-C(7)	1.385(6)
C(6)-H(6)	0.95
F(7)-C(25)	1.301(5)
C(7)-C(8)	1.386(6)
C(7)-C(11)	1.514(5)
F(8)-C(25)	1.334(5)
C(8)-C(9)	1.395(5)
C(8)-H(8)	0.95
C(9)-C(12)	1.497(5)
F(9)-C(25)	1.328(6)
F(10)-C(27)	1.329(4)
C(10)-H(10A)	0.98
C(10)-H(10B)	0.98
C(10)-H(10C)	0.98
F(11)-C(27)	1.334(5)
C(11)-H(11A)	0.98
C(11)-H(11B)	0.98
C(11)-H(11C)	0.98
F(12)-C(27)	1.330(5)
C(12)-H(12A)	0.98
C(12)-H(12B)	0.98
C(12)-H(12C)	0.98
F(13)-C(28)	1.309(5)
C(13)-C(18)	1.387(5)
C(13)-C(14)	1.392(4)
F(14)-C(28)	1.358(5)
C(14)-C(15)	1.399(5)
C(14)-C(19)	1.501(5)
C(15)-C(16)	1.383(6)
C(15)-H(15)	0.95
F(15)-C(28)	1.304(5)
F(16)-C(29)	1.309(5)
C(16)-C(17)	1.391(6)
C(16)-C(20)	1.506(5)
F(17)-C(29)	1.339(5)
C(17)-C(18)	1.384(5)
C(17)-H(17)	0.95
F(18)-C(29)	1.309(5)
C(18)-C(21)	1.507(5)
C(19)-H(19A)	0.98
C(19)-H(19B)	0.98
C(19)-H(19C)	0.98

C(20)-H(20A)	0.98
C(20)-H(20B)	0.98
C(20)-H(20C)	0.98
C(21)-H(21A)	0.98
C(21)-H(21B)	0.98
C(21)-H(21C)	0.98
C(22)-C(23)	1.558(5)
C(22)-C(24)	1.564(5)
C(22)-C(25)	1.585(6)
C(26)-C(28)	1.545(5)
C(26)-C(27)	1.549(5)
C(26)-C(29)	1.593(5)
C(30)-C(31)	1.450(4)
C(30)-H(30)	1.03(4)
C(31)-C(36)	1.398(5)
C(31)-C(32)	1.401(5)
C(32)-C(33)	1.379(5)
C(32)-H(32)	0.95
C(33)-C(34)	1.385(5)
C(33)-H(33)	0.95
C(34)-C(35)	1.383(5)
C(35)-C(36)	1.390(5)
C(35)-H(35)	0.95
C(36)-H(36)	0.95
C(37)-H(37A)	0.98
C(37)-H(37B)	0.98
C(37)-H(37C)	0.98
O(1)-MO1-C(30)	102.28(13)
O(1)-MO1-O(2)	146.29(11)
C(30)-MO1-O(2)	110.88(12)
O(1)-MO1-O(3)	95.40(10)
C(30)-MO1-O(3)	99.27(11)
O(2)-MO1-O(3)	85.36(9)
O(1)-MO1-C(1)	87.88(11)
C(30)-MO1-C(1)	92.56(12)
O(2)-MO1-C(1)	84.81(10)
O(3)-MO1-C(1)	166.73(10)
C(1)-N(1)-C(2)	110.6(3)
C(1)-N(1)-C(4)	128.5(3)
C(2)-N(1)-C(4)	119.9(3)
N(1)-C(1)-N(2)	104.2(3)
N(1)-C(1)-MO1	131.6(2)
N(2)-C(1)-MO1	124.1(2)
C(22)-O(2)-MO1	151.6(2)

C(1)-N(2)-C(3)	111.0(3)
C(1)-N(2)-C(13)	127.3(3)
C(3)-N(2)-C(13)	121.7(3)
C(3)-C(2)-N(1)	107.0(3)
C(3)-C(2)-CL1	129.7(3)
N(1)-C(2)-CL1	123.3(3)
C(26)-O(3)-MO1	142.8(2)
C(2)-C(3)-N(2)	107.2(3)
C(2)-C(3)-CL2	129.2(3)
N(2)-C(3)-CL2	123.6(3)
C(34)-O(4)-C(37)	116.6(3)
C(9)-C(4)-C(5)	123.1(3)
C(9)-C(4)-N(1)	119.6(3)
C(5)-C(4)-N(1)	116.9(3)
C(6)-C(5)-C(4)	116.7(3)
C(6)-C(5)-C(10)	121.5(3)
C(4)-C(5)-C(10)	121.7(3)
C(7)-C(6)-C(5)	122.3(3)
C(7)-C(6)-H(6)	118.9
C(5)-C(6)-H(6)	118.9
C(6)-C(7)-C(8)	118.8(3)
C(6)-C(7)-C(11)	121.1(4)
C(8)-C(7)-C(11)	120.1(4)
C(7)-C(8)-C(9)	121.9(4)
C(7)-C(8)-H(8)	119
C(9)-C(8)-H(8)	119
C(4)-C(9)-C(8)	117.2(3)
C(4)-C(9)-C(12)	121.4(3)
C(8)-C(9)-C(12)	121.4(3)
C(5)-C(10)-H(10A)	109.5
C(5)-C(10)-H(10B)	109.5
H(10A)-C(10)-H(10B)	109.5
C(5)-C(10)-H(10C)	109.5
H(10A)-C(10)-H(10C)	109.5
H(10B)-C(10)-H(10C)	109.5
C(7)-C(11)-H(11A)	109.5
C(7)-C(11)-H(11B)	109.5
H(11A)-C(11)-H(11B)	109.5
C(7)-C(11)-H(11C)	109.5
H(11A)-C(11)-H(11C)	109.5
H(11B)-C(11)-H(11C)	109.5
C(9)-C(12)-H(12A)	109.5
C(9)-C(12)-H(12B)	109.5
H(12A)-C(12)-H(12B)	109.5
C(9)-C(12)-H(12C)	109.5

H(12A)-C(12)-H(12C)	109.5
H(12B)-C(12)-H(12C)	109.5
C(18)-C(13)-C(14)	123.0(3)
C(18)-C(13)-N(2)	118.3(3)
C(14)-C(13)-N(2)	118.4(3)
C(13)-C(14)-C(15)	116.5(3)
C(13)-C(14)-C(19)	123.0(3)
C(15)-C(14)-C(19)	120.4(3)
C(16)-C(15)-C(14)	122.3(3)
C(16)-C(15)-H(15)	118.8
C(14)-C(15)-H(15)	118.8
C(15)-C(16)-C(17)	118.6(3)
C(15)-C(16)-C(20)	121.2(4)
C(17)-C(16)-C(20)	120.2(4)
C(18)-C(17)-C(16)	121.5(3)
C(18)-C(17)-H(17)	119.3
C(16)-C(17)-H(17)	119.3
C(17)-C(18)-C(13)	118.0(3)
C(17)-C(18)-C(21)	120.4(3)
C(13)-C(18)-C(21)	121.3(3)
C(14)-C(19)-H(19A)	109.5
C(14)-C(19)-H(19B)	109.5
H(19A)-C(19)-H(19B)	109.5
C(14)-C(19)-H(19C)	109.5
H(19A)-C(19)-H(19C)	109.5
H(19B)-C(19)-H(19C)	109.5
C(16)-C(20)-H(20A)	109.5
C(16)-C(20)-H(20B)	109.5
H(20A)-C(20)-H(20B)	109.5
C(16)-C(20)-H(20C)	109.5
H(20A)-C(20)-H(20C)	109.5
H(20B)-C(20)-H(20C)	109.5
C(18)-C(21)-H(21A)	109.5
C(18)-C(21)-H(21B)	109.5
H(21A)-C(21)-H(21B)	109.5
C(18)-C(21)-H(21C)	109.5
H(21A)-C(21)-H(21C)	109.5
H(21B)-C(21)-H(21C)	109.5
O(2)-C(22)-C(23)	112.7(3)
O(2)-C(22)-C(24)	110.8(3)
C(23)-C(22)-C(24)	107.5(3)
O(2)-C(22)-C(25)	108.9(3)
C(23)-C(22)-C(25)	109.5(3)
C(24)-C(22)-C(25)	107.4(3)
F(3)-C(23)-F(1)	107.5(4)

F(3)-C(23)-F(2)	107.2(4)
F(1)-C(23)-F(2)	106.7(3)
F(3)-C(23)-C(22)	109.7(3)
F(1)-C(23)-C(22)	113.2(3)
F(2)-C(23)-C(22)	112.3(3)
F(6)-C(24)-F(5)	110.7(4)
F(6)-C(24)-F(4)	105.4(4)
F(5)-C(24)-F(4)	104.7(3)
F(6)-C(24)-C(22)	112.2(3)
F(5)-C(24)-C(22)	112.6(3)
F(4)-C(24)-C(22)	110.7(3)
F(7)-C(25)-F(9)	107.9(4)
F(7)-C(25)-F(8)	109.8(4)
F(9)-C(25)-F(8)	108.0(4)
F(7)-C(25)-C(22)	111.6(3)
F(9)-C(25)-C(22)	109.1(4)
F(8)-C(25)-C(22)	110.4(4)
O(3)-C(26)-C(28)	110.8(3)
O(3)-C(26)-C(27)	115.1(3)
C(28)-C(26)-C(27)	108.3(3)
O(3)-C(26)-C(29)	108.1(3)
C(28)-C(26)-C(29)	107.6(3)
C(27)-C(26)-C(29)	106.7(3)
F(12)-C(27)-F(10)	107.4(3)
F(12)-C(27)-F(11)	105.9(3)
F(10)-C(27)-F(11)	106.8(3)
F(12)-C(27)-C(26)	111.9(3)
F(10)-C(27)-C(26)	114.2(3)
F(11)-C(27)-C(26)	110.1(3)
F(15)-C(28)-F(13)	108.9(4)
F(15)-C(28)-F(14)	107.7(4)
F(13)-C(28)-F(14)	104.7(3)
F(15)-C(28)-C(26)	110.5(3)
F(13)-C(28)-C(26)	113.8(3)
F(14)-C(28)-C(26)	110.9(3)
F(18)-C(29)-F(16)	109.7(4)
F(18)-C(29)-F(17)	106.3(4)
F(16)-C(29)-F(17)	105.5(3)
F(18)-C(29)-C(26)	114.4(3)
F(16)-C(29)-C(26)	110.7(3)
F(17)-C(29)-C(26)	109.7(3)
C(31)-C(30)-MO1	139.9(3)
C(31)-C(30)-H(30)	111(2)
MO1-C(30)-H(30)	109(2)
C(36)-C(31)-C(32)	117.8(3)

C(36)-C(31)-C(30)	119.3(3)
C(32)-C(31)-C(30)	122.9(3)
C(33)-C(32)-C(31)	120.9(3)
C(33)-C(32)-H(32)	119.6
C(31)-C(32)-H(32)	119.6
C(32)-C(33)-C(34)	120.1(3)
C(32)-C(33)-H(33)	120
C(34)-C(33)-H(33)	120
O(4)-C(34)-C(35)	124.1(3)
O(4)-C(34)-C(33)	115.2(3)
C(35)-C(34)-C(33)	120.6(3)
C(34)-C(35)-C(36)	118.9(3)
C(34)-C(35)-H(35)	120.5
C(36)-C(35)-H(35)	120.5
C(35)-C(36)-C(31)	121.5(3)
C(35)-C(36)-H(36)	119.2
C(31)-C(36)-H(36)	119.2
O(4)-C(37)-H(37A)	109.5
O(4)-C(37)-H(37B)	109.5
H(37A)-C(37)-H(37B)	109.5
O(4)-C(37)-H(37C)	109.5
H(37A)-C(37)-H(37C)	109.5
H(37B)-C(37)-H(37C)	109.5

Table S7. Anisotropic displacement parameters ($Å^2 \times 10^3$) for **Mo-2**. The anisotropic displacement factor exponent takes the form: $-2 \pi^2 [h^2 a^{*2} U_{11} + ... + 2 h k a^* b^* U_{12}]$.

	U11	U22	U33	U23	U13	U12
MO1	20(1)	18(1)	18(1)	-4(1)	1(1)	-9(1)
CL1	33(1)	20(1)	44(1)	-2(1)	0(1)	-15(1)
CL2	31(1)	42(1)	33(1)	-1(1)	-7(1)	-23(1)
O(1)	32(1)	27(1)	24(1)	-2(1)	-3(1)	-16(1)
F(1)	54(2)	31(1)	71(2)	-20(1)	24(1)	-9(1)
N(1)	22(1)	20(1)	23(1)	-4(1)	-1(1)	-10(1)
C(1)	21(1)	21(1)	16(1)	-4(1)	0(1)	-9(1)
O(2)	36(1)	24(1)	20(1)	-8(1)	6(1)	-14(1)
N(2)	22(1)	21(1)	17(1)	-1(1)	-1(1)	-10(1)
F(2)	61(2)	32(1)	62(2)	-13(1)	20(1)	-22(1)
C(2)	26(2)	20(1)	26(2)	-5(1)	1(1)	-12(1)

O(3)	27(1)	22(1)	27(1)	-7(1)	5(1)	-14(1)
C(3)	26(2)	28(2)	21(1)	-5(1)	0(1)	-17(1)
F(3)	70(2)	45(2)	62(2)	6(1)	-32(2)	-9(1)
O(4)	29(1)	27(1)	44(2)	-4(1)	4(1)	-2(1)
F(4)	37(1)	60(2)	61(2)	-15(1)	10(1)	-19(1)
C(4)	21(1)	18(1)	31(2)	-2(1)	-1(1)	-9(1)
F(5)	72(2)	70(2)	31(1)	-19(1)	19(1)	-27(2)
C(5)	26(2)	19(1)	37(2)	-6(1)	1(1)	-11(1)
F(6)	78(2)	40(1)	64(2)	-10(1)	41(2)	-29(1)
C(6)	23(2)	19(2)	56(2)	-8(2)	1(2)	-8(1)
F(7)	35(1)	76(2)	53(2)	-5(1)	-12(1)	-18(1)
C(7)	26(2)	21(2)	57(2)	8(2)	-9(2)	-8(1)
F(8)	87(2)	84(2)	47(2)	-24(2)	-17(2)	-42(2)
C(8)	33(2)	30(2)	38(2)	9(2)	-12(2)	-14(2)
C(9)	29(2)	22(2)	31(2)	1(1)	-2(1)	-12(1)
F(9)	62(2)	73(2)	42(2)	12(1)	-17(1)	-8(2)
F(10)	38(1)	34(1)	49(1)	-12(1)	16(1)	-22(1)
C(10)	33(2)	36(2)	39(2)	-17(2)	5(2)	-14(2)
F(11)	44(1)	26(1)	66(2)	-4(1)	17(1)	-6(1)
C(11)	34(2)	34(2)	78(4)	11(2)	-17(2)	-3(2)
F(12)	45(1)	65(2)	51(2)	-36(1)	16(1)	-32(1)
C(12)	39(2)	35(2)	28(2)	-3(1)	-2(2)	-12(2)
F(13)	53(2)	65(2)	46(1)	-31(1)	6(1)	-33(1)
C(13)	23(1)	23(1)	21(1)	0(1)	-4(1)	-11(1)
F(14)	70(2)	67(2)	74(2)	-30(2)	19(2)	-53(2)
C(14)	28(2)	29(2)	21(2)	-1(1)	-2(1)	-15(1)
C(15)	36(2)	38(2)	25(2)	8(1)	-7(1)	-21(2)
F(15)	67(2)	63(2)	85(2)	-17(2)	-46(2)	-2(2)
F(16)	44(1)	32(1)	55(2)	-11(1)	0(1)	-3(1)
C(16)	31(2)	31(2)	42(2)	11(2)	-12(2)	-15(2)
F(17)	62(2)	56(2)	69(2)	-12(2)	-34(2)	-5(1)
C(17)	27(2)	28(2)	41(2)	-2(2)	-1(2)	-7(1)
F(18)	54(2)	61(2)	72(2)	29(2)	9(2)	-6(2)
C(18)	27(2)	29(2)	26(2)	-3(1)	-1(1)	-9(1)
C(19)	36(2)	39(2)	23(2)	-10(1)	3(1)	-15(2)
C(20)	41(2)	39(2)	60(3)	21(2)	-16(2)	-16(2)
C(21)	42(2)	44(2)	25(2)	-5(2)	4(2)	-2(2)
C(22)	29(2)	25(2)	21(2)	-6(1)	2(1)	-9(1)
C(23)	41(2)	29(2)	38(2)	-6(2)	4(2)	-7(2)
C(24)	40(2)	39(2)	27(2)	-9(2)	9(2)	-16(2)
C(25)	55(3)	57(3)	24(2)	-9(2)	-4(2)	-23(2)
C(26)	27(2)	25(2)	29(2)	-8(1)	3(1)	-15(1)
C(27)	32(2)	30(2)	37(2)	-9(1)	8(1)	-17(2)
C(28)	36(2)	42(2)	45(2)	-13(2)	-1(2)	-21(2)
C(29)	37(2)	30(2)	43(2)	-1(2)	2(2)	-10(2)

C(30)	22(1)	21(1)	26(2)	-2(1)	-4(1)	-10(1)
C(31)	22(2)	20(1)	29(2)	-3(1)	-2(1)	-10(1)
C(32)	27(2)	24(2)	28(2)	-1(1)	-5(1)	-9(1)
C(33)	32(2)	28(2)	29(2)	-6(1)	0(1)	-10(1)
C(34)	23(2)	21(2)	40(2)	-3(1)	2(1)	-8(1)
C(35)	21(2)	24(2)	38(2)	2(1)	-7(1)	-8(1)
C(36)	26(2)	26(2)	30(2)	0(1)	-5(1)	-12(1)
C(37)	29(2)	30(2)	61(3)	0(2)	0(2)	0(2)

Table S8. Crystal data and structure refinement for **Mo-8**.

Empirical formula	$C_{53}H_{44}AlClF_{41}MoN_2O_7$
Formula weight	1758.27
Temperature	140(2) K
Wavelength	0.71073 A
Crystal system, space group	Triclinic, $P\bar{1}$
Unit cell dimensions	a = 12.9654(8) Å, α = 79.898(2)°<br>b = 13.8876(9) Å, β = 78.994(2)°<br>c = 19.3009(12) Å, γ = 86.057(3)°
Volume	3356.2(4) A^3
Z, Calculated density	2, 1.740 Mg/m^3
Absorption coefficient	0.412 mm^{-1}
F(000)	1746
Crystal size	0.490 x 0.421 x 0.343 mm
Theta range for data collection	1.490 to 28.403 °.
Limiting indices	-17<=h<=17, -18<=k<=18, -25<=l<=25
Reflections collected / unique	71115 / 16752 [R(int) = 0.0287]
Completeness to θ = 25.242	99.90%
Absorption correction	Semi-empirical from equivalents

Max. and min. transmission	0.7457 and 0.7096
Refinement method	Full-matrix least-squares on F^2
Data / restraints / parameters	16752 / 66 / 1007
Goodness-of-fit on F^2	1.041
Final R indices [I>2σ(I)]	R1 = 0.0718, wR2 = 0.1740
R indices (all data)	R1 = 0.0914, wR2 = 0.1845
Extinction coefficient	n/a
Largest diff. peak and hole	1.794 and -0.978 e.A^{-3}

Table S9. Atomic coordinates (x 10^4) and equivalent isotropic displacement parameters ($Å^2$ x 10^3) for **Mo-8**. U(eq) is defined as one third of the trace of the orthogonalized U_{ij} tensor.

	x	y	z	U(eq)
Mo(1)	7995(1)	6755(1)	7501(1)	20(1)
O(1)	6718(2)	7047(2)	7698(1)	30(1)
N(1)	7081(2)	4977(2)	7023(2)	26(1)
C(1)	7795(3)	5687(2)	6822(2)	22(1)
F(1)	7677(3)	5374(2)	8938(2)	58(1)
O(2)	9196(2)	5980(2)	7810(1)	32(1)
N(2)	8305(3)	5537(2)	6165(2)	28(1)
C(2)	7150(4)	4394(3)	6505(2)	38(1)
F(2)	8118(5)	4141(3)	10099(2)	114(2)
O(3)	8145(2)	7464(2)	8412(1)	28(1)
C(3)	7916(4)	4743(3)	5968(2)	42(1)
F(3)	10126(6)	3517(3)	10150(2)	140(3)
C(4)	6349(3)	4801(3)	7693(2)	25(1)
F(4)	11680(4)	4169(4)	9062(3)	127(2)
C(5)	6624(3)	4081(3)	8233(2)	33(1)
F(5)	11243(3)	5416(3)	7894(2)	75(1)
C(6)	5892(4)	3903(4)	8865(2)	47(1)
C(7)	4938(4)	4404(4)	8957(3)	51(1)

C(8)	4685(3)	5096(4)	8402(2)	42(1)
C(9)	5383(3)	5305(3)	7755(2)	29(1)
C(10)	7647(4)	3506(3)	8130(3)	44(1)
C(11)	4149(5)	4179(7)	9650(3)	89(2)
C(12)	5059(4)	6028(3)	7153(2)	40(1)
C(13)	9120(3)	6115(3)	5697(2)	27(1)
C(14)	10139(3)	5981(3)	5815(2)	33(1)
C(15)	10883(3)	6584(3)	5353(2)	38(1)
C(16)	10633(4)	7244(3)	4782(2)	42(1)
C(17)	9615(4)	7307(3)	4660(2)	41(1)
C(18)	8828(3)	6763(3)	5120(2)	33(1)
C(19)	10461(4)	5184(4)	6377(2)	52(1)
C(20)	11476(5)	7870(4)	4286(3)	66(2)
C(21)	7726(4)	6861(4)	4981(3)	48(1)
C(22)	9437(4)	5404(3)	8385(2)	39(1)
C(23)	8667(5)	5079(3)	8963(3)	50(1)
C(24)	8894(7)	4444(4)	9556(3)	76(2)
C(25)	9893(9)	4137(4)	9579(4)	87(3)
C(26)	10708(7)	4468(5)	9025(4)	84(3)
C(27)	10480(5)	5099(4)	8420(3)	56(2)
C(28)	8536(3)	7783(2)	6770(2)	23(1)
C(29)	8161(3)	8776(3)	6439(2)	30(1)
C(30)	8878(5)	9126(3)	5721(2)	49(1)
C(31)	8261(4)	9467(3)	6958(2)	42(1)
C(32)	7027(4)	8781(4)	6339(3)	54(1)
C(33)	9087(3)	7882(3)	8527(2)	32(1)
C(34)	8699(4)	8492(4)	9110(3)	50(1)
C(35)	7692(5)	8032(5)	9498(3)	57(1)
C(36)	7231(4)	7768(4)	8903(2)	41(1)
Al(1)	2920(1)	10309(1)	7682(1)	21(1)
O(4)	2613(14)	9820(20)	6986(15)	43(5)
O(4A)	2690(20)	9510(20)	7174(13)	57(5)
O(5)	1897(3)	11080(3)	7930(2)	60(1)
F(6)	4158(3)	8132(3)	6010(2)	88(1)
O(6)	4123(6)	10824(6)	7549(7)	43(3)
O(6A)	3864(5)	11068(5)	7135(4)	44(2)
F(7)	4453(3)	9640(3)	5917(2)	82(1)
O(7)	2881(9)	9336(7)	8391(4)	35(3)
O(7A)	3409(6)	9714(5)	8387(3)	50(2)
F(8)	4418(3)	8682(4)	6921(2)	89(1)
F(9)	1358(4)	9693(4)	5992(3)	110(2)
F(10)	2647(5)	10615(3)	5795(3)	127(2)
F(11)	2746(5)	9291(4)	5320(2)	114(2)
F(12)	2087(4)	7704(3)	6315(3)	100(2)
F(13)	1256(3)	8333(4)	7194(3)	108(2)

F(14)	2693(4)	7541(3)	7260(2)	94(1)
F(15)	1574(3)	12456(3)	6865(2)	65(1)
F(16)	1421(3)	13156(2)	7785(2)	69(1)
F(17)	59(3)	12861(3)	7375(3)	92(1)
F(18)	314(3)	9946(2)	7999(2)	70(1)
F(19)	363(3)	10960(3)	7018(2)	74(1)
F(20)	-814(3)	11115(3)	7967(3)	88(1)
F(21)	-287(3)	12301(3)	8801(2)	90(1)
F(22)	1243(4)	11981(4)	9029(2)	102(2)
F(23)	219(4)	10776(3)	9161(2)	88(1)
F(24)	5599(4)	12698(3)	6131(3)	95(1)
F(25)	5738(3)	11152(3)	6113(3)	92(1)
F(26)	4316(4)	11939(5)	5971(4)	149(3)
F(27)	3664(3)	12411(3)	8064(4)	126(2)
F(28)	4963(3)	13200(3)	7433(3)	108(2)
F(29)	3612(4)	13079(4)	6971(4)	138(3)
F(30)	5870(5)	10374(4)	7466(3)	138(2)
F(31)	5387(5)	11350(5)	8191(3)	135(2)
F(32)	6482(3)	11810(4)	7217(2)	94(1)
F(33)	5030(4)	9365(5)	9104(4)	130(2)
F(34)	3725(6)	10373(4)	9425(4)	151(3)
F(35)	3777(7)	9041(5)	10106(3)	177(3)
F(36)	4683(4)	8200(4)	8317(3)	114(2)
C(37)	2781(3)	9123(3)	6575(2)	36(1)
F(37)	4238(5)	7549(4)	9381(3)	137(2)
C(38)	3980(5)	8868(4)	6337(3)	58(1)
F(38)	3171(6)	7506(4)	8618(4)	143(2)
C(39)	2371(6)	9699(5)	5910(4)	75(2)
F(39)	1905(4)	9629(5)	9759(3)	132(2)
C(40)	2185(5)	8169(4)	6846(3)	58(1)
F(40)	2258(5)	7952(5)	9940(3)	138(2)
C(41)	941(3)	11493(3)	7982(2)	31(1)
F(41)	1575(4)	8652(6)	9042(5)	182(3)
C(42)	1003(4)	12511(3)	7497(3)	46(1)
C(43)	169(4)	10888(4)	7742(3)	54(1)
C(44)	538(6)	11636(5)	8761(3)	65(2)
C(45)	4679(4)	11659(4)	7147(4)	69(2)
C(46)	5093(5)	11851(5)	6333(5)	82(2)
C(47)	4224(5)	12607(5)	7404(6)	94(3)
C(48)	5618(6)	11283(6)	7511(5)	85(2)
C(49)	3292(5)	8983(4)	8998(2)	52(1)
C(50)	4047(8)	9454(7)	9417(5)	104(3)
C(51)	3893(7)	8058(6)	8815(4)	83(2)
C(52)	2242(9)	8784(9)	9446(6)	116(3)
C(1X)	5488(14)	5206(17)	4913(13)	107(5)

C(1Y)	5083(15)	4705(18)	4745(13)	105(5)
Cl(1X)	6424(2)	4506(2)	4402(2)	114(1)

Table S10. Bond lengths [Å] and angles [°] for **Mo-8**.

Mo(1)-O(1)	1.664(3)
Mo(1)-C(28)	1.891(3)
Mo(1)-O(2)	1.958(3)
Mo(1)-C(1)	2.203(3)
Mo(1)-O(3)	2.205(2)
N(1)-C(1)	1.354(4)
N(1)-C(2)	1.379(5)
N(1)-C(4)	1.445(4)
C(1)-N(2)	1.357(4)
F(1)-C(23)	1.328(7)
O(2)-C(22)	1.326(5)
N(2)-C(3)	1.383(5)
N(2)-C(13)	1.440(4)
C(2)-C(3)	1.339(6)
C(2)-H(2)	0.95
F(2)-C(24)	1.337(9)
O(3)-C(36)	1.457(5)
O(3)-C(33)	1.458(5)
C(3)-H(3)	0.95
F(3)-C(25)	1.344(6)
C(4)-C(9)	1.387(5)
C(4)-C(5)	1.392(5)
F(4)-C(26)	1.310(8)
C(5)-C(6)	1.391(6)
C(5)-C(10)	1.498(6)
F(5)-C(27)	1.313(8)
C(6)-C(7)	1.373(7)
C(6)-H(6)	0.95
C(7)-C(8)	1.381(7)
C(7)-C(11)	1.520(7)
C(8)-C(9)	1.393(6)
C(8)-H(8)	0.95
C(9)-C(12)	1.501(5)
C(10)-H(10A)	0.98
C(10)-H(10B)	0.98
C(10)-H(10C)	0.98
C(11)-H(11A)	0.98
C(11)-H(11B)	0.98
C(11)-H(11C)	0.98
C(12)-H(12A)	0.98

C(12)-H(12B)	0.98
C(12)-H(12C)	0.98
C(13)-C(14)	1.378(6)
C(13)-C(18)	1.398(6)
C(14)-C(15)	1.396(6)
C(14)-C(19)	1.504(6)
C(15)-C(16)	1.380(7)
C(15)-H(15)	0.95
C(16)-C(17)	1.378(7)
C(16)-C(20)	1.519(6)
C(17)-C(18)	1.387(6)
C(17)-H(17)	0.95
C(18)-C(21)	1.496(7)
C(19)-H(19A)	0.98
C(19)-H(19B)	0.98
C(19)-H(19C)	0.98
C(20)-H(20A)	0.98
C(20)-H(20B)	0.98
C(20)-H(20C)	0.98
C(21)-H(21A)	0.98
C(21)-H(21B)	0.98
C(21)-H(21C)	0.98
C(22)-C(23)	1.380(8)
C(22)-C(27)	1.400(7)
C(23)-C(24)	1.383(7)
C(24)-C(25)	1.343(12)
C(25)-C(26)	1.390(12)
C(26)-C(27)	1.400(8)
C(28)-C(29)	1.504(5)
C(28)-H(28)	0.98(4)
C(29)-C(32)	1.519(6)
C(29)-C(31)	1.531(5)
C(29)-C(30)	1.534(6)
C(30)-H(30A)	0.98
C(30)-H(30B)	0.98
C(30)-H(30C)	0.98
C(31)-H(31A)	0.98
C(31)-H(31B)	0.98
C(31)-H(31C)	0.98
C(32)-H(32A)	0.98
C(32)-H(32B)	0.98
C(32)-H(32C)	0.98
C(33)-C(34)	1.515(6)
C(33)-H(33A)	0.99
C(33)-H(33B)	0.99

C(34)-C(35)	1.500(7)
C(34)-H(34A)	0.99
C(34)-H(34B)	0.99
C(35)-C(36)	1.501(6)
C(35)-H(35A)	0.99
C(35)-H(35B)	0.99
C(36)-H(36A)	0.99
C(36)-H(36B)	0.99
Al(1)-O(7A)	1.674(5)
Al(1)-O(4A)	1.679(11)
Al(1)-O(5)	1.696(3)
Al(1)-O(6)	1.717(7)
Al(1)-O(4)	1.724(12)
Al(1)-O(6A)	1.737(6)
Al(1)-O(7)	1.743(7)
O(4)-C(37)	1.343(14)
O(4A)-C(37)	1.342(13)
O(5)-C(41)	1.322(5)
F(6)-C(38)	1.278(6)
O(6)-C(45)	1.432(10)
O(6A)-C(45)	1.388(7)
F(7)-C(38)	1.334(7)
O(7)-C(49)	1.377(9)
O(7A)-C(49)	1.406(8)
F(8)-C(38)	1.335(7)
F(9)-C(39)	1.294(8)
F(10)-C(39)	1.315(8)
F(11)-C(39)	1.354(9)
F(12)-C(40)	1.332(7)
F(13)-C(40)	1.288(7)
F(14)-C(40)	1.309(7)
F(15)-C(42)	1.313(6)
F(16)-C(42)	1.326(6)
F(17)-C(42)	1.334(6)
F(18)-C(43)	1.329(6)
F(19)-C(43)	1.358(7)
F(20)-C(43)	1.302(7)
F(21)-C(44)	1.364(8)
F(22)-C(44)	1.289(8)
F(23)-C(44)	1.348(7)
F(24)-C(46)	1.347(8)
F(25)-C(46)	1.311(8)
F(26)-C(46)	1.319(9)
F(27)-C(47)	1.332(11)
F(28)-C(47)	1.320(7)

F(29)-C(47)	1.322(11)
F(30)-C(48)	1.296(9)
F(31)-C(48)	1.307(9)
F(32)-C(48)	1.355(8)
F(33)-C(50)	1.310(10)
F(34)-C(50)	1.317(10)
F(35)-C(50)	1.342(11)
F(36)-C(51)	1.265(9)
C(37)-C(40)	1.539(7)
C(37)-C(39)	1.552(7)
C(37)-C(38)	1.569(7)
F(37)-C(51)	1.328(8)
F(38)-C(51)	1.395(10)
F(39)-C(52)	1.418(12)
F(40)-C(52)	1.364(12)
C(41)-C(43)	1.535(6)
C(41)-C(44)	1.537(6)
C(41)-C(42)	1.551(6)
F(41)-C(52)	1.311(12)
C(45)-C(47)	1.530(8)
C(45)-C(48)	1.535(11)
C(45)-C(46)	1.543(11)
C(49)-C(52)	1.482(12)
C(49)-C(51)	1.516(9)
C(49)-C(50)	1.617(11)
C(1X)-C(1X)#1	1.38(4)
C(1X)-Cl(1X)	1.76(2)
C(1X)-H(1X1)	0.99
C(1X)-H(1X2)	0.99
C(1Y)-C(1Y)#1	1.37(4)
C(1Y)-Cl(1X)	1.76(2)
C(1Y)-H(1Y1)	0.99
C(1Y)-H(1Y2)	0.99
O(1)-Mo(1)-C(28)	104.19(15)
O(1)-Mo(1)-O(2)	146.73(13)
C(28)-Mo(1)-O(2)	107.41(14)
O(1)-Mo(1)-C(1)	94.78(13)
C(28)-Mo(1)-C(1)	98.43(13)
O(2)-Mo(1)-C(1)	90.70(12)
O(1)-Mo(1)-O(3)	85.50(11)
C(28)-Mo(1)-O(3)	96.81(12)
O(2)-Mo(1)-O(3)	80.74(11)
C(1)-Mo(1)-O(3)	164.20(11)
C(1)-N(1)-C(2)	111.1(3)

C(1)-N(1)-C(4)	126.5(3)
C(2)-N(1)-C(4)	122.4(3)
N(1)-C(1)-N(2)	104.3(3)
N(1)-C(1)-Mo(1)	123.4(2)
N(2)-C(1)-Mo(1)	132.3(2)
C(22)-O(2)-Mo(1)	139.4(3)
C(1)-N(2)-C(3)	110.8(3)
C(1)-N(2)-C(13)	127.3(3)
C(3)-N(2)-C(13)	121.9(3)
C(3)-C(2)-N(1)	106.9(3)
C(3)-C(2)-H(2)	126.5
N(1)-C(2)-H(2)	126.5
C(36)-O(3)-C(33)	109.8(3)
C(36)-O(3)-Mo(1)	122.1(2)
C(33)-O(3)-Mo(1)	126.8(2)
C(2)-C(3)-N(2)	106.9(3)
C(2)-C(3)-H(3)	126.6
N(2)-C(3)-H(3)	126.6
C(9)-C(4)-C(5)	123.1(3)
C(9)-C(4)-N(1)	118.9(3)
C(5)-C(4)-N(1)	117.8(3)
C(6)-C(5)-C(4)	116.8(4)
C(6)-C(5)-C(10)	121.6(4)
C(4)-C(5)-C(10)	121.6(4)
C(7)-C(6)-C(5)	122.1(4)
C(7)-C(6)-H(6)	119
C(5)-C(6)-H(6)	119
C(6)-C(7)-C(8)	119.3(4)
C(6)-C(7)-C(11)	120.7(5)
C(8)-C(7)-C(11)	119.9(5)
C(7)-C(8)-C(9)	121.3(4)
C(7)-C(8)-H(8)	119.3
C(9)-C(8)-H(8)	119.3
C(4)-C(9)-C(8)	117.3(4)
C(4)-C(9)-C(12)	122.9(4)
C(8)-C(9)-C(12)	119.7(4)
C(5)-C(10)-H(10A)	109.5
C(5)-C(10)-H(10B)	109.5
H(10A)-C(10)-H(10B)	109.5
C(5)-C(10)-H(10C)	109.5
H(10A)-C(10)-H(10C)	109.5
H(10B)-C(10)-H(10C)	109.5
C(7)-C(11)-H(11A)	109.5
C(7)-C(11)-H(11B)	109.5
H(11A)-C(11)-H(11B)	109.5

C(7)-C(11)-H(11C)	109.5
H(11A)-C(11)-H(11C)	109.5
H(11B)-C(11)-H(11C)	109.5
C(9)-C(12)-H(12A)	109.5
C(9)-C(12)-H(12B)	109.5
H(12A)-C(12)-H(12B)	109.5
C(9)-C(12)-H(12C)	109.5
H(12A)-C(12)-H(12C)	109.5
H(12B)-C(12)-H(12C)	109.5
C(14)-C(13)-C(18)	123.4(3)
C(14)-C(13)-N(2)	119.5(3)
C(18)-C(13)-N(2)	117.0(4)
C(13)-C(14)-C(15)	116.5(4)
C(13)-C(14)-C(19)	122.5(4)
C(15)-C(14)-C(19)	120.9(4)
C(16)-C(15)-C(14)	122.2(4)
C(16)-C(15)-H(15)	118.9
C(14)-C(15)-H(15)	118.9
C(17)-C(16)-C(15)	119.0(4)
C(17)-C(16)-C(20)	120.8(5)
C(15)-C(16)-C(20)	120.2(5)
C(16)-C(17)-C(18)	121.6(4)
C(16)-C(17)-H(17)	119.2
C(18)-C(17)-H(17)	119.2
C(17)-C(18)-C(13)	117.1(4)
C(17)-C(18)-C(21)	120.4(4)
C(13)-C(18)-C(21)	122.5(4)
C(14)-C(19)-H(19A)	109.5
C(14)-C(19)-H(19B)	109.5
H(19A)-C(19)-H(19B)	109.5
C(14)-C(19)-H(19C)	109.5
H(19A)-C(19)-H(19C)	109.5
H(19B)-C(19)-H(19C)	109.5
C(16)-C(20)-H(20A)	109.5
C(16)-C(20)-H(20B)	109.5
H(20A)-C(20)-H(20B)	109.5
C(16)-C(20)-H(20C)	109.5
H(20A)-C(20)-H(20C)	109.5
H(20B)-C(20)-H(20C)	109.5
C(18)-C(21)-H(21A)	109.5
C(18)-C(21)-H(21B)	109.5
H(21A)-C(21)-H(21B)	109.5
C(18)-C(21)-H(21C)	109.5
H(21A)-C(21)-H(21C)	109.5
H(21B)-C(21)-H(21C)	109.5

O(2)-C(22)-C(23)	121.1(4)
O(2)-C(22)-C(27)	120.8(5)
C(23)-C(22)-C(27)	118.1(5)
F(1)-C(23)-C(22)	118.4(4)
F(1)-C(23)-C(24)	119.4(6)
C(22)-C(23)-C(24)	122.1(6)
F(2)-C(24)-C(25)	120.7(6)
F(2)-C(24)-C(23)	119.8(7)
C(25)-C(24)-C(23)	119.5(7)
C(24)-C(25)-F(3)	120.2(9)
C(24)-C(25)-C(26)	121.1(5)
F(3)-C(25)-C(26)	118.7(8)
F(4)-C(26)-C(25)	120.2(7)
F(4)-C(26)-C(27)	120.3(9)
C(25)-C(26)-C(27)	119.5(7)
F(5)-C(27)-C(26)	120.1(6)
F(5)-C(27)-C(22)	120.3(5)
C(26)-C(27)-C(22)	119.6(7)
C(29)-C(28)-Mo(1)	137.3(3)
C(29)-C(28)-H(28)	116(3)
Mo(1)-C(28)-H(28)	105(3)
C(28)-C(29)-C(32)	112.0(3)
C(28)-C(29)-C(31)	105.8(3)
C(32)-C(29)-C(31)	109.9(4)
C(28)-C(29)-C(30)	110.3(4)
C(32)-C(29)-C(30)	110.4(4)
C(31)-C(29)-C(30)	108.3(3)
C(29)-C(30)-H(30A)	109.5
C(29)-C(30)-H(30B)	109.5
H(30A)-C(30)-H(30B)	109.5
C(29)-C(30)-H(30C)	109.5
H(30A)-C(30)-H(30C)	109.5
H(30B)-C(30)-H(30C)	109.5
C(29)-C(31)-H(31A)	109.5
C(29)-C(31)-H(31B)	109.5
H(31A)-C(31)-H(31B)	109.5
C(29)-C(31)-H(31C)	109.5
H(31A)-C(31)-H(31C)	109.5
H(31B)-C(31)-H(31C)	109.5
C(29)-C(32)-H(32A)	109.5
C(29)-C(32)-H(32B)	109.5
H(32A)-C(32)-H(32B)	109.5
C(29)-C(32)-H(32C)	109.5
H(32A)-C(32)-H(32C)	109.5
H(32B)-C(32)-H(32C)	109.5

O(3)-C(33)-C(34)	104.8(3)
O(3)-C(33)-H(33A)	110.8
C(34)-C(33)-H(33A)	110.8
O(3)-C(33)-H(33B)	110.8
C(34)-C(33)-H(33B)	110.8
H(33A)-C(33)-H(33B)	108.9
C(35)-C(34)-C(33)	103.7(4)
C(35)-C(34)-H(34A)	111
C(33)-C(34)-H(34A)	111
C(35)-C(34)-H(34B)	111
C(33)-C(34)-H(34B)	111
H(34A)-C(34)-H(34B)	109
C(36)-C(35)-C(34)	102.6(4)
C(36)-C(35)-H(35A)	111.2
C(34)-C(35)-H(35A)	111.2
C(36)-C(35)-H(35B)	111.2
C(34)-C(35)-H(35B)	111.2
H(35A)-C(35)-H(35B)	109.2
O(3)-C(36)-C(35)	103.7(4)
O(3)-C(36)-H(36A)	111
C(35)-C(36)-H(36A)	111
O(3)-C(36)-H(36B)	111
C(35)-C(36)-H(36B)	111
H(36A)-C(36)-H(36B)	109
O(7A)-Al(1)-O(4A)	109.9(15)
O(7A)-Al(1)-O(5)	111.8(3)
O(4A)-Al(1)-O(5)	115.7(13)
O(5)-Al(1)-O(6)	113.4(3)
O(5)-Al(1)-O(4)	106.1(4)
O(6)-Al(1)-O(4)	117.5(11)
O(7A)-Al(1)-O(6A)	109.2(4)
O(4A)-Al(1)-O(6A)	105.2(6)
O(5)-Al(1)-O(6A)	104.5(3)
O(5)-Al(1)-O(7)	106.0(3)
O(6)-Al(1)-O(7)	107.5(6)
O(4)-Al(1)-O(7)	105.5(13)
C(37)-O(4)-Al(1)	148.8(14)
C(37)-O(4A)-Al(1)	156.0(13)
C(41)-O(5)-Al(1)	158.8(3)
C(45)-O(6)-Al(1)	140.5(9)
C(45)-O(6A)-Al(1)	143.0(7)
C(49)-O(7)-Al(1)	143.3(9)
C(49)-O(7A)-Al(1)	148.3(7)
O(4A)-C(37)-C(40)	102.2(9)
O(4)-C(37)-C(40)	118.1(17)

O(4A)-C(37)-C(39)	120.7(19)
O(4)-C(37)-C(39)	98.5(13)
C(40)-C(37)-C(39)	109.7(4)
O(4A)-C(37)-C(38)	106.8(14)
O(4)-C(37)-C(38)	112.7(8)
C(40)-C(37)-C(38)	109.3(4)
C(39)-C(37)-C(38)	107.7(5)
F(6)-C(38)-F(7)	108.9(5)
F(6)-C(38)-F(8)	108.9(5)
F(7)-C(38)-F(8)	107.0(5)
F(6)-C(38)-C(37)	113.8(5)
F(7)-C(38)-C(37)	110.0(5)
F(8)-C(38)-C(37)	108.1(4)
F(9)-C(39)-F(10)	108.1(6)
F(9)-C(39)-F(11)	105.7(6)
F(10)-C(39)-F(11)	109.5(7)
F(9)-C(39)-C(37)	111.9(6)
F(10)-C(39)-C(37)	110.2(5)
F(11)-C(39)-C(37)	111.3(5)
F(13)-C(40)-F(14)	108.8(5)
F(13)-C(40)-F(12)	108.0(5)
F(14)-C(40)-F(12)	104.7(5)
F(13)-C(40)-C(37)	111.4(5)
F(14)-C(40)-C(37)	111.3(5)
F(12)-C(40)-C(37)	112.4(5)
O(5)-C(41)-C(43)	112.4(4)
O(5)-C(41)-C(44)	109.1(4)
C(43)-C(41)-C(44)	109.9(4)
O(5)-C(41)-C(42)	108.6(4)
C(43)-C(41)-C(42)	108.2(4)
C(44)-C(41)-C(42)	108.6(4)
F(15)-C(42)-F(16)	108.4(4)
F(15)-C(42)-F(17)	105.6(5)
F(16)-C(42)-F(17)	108.5(4)
F(15)-C(42)-C(41)	110.8(4)
F(16)-C(42)-C(41)	111.4(4)
F(17)-C(42)-C(41)	111.8(4)
F(20)-C(43)-F(18)	106.6(4)
F(20)-C(43)-F(19)	110.2(5)
F(18)-C(43)-F(19)	105.9(5)
F(20)-C(43)-C(41)	113.6(5)
F(18)-C(43)-C(41)	109.5(4)
F(19)-C(43)-C(41)	110.6(4)
F(22)-C(44)-F(23)	109.8(6)
F(22)-C(44)-F(21)	105.7(5)

F(23)-C(44)-F(21)	108.8(5)
F(22)-C(44)-C(41)	112.1(5)
F(23)-C(44)-C(41)	109.6(5)
F(21)-C(44)-C(41)	110.8(5)
O(6A)-C(45)-C(47)	109.4(5)
O(6)-C(45)-C(47)	111.8(6)
O(6A)-C(45)-C(48)	122.7(6)
O(6)-C(45)-C(48)	88.2(7)
C(47)-C(45)-C(48)	109.2(6)
O(6A)-C(45)-C(46)	97.0(6)
O(6)-C(45)-C(46)	126.6(7)
C(47)-C(45)-C(46)	110.3(6)
C(48)-C(45)-C(46)	107.3(5)
F(25)-C(46)-F(26)	107.2(7)
F(25)-C(46)-F(24)	107.8(6)
F(26)-C(46)-F(24)	105.9(7)
F(25)-C(46)-C(45)	113.0(7)
F(26)-C(46)-C(45)	111.3(6)
F(24)-C(46)-C(45)	111.3(6)
F(28)-C(47)-F(29)	108.1(7)
F(28)-C(47)-F(27)	106.6(7)
F(29)-C(47)-F(27)	109.2(6)
F(28)-C(47)-C(45)	112.3(5)
F(29)-C(47)-C(45)	110.3(8)
F(27)-C(47)-C(45)	110.2(7)
F(30)-C(48)-F(31)	107.4(7)
F(30)-C(48)-F(32)	107.5(6)
F(31)-C(48)-F(32)	108.0(8)
F(30)-C(48)-C(45)	112.3(8)
F(31)-C(48)-C(45)	109.9(6)
F(32)-C(48)-C(45)	111.6(6)
O(7)-C(49)-C(52)	93.1(7)
O(7A)-C(49)-C(52)	120.7(6)
O(7)-C(49)-C(51)	102.8(6)
O(7A)-C(49)-C(51)	109.5(5)
C(52)-C(49)-C(51)	112.6(7)
O(7)-C(49)-C(50)	132.5(7)
O(7A)-C(49)-C(50)	96.4(6)
C(52)-C(49)-C(50)	111.6(6)
C(51)-C(49)-C(50)	103.7(6)
F(33)-C(50)-F(34)	112.7(9)
F(33)-C(50)-F(35)	118.2(9)
F(34)-C(50)-F(35)	102.8(7)
F(33)-C(50)-C(49)	110.0(6)
F(34)-C(50)-C(49)	107.1(8)

F(35)-C(50)-C(49)	105.2(8)
F(36)-C(51)-F(37)	106.9(7)
F(36)-C(51)-F(38)	109.8(7)
F(37)-C(51)-F(38)	108.8(7)
F(36)-C(51)-C(49)	114.6(7)
F(37)-C(51)-C(49)	111.1(6)
F(38)-C(51)-C(49)	105.7(6)
F(41)-C(52)-F(40)	106.7(10)
F(41)-C(52)-F(39)	108.4(9)
F(40)-C(52)-F(39)	113.1(9)
F(41)-C(52)-C(49)	109.7(9)
F(40)-C(52)-C(49)	112.0(8)
F(39)-C(52)-C(49)	106.8(8)
C(1X)#1-C(1X)-Cl(1X)	112(2)
C(1X)#1-C(1X)-H(1X1)	109.2
Cl(1X)-C(1X)-H(1X1)	109.2
C(1X)#1-C(1X)-H(1X2)	109.2
Cl(1X)-C(1X)-H(1X2)	109.2
H(1X1)-C(1X)-H(1X2)	107.9
C(1Y)#1-C(1Y)-Cl(1X)	113.0(19)
C(1Y)#1-C(1Y)-H(1Y1)	109
Cl(1X)-C(1Y)-H(1Y1)	109
C(1Y)#1-C(1Y)-H(1Y2)	109
Cl(1X)-C(1Y)-H(1Y2)	109
H(1Y1)-C(1Y)-H(1Y2)	107.8

Table S11. Anisotropic displacement parameters (Å^2 x 10^3) for **Mo-8**. The anisotropic displacement factor exponent takes the form: $-2\pi^2$ [h^2 a^{*2} U_{11} + ... + 2 h k a* b* U_{12}].

	U11	U22	U33	U23	U13	U12
Mo(1)	23(1)	19(1)	18(1)	-3(1)	-1(1)	-1(1)
O(1)	24(1)	31(1)	36(1)	-14(1)	1(1)	-3(1)
N(1)	29(2)	21(1)	26(1)	-6(1)	3(1)	-6(1)
C(1)	24(2)	18(2)	21(2)	-4(1)	0(1)	-1(1)
F(1)	75(2)	54(2)	41(2)	-3(1)	0(1)	-23(2)
O(2)	37(2)	29(1)	31(1)	-6(1)	-12(1)	9(1)
N(2)	33(2)	24(2)	25(1)	-7(1)	6(1)	-7(1)
C(2)	47(2)	29(2)	39(2)	-15(2)	6(2)	-17(2)
F(2)	236(6)	57(2)	41(2)	16(2)	-19(3)	-33(3)

O(3)	28(1)	35(1)	24(1)	-12(1)	0(1)	-4(1)
C(3)	56(3)	32(2)	36(2)	-19(2)	9(2)	-16(2)
F(3)	304(8)	56(2)	89(3)	-18(2)	-129(4)	62(3)
C(4)	24(2)	23(2)	25(2)	-2(1)	0(1)	-7(1)
F(4)	162(5)	106(3)	162(5)	-83(3)	-136(4)	95(3)
C(5)	29(2)	29(2)	38(2)	4(2)	-7(2)	-6(2)
F(5)	54(2)	90(3)	97(3)	-51(2)	-31(2)	31(2)
C(6)	41(2)	56(3)	36(2)	19(2)	-5(2)	-13(2)
C(7)	36(2)	70(3)	37(2)	8(2)	6(2)	-14(2)
C(8)	24(2)	52(3)	45(2)	-6(2)	2(2)	-3(2)
C(9)	26(2)	26(2)	33(2)	-4(1)	-4(1)	-2(1)
C(10)	37(2)	34(2)	61(3)	3(2)	-18(2)	2(2)
C(11)	54(4)	141(7)	50(3)	19(4)	19(3)	-23(4)
C(12)	39(2)	34(2)	48(2)	-1(2)	-18(2)	5(2)
C(13)	34(2)	23(2)	22(2)	-7(1)	7(1)	-7(1)
C(14)	39(2)	32(2)	25(2)	-13(2)	6(2)	-3(2)
C(15)	32(2)	44(2)	39(2)	-23(2)	9(2)	-7(2)
C(16)	53(3)	29(2)	39(2)	-15(2)	21(2)	-14(2)
C(17)	56(3)	31(2)	28(2)	0(2)	8(2)	-5(2)
C(18)	41(2)	29(2)	27(2)	-8(1)	3(2)	-5(2)
C(19)	44(3)	69(3)	35(2)	-5(2)	2(2)	14(2)
C(20)	68(4)	43(3)	74(4)	-17(3)	38(3)	-27(3)
C(21)	49(3)	53(3)	41(2)	-3(2)	-12(2)	4(2)
C(22)	64(3)	24(2)	38(2)	-10(2)	-29(2)	11(2)
C(23)	93(4)	28(2)	37(2)	-4(2)	-30(2)	-3(2)
C(24)	161(7)	33(3)	40(3)	0(2)	-38(4)	-7(4)
C(25)	182(9)	39(3)	61(4)	-18(3)	-77(5)	30(4)
C(26)	135(7)	53(3)	95(5)	-43(4)	-89(5)	54(4)
C(27)	72(4)	45(3)	69(3)	-30(3)	-48(3)	27(3)
C(28)	27(2)	20(2)	23(2)	-4(1)	-5(1)	-2(1)
C(29)	43(2)	20(2)	29(2)	0(1)	-12(2)	-5(2)
C(30)	85(4)	28(2)	28(2)	4(2)	-6(2)	-7(2)
C(31)	73(3)	20(2)	36(2)	-6(2)	-14(2)	1(2)
C(32)	58(3)	33(2)	78(4)	1(2)	-40(3)	5(2)
C(33)	35(2)	35(2)	27(2)	-7(2)	-7(2)	-5(2)
C(34)	65(3)	54(3)	38(2)	-23(2)	-4(2)	-15(2)
C(35)	66(3)	71(4)	37(2)	-30(2)	2(2)	-7(3)
C(36)	37(2)	54(3)	32(2)	-22(2)	9(2)	-6(2)
Al(1)	19(1)	20(1)	25(1)	-6(1)	-3(1)	-2(1)
O(4)	53(6)	44(8)	43(7)	-24(6)	-32(4)	20(4)
O(4A)	94(7)	44(7)	41(6)	-23(5)	-17(5)	-11(5)
O(5)	39(2)	58(2)	88(3)	-30(2)	-21(2)	28(2)
F(6)	89(3)	61(2)	101(3)	-33(2)	28(2)	12(2)
O(6)	28(4)	39(4)	64(7)	-8(4)	-7(3)	-11(3)
O(6A)	40(3)	45(3)	47(4)	-4(3)	-5(3)	-26(3)

F(7)	71(2)	69(2)	92(3)	-9(2)	17(2)	-13(2)
O(7)	37(6)	31(5)	34(4)	13(3)	-13(3)	-9(4)
O(7A)	53(4)	45(4)	54(3)	6(2)	-31(3)	-3(3)
F(8)	53(2)	125(4)	88(3)	-8(2)	-30(2)	21(2)
F(9)	83(3)	120(4)	141(4)	-9(3)	-76(3)	13(3)
F(10)	137(4)	56(3)	186(6)	34(3)	-79(4)	0(3)
F(11)	156(5)	146(4)	48(2)	-12(2)	-47(3)	1(4)
F(12)	122(4)	76(3)	116(4)	-46(3)	-23(3)	-33(3)
F(13)	55(2)	102(3)	153(4)	-30(3)	32(3)	-15(2)
F(14)	93(3)	80(3)	87(3)	32(2)	-5(2)	0(2)
F(15)	76(2)	72(2)	44(2)	-1(2)	-1(2)	-24(2)
F(16)	74(2)	46(2)	85(2)	-28(2)	12(2)	-24(2)
F(17)	55(2)	59(2)	139(4)	37(2)	-16(2)	15(2)
F(18)	88(3)	33(2)	88(2)	-7(2)	-11(2)	-18(2)
F(19)	90(3)	85(3)	61(2)	-15(2)	-36(2)	-27(2)
F(20)	37(2)	71(2)	155(4)	-15(2)	-14(2)	-10(2)
F(21)	92(3)	74(2)	91(3)	-40(2)	35(2)	6(2)
F(22)	146(4)	122(4)	55(2)	-33(2)	-41(2)	-27(3)
F(23)	124(4)	82(3)	46(2)	5(2)	8(2)	-20(2)
F(24)	98(3)	75(3)	113(4)	0(2)	-27(3)	-29(2)
F(25)	76(3)	79(3)	119(4)	-39(3)	12(2)	-8(2)
F(26)	98(4)	166(6)	228(7)	-92(5)	-89(4)	8(4)
F(27)	70(3)	92(3)	210(6)	-89(4)	58(3)	-30(2)
F(28)	75(3)	73(3)	183(5)	-66(3)	15(3)	-42(2)
F(29)	70(3)	83(3)	268(8)	-60(4)	-26(4)	28(3)
F(30)	146(5)	80(3)	135(4)	18(3)	55(4)	44(3)
F(31)	137(5)	153(5)	85(3)	-1(3)	24(3)	17(4)
F(32)	43(2)	130(4)	100(3)	-5(3)	0(2)	-13(2)
F(33)	79(3)	133(5)	171(6)	-44(4)	9(3)	-3(3)
F(34)	193(6)	94(4)	157(5)	-71(4)	41(5)	-18(4)
F(35)	308(10)	193(7)	53(3)	-20(3)	-63(4)	-92(6)
F(36)	107(4)	139(4)	88(3)	-43(3)	7(3)	53(3)
C(37)	39(2)	39(2)	37(2)	-18(2)	-15(2)	7(2)
F(37)	147(5)	129(4)	113(4)	26(3)	-42(3)	85(4)
C(38)	62(3)	58(3)	49(3)	-13(2)	2(2)	-6(3)
F(38)	186(5)	111(4)	172(5)	-88(4)	-82(4)	18(4)
C(39)	78(5)	65(4)	88(5)	2(3)	-44(4)	1(3)
F(39)	105(4)	155(5)	127(4)	-63(4)	19(3)	41(3)
C(40)	57(3)	57(3)	59(3)	-13(3)	-6(3)	-5(3)
F(40)	124(5)	146(5)	118(4)	10(4)	25(4)	-48(4)
C(41)	28(2)	31(2)	35(2)	-12(2)	-10(2)	7(2)
F(41)	72(3)	235(8)	278(10)	-98(7)	-86(5)	25(4)
C(42)	39(2)	35(2)	62(3)	-6(2)	-3(2)	-4(2)
C(43)	45(3)	51(3)	68(3)	-1(2)	-17(2)	-14(2)
C(44)	88(5)	60(3)	43(3)	-15(2)	5(3)	-12(3)

C(45)	35(3)	40(3)	126(6)	-29(3)	19(3)	-21(2)
C(46)	49(4)	67(4)	138(7)	-33(4)	-18(4)	-10(3)
C(47)	37(3)	60(4)	188(9)	-58(5)	15(4)	-16(3)
C(48)	68(4)	77(5)	95(5)	-11(4)	22(4)	0(4)
C(49)	71(3)	52(3)	30(2)	-6(2)	-14(2)	29(3)
C(50)	124(8)	116(7)	83(6)	-56(5)	-20(5)	15(6)
C(51)	88(4)	85(4)	73(3)	-13(3)	-21(3)	30(3)
C(52)	109(5)	120(5)	117(5)	-13(4)	-22(4)	5(4)
C(1X)	49(9)	135(12)	154(15)	-58(10)	-33(8)	11(9)
C(1Y)	48(9)	136(12)	151(15)	-60(10)	-37(8)	13(9)
Cl(1X)	64(1)	163(2)	134(2)	-67(2)	-37(1)	32(1)
